# Cervicovaginal Microbiome and HPV: A Standardized Approach to 16S/ITS NGS and Microbial Community Profiling for Viral Association

**DOI:** 10.3390/ijms26168090

**Published:** 2025-08-21

**Authors:** Jane Shen-Gunther, Qingqing Xia, Hong Cai, Yufeng Wang

**Affiliations:** 1 Gynecologic Oncology & Molecular Medicine, Department of Molecular Medicine, University of Texas Health Science Center at San Antonio, San Antonio, TX 78229, USA; 2 Department of Clinical Investigation, Brooke Army Medical Center, Fort Sam Houston, San Antonio, TX 78234, USA; qingqing.xia.civ@health.mil; 3 Department of Molecular Microbiology and Immunology, South Texas Center for Emerging Infectious Diseases, University of Texas at San Antonio, San Antonio, TX 78249, USA; hong.cai@utsa.edu (H.C.); yufeng.wang@utsa.edu (Y.W.)

**Keywords:** 16S rRNA sequencing, bioinformatics, cervical cancer, human papillomavirus, ITS sequencing, next-generation sequencing, taxonomic classification, vaginal microbiome

## Abstract

16S rRNA next-generation sequencing (NGS) has significantly advanced cervicovaginal microbiome profiling, offering insights into the relationship between vaginal dysbiosis and HPV-associated carcinogenesis. However, reliance on a limited set of 16S hypervariable regions introduces inherent biases that impact results. This study developed standardized workflows for 16S/ITS NGS, with a focus on identifying methodological biases that influence microbial abundance and taxonomic specificity. Commercial NGS tools were employed, including the 16S/ITS QIAseq V1–V9 screening panel, ATCC vaginal microbial standard, and CLC Genomics Workbench integrated with a customized database (VAGIBIOTA) for analysis. The microbial communities of 66 cervical cytology samples were characterized. Among the regions tested, V3V4 exhibited the least quantitative bias, while V1V2 offered the highest specificity. Microbial profiles and Community State Types (CST) (I–V) were broadly consistent with prior studies, with *Lactobacillus* abundance clustering into three states: *L.*-dominant (CST I–III, V), *L.*-diminished (CST IV-A), and *L.*-depleted (CST IV-B). Differential abundance analysis revealed that anaerobic opportunistic pathogens dominant in CST IV-B (dysbiosis) were also enriched in HSIL and HPV-16 positive samples. Our findings revealed distinct differences in species identification across 16S rRNA hypervariable regions, emphasizing the importance of region selection in clarifying microbial contributions to HPV-associated carcinogenesis.

## 1. Introduction

*Gardnerella vaginalis* was first isolated by Leopold in 1953 and later ascribed as the “causative agent” in bacterial vaginitis (BV) by Gardner and Dukes in 1955 [1,2,3,4,5]. They famously described this common gynecologic infection as producing a “gray, thin, homogeneous, odorous, and less acidic” vaginal discharge [2,6]. Over the years, *G. vaginalis* has become central to our understanding of BV, with a global prevalence rate of 23–29% among women of reproductive age [6]. Studies have shown that *G. vaginalis* can form biofilms that promote the growth of a polymicrobial community and contribute to vaginal dysbiosis by displacing the usually dominant *Lactobacillus* species [7,8,9,10]. In 2011, Ravel et al. advanced our understanding of the vaginal microbiome by introducing the Community State Types (CST) classification [11]. This system emerged from an extensive analysis of vaginal samples from asymptomatic North American women using 16S rRNA gene pyrosequencing targeting the V1V2 hypervariable regions [11]. Their groundbreaking study categorized the vaginal microbiome into five distinct microbial clusters, known as CST, each characterized by the dominance of specific *Lactobacillus* species: CST I (*Lactobacillus crispatus*), CST II (*Lactobacillus gasseri*), CST III (*Lactobacillus iners*), CST IV (diverse anaerobic bacteria dominated by *G. vaginalis* with depleted *Lactobacillus* species), and CST V (*Lactobacillus jensenii*) [11]. This classification not only transformed our understanding of microbial ecology but also elucidated the adverse sequela of dysbiosis, including increased susceptibility to sexually transmitted infections (STIs), Human Immunodeficiency Virus (HIV), Human Papillomavirus (HPV), and preterm birth, all of which impose significant burdens on global health [6]. Further studies refined this classification and revealed correlations between CST IV and adverse health outcomes, highlighting the importance of a stable, *Lactobacillus*-dominant microbiome [12,13,14,15,16,17,18,19]. Since its introduction, the CST classification has become a crucial framework for exploring the relationship between vaginal dysbiosis and various gynecological conditions [6,12,13,14,15,16,17,18,19].

Vaginal dysbiosis has emerged as a significant factor influencing the persistence and progression of human papillomavirus (HPV)-associated cervical dysplasia and cancer [20,21,22,23,24,25]. Dysbiosis fosters a pro-inflammatory environment fueled by cytokines and immune cell recruitment, which damages the cervical epithelium to promote viral persistence and integration [5,20,21,22,23,24,25,26]. Dysbiosis extends beyond mere susceptibility to HPV infection; it induces chronic inflammation, which is exacerbated by *G. vaginalis*-mediated cytolysis and cytokine responses [4,5,26]. Inflammation-induced carcinogenesis has long been acknowledged as a complex, multifactorial process [27]. Chronic inflammation creates a tumorigenic environment through the combined actions of pro-inflammatory cytokines, oxidative stress, and altered cellular signaling pathways [28,29,30,31]. At the molecular level, inflammatory stimuli trigger immune cells like macrophages and neutrophils to generate reactive oxygen and nitrogen species. This leads to oxidative DNA damage and genomic instability, making tissues more susceptible to malignant transformation [28,29,30,31]. Mechanistically, nitric oxide, superoxide, and other free radicals can induce point mutations and epigenetic modifications that impact critical tumor suppressor genes and oncogenes [30,31]. Collectively, the inflammatory mediators, genomic damage, and disrupted signaling pathways drive the process of carcinogenesis [27,28,29,30,31].

Metagenomic 16S rRNA gene sequencing has been crucial for investigating vaginal microbial communities [11,13]. However, focusing solely on 1–2 sets of 16S hypervariable regions (e.g., V1V2 or V3V4) introduces several inherent biases and limitations that can affect research results and their interpretations [32,33,34,35,36]. One major bias is primer specificity (primer bias), where the choice of primers can preferentially amplify certain taxa over others [32,33,34,35,36]. This may result in certain microorganisms being under- or overrepresented, distorting conclusions about the community structure [32,33,34,35,36]. Commonly used primers may not effectively target all relevant vaginal microbial taxa, resulting in incomplete assessments of microbial diversity [32,33,34,35,36]. Moreover, the V3V4 region might lack the resolution needed to distinguish closely related bacterial species, especially within the *Gardnerella* genus, potentially resulting in misidentification [37]. Existing studies on the performance of different 16S rRNA hypervariable regions for vaginal microbiota are limited and methodologically diverse [32,33,34,35,36]. To address this gap, we evaluated six 16S rRNA regions (V1V2, V2V3, V3V4, V4V5, V5V7, V7V9) for sequencing accuracy using both a mock microbial standard and cervical cytology samples. To ensure quality, reproducibility, and applicability, we utilized widely accessible, commercial off-the-shelf next-generation sequencing (NGS) products, including the 16S/ITS QIAseq V1–V9 screening panel, ATCC vaginal microbial genomic standard, and CLC Genomics Workbench for bioinformatics analysis [38,39,40].

## 2. Results

### 2.1. Taxonomic Classification and Visualization of Cervicovaginal Microbiomes

A total of 70 unique clinical samples and 2 (replicate) ATCC vaginal microbial genomic standards were submitted for NGS. Four clinical samples (ID nos.: 30663-026, -031, -035, -048) failed the library preparation with very low DNA concentrations within the 600–670 bp range and were excluded from downstream sequencing. The remaining 66 cytology samples formed the clinical NGS dataset, classified as Negative for Intraepithelial Lesion or Malignancy (NILM) (n = 10) (ID no.: 30856-001 to -012), Atypical Squamous Cells of Undetermined Significance (ASCUS) (n = 12) (ID no.: 30856-012 to -022), Low-grade Squamous Intraepithelial Lesions (LSIL) (n = 24) (ID no.: 30663-001 to -024), and High-grade Squamous Intraepithelial Lesions (HSIL) (n = 20) (ID no.: 30663-025 to -048 exclusive of the 4 failed samples). The replicate vaginal microbiome standards (n = 2) (ID no.: 30856-023 to -024) were sequenced alongside the clinical samples.

**Figure 1:**
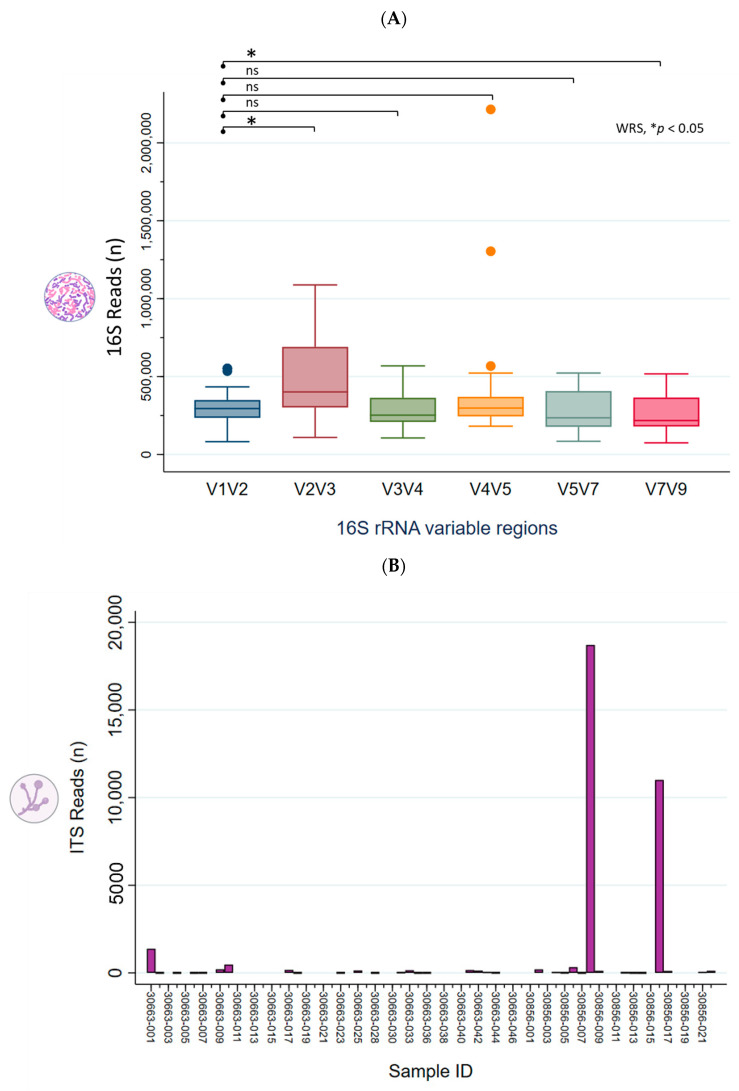
16S/ITS NGS read counts. (**A**) Boxplot showing read count distribution across 16S hypervariable regions for 66 clinical samples. With V1V2 as the comparator, V2V3 showed higher and V7V9 lower read counts (Wilcoxon rank-sum test, * *p* < 0.05), suggesting primer bias. Outliers are plotted as points beyond the whiskers. ns, not significant. (**B**) ITS read counts across 66 samples had a median of 2 (range: 0–18,708) with a right-skewed distribution; only 15 samples had sufficient reads (≥100) for downstream taxonomic profiling.

Following sequencing, the NGS reads (FASTQ files) were demultiplexed using the CLC QIAseq 16S/ITS demultiplexer tool to generate sequence lists corresponding to each specific 16S/ITS region. Figure 1A displays a boxplot illustrating the distribution of read counts across the 16S hypervariable regions (V-regions) for all 66 clinical samples. Median read counts for the V-regions (V1V2, V2V3, V3V4, V4V5, V5V7, V7V9) were 293,288; 394,547; 250,331; 289,505; 233,063, and 215,039, respectively. Using V1V2 as the reference, the V2V3 region exhibited significantly higher read counts, whereas V7V9 showed lower counts (Wilcoxon rank-sum test, * *p* < 0.05), indicating primer bias. Figure 1B depicts the right-skewed distribution of ITS read counts across 66 samples, with a median of 2 (range: 0 to 18,708). Only 15 samples had sufficient read counts (≥100) for downstream analysis.

The demultiplexed 16S/ITS reads from each sample were processed through the “Data Quality Control (QC) and Taxonomic Profiling” workflow, which generated two key outputs: (1) a graphical QC report and supplementary QC report, and (2) an Abundance Table. The graphical report provided a summary of the total number of sequences and nucleotides per sample, along with analyses such as per-sequence and per-base metrics. Overall, forward and reverse reads showed high-quality scores (PHRED ≥ 30) across all samples, with R1 ≥ 94% and R2 ≥ 80% of reads scoring between 30 and 38. Due to the large size of the QC reports, a representative sample (30856-002) is included in Supplementary Table S1. Detailed QC metric descriptions are available in the CLC Microbial Genomics Module manual [41]. The taxonomic profiling workflow generated individual abundance tables that display the names of the identified taxa, 7-level taxonomic nomenclature, coverage estimate, and abundance value (raw or relative number of reads found in the sample associated with the taxon). The merged abundance tables, which compile data across all samples, include summary statistics such as total reads per taxon, as well as the minimum, maximum, mean, median, and standard deviation (see Supplementary Table S2). The metadata is presented separately in Supplementary Table S3. Graphical representations of these tables are described below.

**Figure 2:**
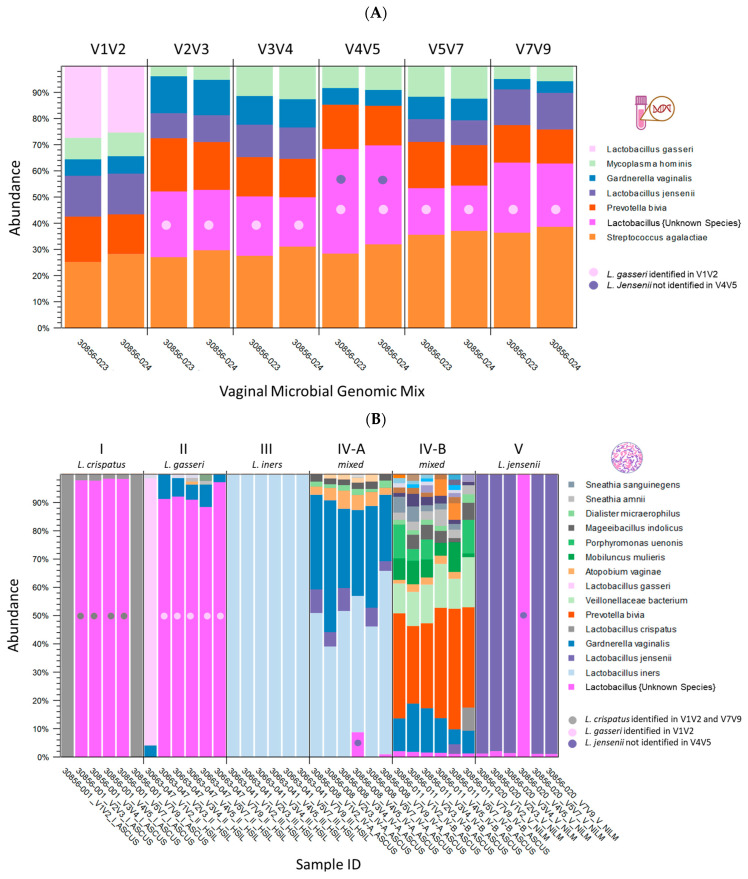
Taxonomic profiles of the ATCC vaginal microbiome standard and six cytology samples representing Community State Types (CST) I-V. (**A**) Six microbial species in the ATCC standard (each at 16.7%) were sequenced in replicate across V-regions and shown as stacked bars. Species-level specificity varied by region: only V1V2 detected *L. gasseri*, while V4V5 missed *L. jensenii*. V3V4 showed the most balanced representation across replicates. (**B**) Stacked bar plots show taxonomic profiles from six cytology samples representing CST I–V, each dominated, respectively, by *L. crispatus*, *L. gasseri*, *L. iners*, mixed anaerobic bacteria, and *L. jensenii*. Species-level detection varied by V-region: *L. crispatus* (CST I) was identified only by V1V2 and V7V9; *L. gasseri* (CST II) only by V1V2; *L. iners* (CST III) was consistently detected across all regions; and *L. jensenii* (CST IV-A and V) was absent in V4V5.

The taxonomic profile of the ATCC vaginal microbiome standard is illustrated in Figure 2A. The stacked bars represent six microbial species included in the ATCC standard, each contributing equally at 16.7%, sequenced in replicates across various V-regions. Species-level specificity varied depending on the V-region sequenced; notably, *L. gasseri* was identified only in V1V2, while *L. jensenii* was not detected in V4V5. Among the sequenced regions, V3V4 exhibited the least bias in the proportions of the six microbial species (chi-square, *p*-value > 0.05) and demonstrated high reproducibility across technical replicates (chi-square, *p*-value > 0.05). Figure 2B presents the taxonomic profiles of six cytology samples representing CST I-V, dominated by *L. crispatus*, *L. gasseri*, *L. iners*, mixed anaerobic bacteria, and *L. jensenii*, respectively. Variations in species-level specificity based on the targeted V-region were observed in clinical samples. In CST I, *L. crispatus* was uniquely identified in the V1V2 and V7V9 regions. For CST II, *L. gasseri* was detected only in the V1V2 region, whereas *L. iners* was identified across all V-regions in CST III. In CST IVA and V, *L. jensenii* was found in all regions except for V4V5.

**Figure 3:**
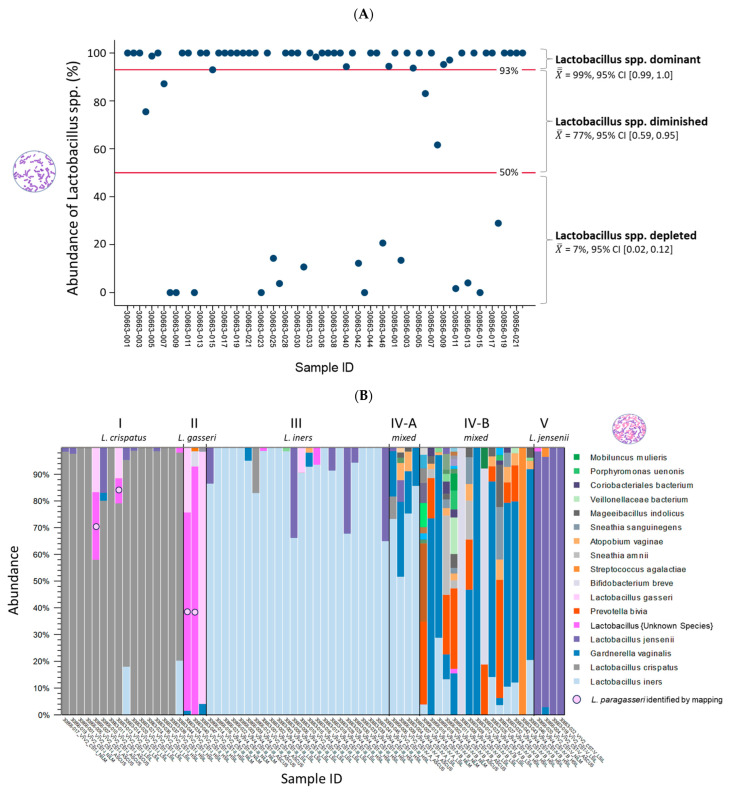

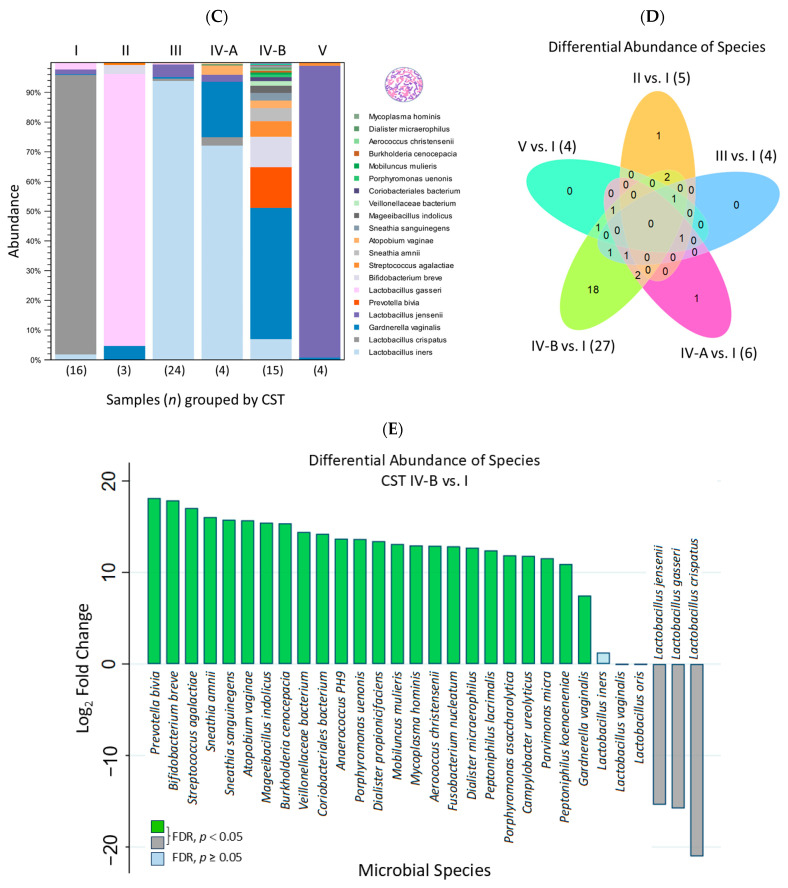
Cervicovaginal microbiome profiles of 66 cytology samples. (**A**). The dot plot shows *Lactobacillus* abundance per sample. Clustering identified three *Lactobacillus* states: *L.*-dominant (CST I–III, V), *L.*-diminished (IV-A), and *L.*-depleted (IV-B) based on abundance levels. Mean values with 95% CI and red reference lines distinguish these states. (**B**). The stacked bar chart shows microbial composition (%) per sample, based on the proportion of mapped reads. Of 30 species identified by 16S sequencing, the top 17 are shown in the legend. For each sample, the V-region with the best species-level resolution is displayed. (**C**) The merged stacked bar chart shows microbial composition by CST, highlighting the dominant species within each group. (**D**) The Venn diagram compares species abundance across CST, using CST I as the reference. Unique species (n) in CST II–V appear in the arms of the diagram, with those showing an absolute fold change > 1.5 noted in parentheses. (**E**) The bar chart highlights the significant shift in species (n = 27) for CST IV-B compared to CST I, marked by an enrichment of facultative and/or anaerobic organisms (green bars) and depletion of *L. crispatus*, *gasseri*, and *jensenii* (gray bars).

Following taxonomic profiling, ambiguous *Lactobacillus* reads were resolved as *L. paragasseri* via read mapping. *Lactobacillus* abundance (%) was then quantified per sample. Partition clustering grouped samples into three *Lactobacillus* states: *L.*-dominant (CST I–III, V), *L.*-diminished (CST IV-A), and *L.*-depleted (CST IV-B), based on *Lactobacillus* levels (Figure 3A). Mean abundances with 95% confidence intervals distinguished the CST, with reference lines marking 93% (*L.*-dominant threshold) and 50% (*L.*-depleted threshold).

Figure 3B displays a stacked bar chart of microbial species abundance per sample. To ensure species-level accuracy, the most suitable V-region was selected for each sample. The bar charts for species-level abundance across V1-V9 regions for individual samples are provided in Supplementary Figure S1. 16S V-region sequencing detected 30 unique species, with the top 17 highlighted in the legend and their composition (%) calculated based on the proportion of mapped reads (n) to the total mapped reads (Figure 3B).

The CST distribution across the 66 samples included CST I (24%), II (5%), III (36%), IV-A (6%), IV-B (23%), and V (6%). Among the samples, *L. iners* was the most abundant species, particularly in CST III and IV-A. Four samples (30856-007 and 30663-013, -034, and -040) containing ambiguous *Lactobacillus* reads were identified as *L. paragasseri* by read mapping (Figure 3B). Figure 3C presents a merged stacked bar chart displaying the 66 samples grouped by CST. Grouping clearly highlighted the dominant species within the mixed microbial communities of CST IV-A and IV-B, identified as *L. iners* and *G. vaginalis*, respectively.

The differential abundance of species across CST groups is depicted in a Venn diagram (Figure 3D). Using the taxonomic profiling table as input and CST I as the reference, pairwise comparisons identified species unique to CST II–V, as well as those meeting the criteria of an absolute fold change greater than 1.5 and a false discovery rate (FDR) *p*-value < 0.05 (Supplementary Table S4). Unique species are displayed in the arms of the Venn diagram, while those with significant fold changes are indicated in parentheses. Notably, CST IV-B exhibited the highest number of unique species (n = 18) and the greatest number of species with significant fold changes (n = 27) compared to CST I (Figure 3E).

**Figure 4:**
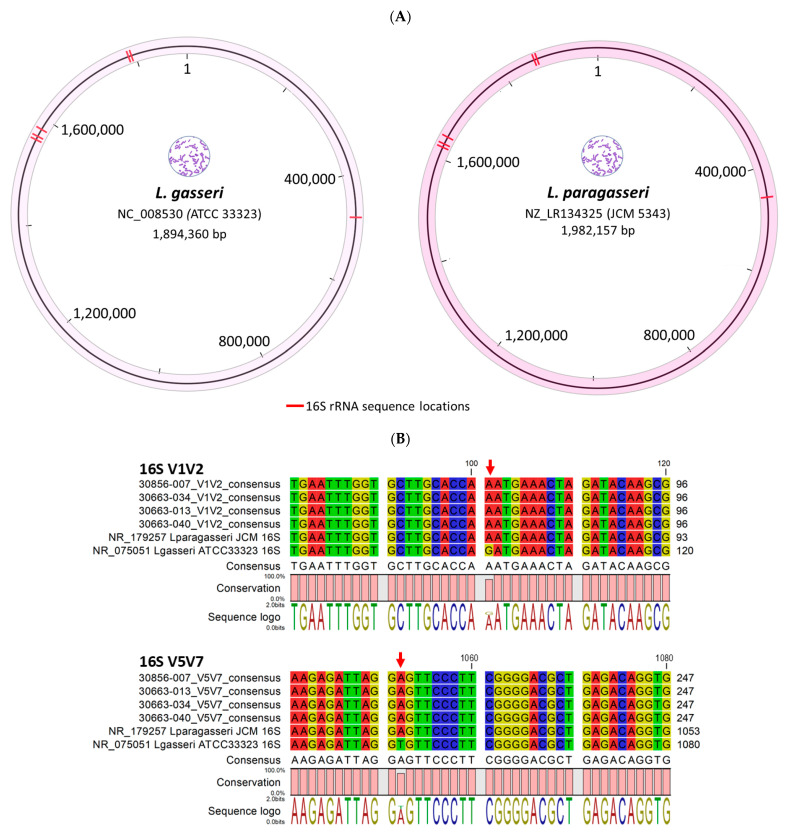
Alignment of reads classified as “*Lactobacillus* spp. unknown” to *L. paragasseri* and *L. gasseri* 16S rRNA genes. (**A**) Representative *L. gasseri* and *L. paragasseri* genomes show multiple 16S loci, with ambiguous reads mapping to expected locations. (**B**) In four samples containing ambiguous *Lactobacillus* spp. reads (30856-007; 30663-013, -034, -040), the V1V2 and V5V7 consensus sequences matched *L. paragasseri* (NR179257), distinguished from *L. gasseri* (NR975051) by SNPs at positions 101 (G/A) and 1052 (T/A), marked by red arrows.

Reads classified as “*Lactobacillus* spp. unknown” by taxonomic profiling were resolved by read mapping. The reads were initially extracted from the taxonomic profiling output and subsequently processed through the Read Mapping workflow to generate a tabulated report. The ambiguous *Lactobacillus* reads (n, %) identified in four samples: 30856-007 (134,698; 25%) and 30663-013 (31,659; 9%), -034 (253,079; 74%), and -040 (146,466; 99%) were mapped to *L. paragasseri* and *L. gasseri*. The read proportions for *L. paragasseri* and *L. gasseri* varied between 51 and 56% and 44 and 49%, respectively. Furthermore, the ambiguous *Lactobacillus* reads mapped to one or more of the expected 16S rRNA genome locations in *L. paragasseri* and *L. gasseri*, as illustrated in Figure 4A. The inability of taxonomic profiling to differentiate and classify these reads is due to the nearly identical 16S rRNA sequences (99.9%) of *L. gasseri* and *L. paragasseri* [42,43]. Ene et al. found that the two species cannot be distinguished by short-read sequencing of the 16S rRNA gene, as they differ only by two nucleotides at positions 95 (C/T) in the V1 region and 1046 (A/T) in the V6 region [43]. Additionally, these positions and nucleotides may vary between unique strains of *L. paragasseri* and *L. gasseri* [43]. In our samples, V1V2 and V5V7 consensus sequences (derived from mapped reads) aligned with *L. paragasseri* (NR179257) over *L. gasseri* (NR975051). This alignment was confirmed by distinguishing nucleotides at positions 101 (G/A) and 1052 (T/A) (Figure 4B).

Fungal species were generally rare and detected at very low read abundance. Taxonomic analysis of ITS sequences identified *Candida albicans* as the sole fungal species in 7 of 66 samples (11%). The sample IDs along with their corresponding CST were as follows: 30663-001 (III), 30663-010 (III), 30856-002 (IV-B), 30856-006 (IV-A), 30856-008 (IV-A), 30856-009 (III), and 30856-017 (I). The respective mapped read counts for these samples were 1136; 462; 194; 316; 10,911; 88; and 112, as illustrated in Supplementary Figure S2 (bar chart). Only sample 30856-008 (CST IV-A) exhibited a significantly elevated ITS read count compared to all other samples.

### 2.2. Diversity Analysis and Visualization of Microbial Communities

**Figure 5:**
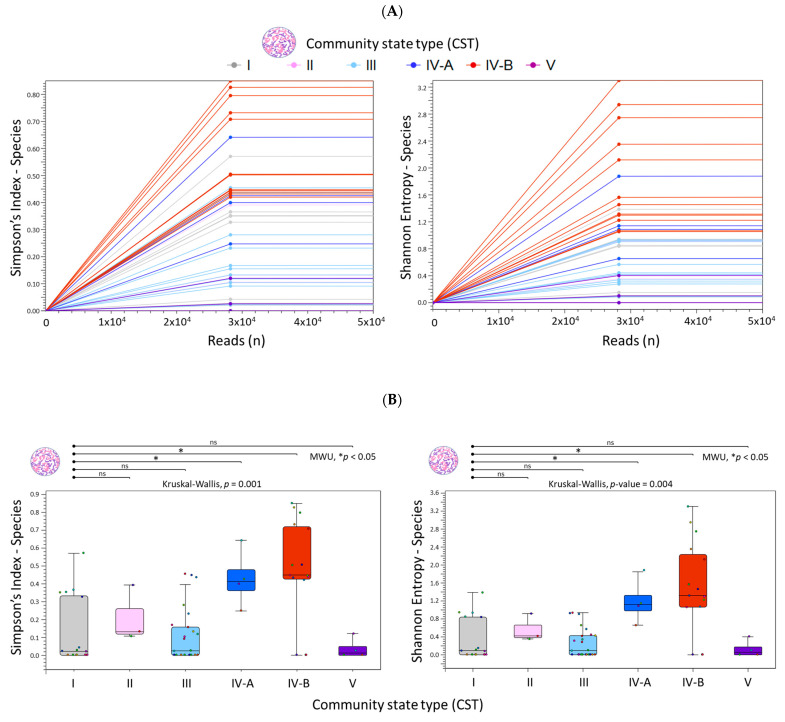
Diversity analysis of microbial species in liquid cytology samples by Community State Types (CST). (**A**) Simpson and Shannon rarefaction curves plateaued at 28,000 reads, indicating sufficient sampling depth across all 66 samples. (**B**) Boxplots summarize CST-grouped samples. Colored points represent individual samples. Species richness (Simpson’s index) and evenness (Shannon entropy) significantly increased from *Lactobacillus*-dominant (CST I–III, V) to *L.*-diminished and *L.*-depleted states (CST IV-A, IV-B). MWU: Mann–Whitney U test. ns, not significant.

The “Merge and Estimate Alpha and Beta Diversities” workflow produced the following outputs: (1) an alpha diversity rarefaction table and plots, and (2) a beta diversity distance matrix alongside principal coordinate analysis (PCoA) plots. The alpha diversity metric is calculated by sub-sampling abundances at varying depths (number of reads) for each sample. As illustrated in Figure 5A, the rarefaction curves for Simpson’s and Shannon indices reached a plateau at 28,000 reads, signifying adequate sampling depth. This indicates that at 28,000 reads, most species have been captured, with minimal gain from further sampling. Figure 5B presents box plots for grouped samples classified by CST, accompanied by the auto-calculated statistical results. Notably, CST IV-A and IV-B demonstrated significantly higher species richness (Simpson’s index) and species evenness (Shannon entropy) in comparison to the reference group, CST I.

**Figure 6:**
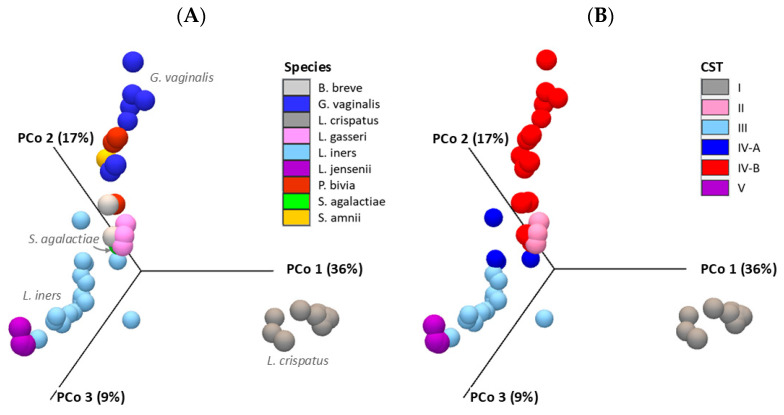
Cervicovaginal microbial community structures. (**A**) 3D PCoA plot of 66 samples (color-coded by species) highlights *L. crispatus*, *G. vaginalis*, and *L. iners* as key drivers of microbial variation. (**B**) 3D PCoA plot grouped by CST shows CST I, IV-B, and III as major contributors to variance (36%, 17%, and 9%, respectively). β-diversity was assessed using Bray–Curtis (PERMANOVA, *p* < 0.05).

The beta diversity analysis produced a Bray–Curtis distance matrix and a visual representation in the form of a 3D PCoA plot, where each sample is represented as a point in three-dimensional space. The Bray–Curtis statistic quantifies the compositional dissimilarity between samples. The PCoA plot (Figure 6) highlights the dissimilarities in microbial composition across all samples, with *L. crispatus*, *G. vaginalis*, and *L. iners* identified as the key species influencing these differences, as represented along the principal coordinates. When grouped by CST, the most influential microbial communities were CST I, IV-B, and III, accounting for 36%, 17%, and 9% of the variance, respectively, as illustrated in Figure 6B.

### 2.3. Differential Abundance Analysis and Visualization Using Hierarchical Clustering Heatmaps

**Figure 7:**
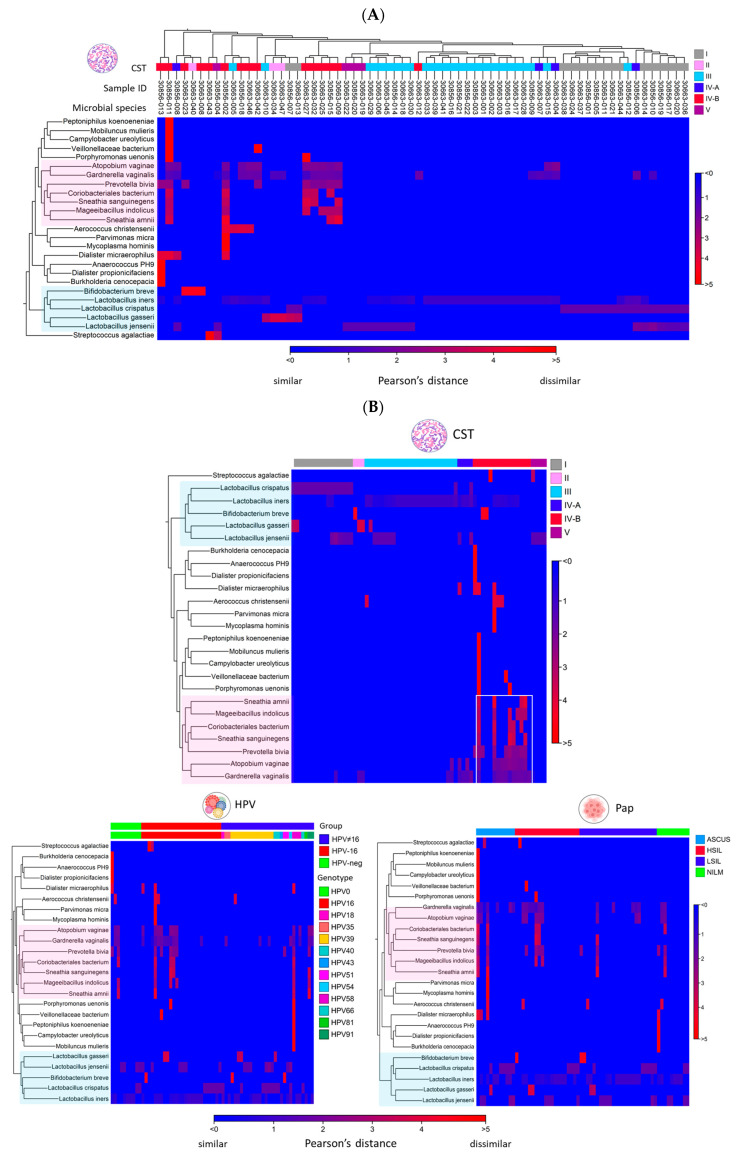
Clustered Heatmap of Microbial Abundance in Liquid Cytology Samples. (**A**) Two-way hierarchical clustering of 66 samples and microbial species reveals distinct CST patterns. *L. iners* formed the earliest clusters, with a shift toward a diverse, anaerobic community in CST IV-B. *Lactobacillus* species (blue overlay) cluster with CST I–III and V, while anaerobes (pink overlay) associate with CST IV-A and IV-B. Differences in species abundance are quantified using Pearson’s distance metric. (**B**) Aggregated heatmaps grouped by CST, HPV type/status and Pap smear diagnosis highlight distinct microbial signatures. Notably, the seven most abundant anaerobic pathogens (pink overlay) in CST IV-B (rectangular outline) are also enriched in HPV-16 positive and HSIL samples.

The “Create Heat Map for Abundance Table” workflow generated a two-way hierarchical clustering heatmap, organizing both the rows (samples) and columns (species) based on their similarity (Figure 7A). The accompanying dendrogram illustrates the grouping patterns among items, while the heatmap itself uses a two-color gradient to visually represent variations in species abundance across the dataset. Figure 7A illustrates the clustering of 66 samples based on CST. Samples dominated by *Lactobacillus* spp., characterized by high relative abundance, are distributed along the blue-to-red gradient of the heatmap. In contrast, *Lactobacillus*-depleted samples (CST IV-B) are predominantly located within the red spectrum. To aid interpretation, *Lactobacillus* spp. are highlighted in blue along the y-axis, while major clusters of anaerobic bacteria are highlighted in pink. Figure 7B presents aggregated heatmaps categorized by CST profiles, Pap smear diagnoses, and HPV genotype or status, categorized as HPV-negative, HPV-positive (non-16), and HPV-16. These visualizations enable direct comparison of microbial compositions across groups, revealing that the dominant anaerobic species associated with CST IV-B (vaginal dysbiosis) are also prevalent in samples diagnosed with HSIL and those positive for HPV-16. Furthermore, differential abundance analysis comparing grouped HSIL and NILM (control) samples identified thirteen enriched non-*Lactobacillus* facultative or anaerobic species (log_2_ fold change > 0), five of which were statistically significant (FDR *p*-value < 0.05) (Supplementary Table S5). Similarly, comparison between HPV-16 positive and HPV-negative (control) samples revealed that seven out of ten enriched species reached statistical significance (FDR *p*-value < 0.05) (Supplementary Table S6).

## 3. Discussion

In this study, we developed and tested a standardized workflow for 16S/ITS metagenomic sequencing of CVM. The goal was to detect biases in the workflow that could impact the correlation between microbial communities and HPV. Bias in the 16S rRNA V-region was detected, as revealed by the uneven distribution of metagenomic reads among the six microbial species that were equally represented in the genomic standard. This discrepancy could be attributed to primer or sequencing bias. The amplified and sequenced V3V4 region was the most evenly distributed among all V-regions but failed to detect *L. gasseri*. The V1V2 region detected *L. gasseri*, though the proportions of *G. vaginalis* and *Mycoplasma hominis* were lower compared to other species. Clinical samples further revealed that species detection varied across the V1 to V9 regions. *L. crispatus* was only identified in the V1V2 and V7V9 regions, while *L. jensenii* was detected in all V-regions except V4V5. Finally, ambiguous *Lactobacillus* reads were mapped to *L. paragasseri* due to its nearly identical 16S rRNA sequence to *L. gasseri*, which required analysis using the V1V2 and V5V7 regions. Our results demonstrated inherent amplification biases in 16S rRNA metagenomic sequencing, ranging from non-detection to skewed microbial abundance levels. Therefore, to enhance accuracy, sequencing data from the V1V2 region for species identification and the V3V4 region for abundance estimation were used in a complementary manner for each sample. This multi-V-region approach to microbiome analysis is supported by other researchers in the field [44,45]. In summation, the 16S rRNA screening panel and mock community standard were critical for detecting bias and ensuring accurate microbiome profiling, thereby supporting robust downstream association analyses.

Among the 66 cytology-derived CVM, 30 unique species were identified. The microbial community characteristics and distribution were highly consistent with the CST I-V categories defined by Ravel and Gajer et al. [11,13]. Specifically, the CST distribution across the 66 samples were as follows: CST I (24%), CST II (5%), CST III (36%), CST IV-A (6%), CST IV-B (23%), and CST V (6%). Unique to this study, we clustered the *Lactobacillus* abundance levels within each sample to quantify three *Lactobacillus* states: *L.*-dominant (CST I-III and V), *L.*-diminished (CST IV-A), and *L.*-depleted (CST IV-B). Quantifying *Lactobacillus* states made CST classification objective and readily automatable through algorithmic approaches, in contrast to earlier studies [11,13,14]. For all the *L.*-diminished (CST IV-A) and *L.*-depleted (CST IV-B) samples, the dominant *Lactobacillus* spp. was *L. iners*, while the dominant opportunistic pathogen was *G. vaginalis*. *L. iners* is known to be associated with other opportunistic pathogens and episodes of dysbiosis [46]. A comprehensive review by Vaneechoutte [46] highlighted several unusual characteristics of *L. iners* compared to other commensal *Lactobacillus* spp. to include the lack of D-lactic acid production (resulting in high pH), limited hydrogen peroxide production, production of inerolysin (a cytotoxin), and adherence to vaginal epithelial cells, as well as promotion of adherence by *G. vaginalis* [46]. In contrast, *L. crispatus* is regarded as a key defender against vaginal dysbiosis, characterized by a substantially larger genome and enhanced metabolic capabilities [46,47,48]. It adheres to the vaginal epithelium, promotes competitive exclusion of other bacteria, produces D-lactic acid (lowering vaginal pH), generates hydrogen peroxide, and inhibits opportunistic pathogens [46,47,48]. As for *L. gasseri* and *L. jensenii*, these species are less effective than *L. crispatus* in maintaining eubiosis [46,47,48]. *Bifidobacterium breve* was also detected at low abundances in samples dominated by *Lactobacillus.* Freitas et al. demonstrated that all vaginal *Bifidobacterium* species can produce and tolerate lactic acid and low pH, although only about one-third generate hydrogen peroxide [49]. Consequently, *Bifidobacterium* is also recognized as a contributor to the protective functions of the vaginal microbiome [49].

As *Lactobacillus* dominance declined from the diminished to the depleted state, species diversity, richness, and evenness increased significantly. Essentially, the dominion of *Lactobacillus* within the vagina was eradicated by a plethora of anaerobic, opportunistic pathogens. Beta-diversity analysis revealed three key species associated with the most influential microbial communities across all samples: *L. crispatus* (CST I), *G. vaginalis* (CST IV-B), and *L. iners* (CST III). Within CST IV-B, hierarchical clustering identified five distinct species clusters among the 19 species. The top seven anaerobes (*G. vaginalis*, *P. bivia*, *A. vaginae*, *S. sanguinegens*, *S. amnii*, *M. indolicus*, and *Coriobacteriales bacterium*) were predominantly associated with CST IV-B, HSIL and HPV-16 positive samples. In particular, the three most abundant species (*G. vaginalis*, *Prevotella bivia and Atopobium vaginae*) are well-documented microbial co-occurrents. Di Paola et al. identified sialidase-producing strains of *G. vaginalis* that form biofilms for survival in symbiotic relationships with *Atopobium* and *Prevotella* [50]. Moreover, the sialidase-encoding gene in *G. vaginalis* was significantly more abundant in CST IV-B samples and in samples from the HPV-persistent cohort compared to those in the HPV-cleared group [50]. Mechanistically, the biofilm produced by *G. vaginalis* traps anaerobic pathobionts like *Atopobium* and *Prevotella*, promotes their overgrowth, which leads to inflammation, epithelial breakdown, and HPV persistence [50]. Beyond dysbiosis, Zhou et al. highlighted the pivotal role of inflammation, along with its associated cytokines, signaling pathways, and oxidative/nitrative stress in promoting HPV infection, tumor proliferation, and carcinogenesis [51]. Conversely, HPV infection itself can trigger inflammatory responses, activating macrophages and natural killer (NK) cells, and inducing the release of pro-inflammatory cytokines, thereby contributing to a state of dysbiosis [51]. Together, HPV and dysbiosis work in concert in a milieu of inflammation to promote carcinogenesis.

The facultative and anaerobic pathogens identified in CST IV-B of this study are implicated in clinically significant polymicrobial infections and chronic inflammation, which predisposes host tissues to malignant transformation. The virulence and diseases associated with each infectious agent are highlighted below. First, *G. vaginalis*, *Atopobium vaginae* and *Prevotella bivia* have all been associated with both lower and upper female genital tract infections, including bacterial vaginosis (BV), pelvic inflammatory disease (PID), endometritis, and preterm birth [52,53]. *Sneathia sanguinegens* and *Sneathia amnii* are emerging pathogens connected to BV, cervical intraepithelial neoplasia (CIN), and preterm birth [54,55,56]. *Mageeibacillus indolicus*, a relatively novel member of the order *Clostridiales*, has been isolated from the endometrium of women with PID [57]. *Coriobacteriales bacterium* has been found in higher abundance in BV cases and in the gut and skin microbiota of postmenopausal women with vulvar lichen sclerosus [58,59]. *Porphyromonas uenonis* has been linked to CIN as well as oral and esophageal squamous cell carcinoma [60]. *Mobiluncus mulieris* frequently co-occurs with *Gardnerella* and *Atopobium* in BV cases [61,62]. *Campylobacter ureolyticus* is an underrecognized pathogen implicated in soft tissue infections, abscesses, and infective endocarditis [63,64]. *Peptoniphilus koenoeneniae* is an emerging opportunistic pathogen associated with abscesses and chronic sinusitis [65,66]. In a case–control study of pregnant women, *Veillonellaceae* bacterium was associated with BV and linked to early preterm birth [67]. *Aerococcus christensenii* is a commensal organism isolated from cases of urinary tract infections (UTIs), polymicrobial infections, abscesses, and soft tissue infections [68]. *Parvimonas micra*, another commensal bacterium, has been associated with infective endocarditis, osteomyelitis, empyema, and infections in immunocompromised individuals [69,70,71]. *Mycoplasma hominis* colonizes the urogenital tract and is linked to urethritis, BV, PID, and infertility [72,73]. *Burkholderia cenocepacia* is a well-known opportunistic pathogen capable of causing severe respiratory infections, particularly in immunocompromised patients [74,75]. *Anaerococcus vaginalis* is a biofilm-forming bacterium associated with BV, ovarian abscesses, and other anaerobic infections, often within polymicrobial environments [76]. *Dialister propionicifaciens* has been linked to BV, while *Dialister micraerophilus* has been identified in polymicrobial infections, abscesses, and pyometra [77,78,79]. Finally, Group B Streptococcus (GBS), or *Streptococcus agalactiae*, is a leading cause of invasive infections in newborns, pregnant women, and immunocompromised adults [80,81,82,83,84]. In neonates, it is the primary cause of early-onset sepsis, pneumonia, and meningitis [80,81,82,83,84]. In pregnancy, GBS can lead to UTIs, chorioamnionitis, preterm labor, and postpartum infections [80,81,82,83,84]. In non-pregnant adults, it is linked to bacteremia, skin infections, and endocarditis [84]. Collectively, the opportunistic pathogens identified in CST IV-B have the potential to cause serious infections and substantial morbidity under conducive environmental and host conditions.

Fungal ITS sequencing yielded low read counts, with *Candida albicans* detected in only 7 of 66 samples (11%). This aligns with a recent study using combined 16S rRNA and ITS sequencing, which found ITS reads comprised a small proportion of total sequences (mean 780, 1%) [85]. Notably, *C. albicans* was the predominant fungal species in 39 of 50 vaginal samples from healthy women [85]. Despite growing interest in cost-effective, cross-kingdom microbial profiling, studies on simultaneous DNA extraction and pooled sequencing remain scarce [85]. The low abundance of fungal reads observed here may be attributed to a suboptimal lysis protocol, given the complex and rigid structure of fungal cell walls. Recent efforts have explored enzymatic, thermal, mechanical, and combined lysis strategies to enhance fungal DNA yield while minimizing degradation [86,87,88,89], highlighting the need for further optimization of 16S/ITS co-extraction methods for accurate vaginal mycobiome characterization.

The strength of this study lies in our use of the 16S/ITS screening panel and a streamlined, wet-to-dry lab workflow, which enabled us to identify variable region bias effectively. By employing genomic standards alongside clinical samples, we were able to pinpoint both qualitative and quantitative biases in taxonomic profiling. Additionally, our study characterized three distinct *Lactobacillus* states and identified the dominant pathogenic microbial species associated with the *Lactobacillus*-depleted state (CST IV-B), which may contribute to a microenvironment conducive to carcinogenesis. This comprehensive approach underscores the robustness of our findings in elucidating microbial contributions to HPV-associated carcinogenesis.

We recognize several limitations in our study. First, the vaginal genomic standard included a limited number of microbial species. Expanding this panel to incorporate *L. crispatus*, *L. iners*, *L. paragasseri*, and *Atopobium vaginae* would enhance taxonomic resolution by providing a more comprehensive set of quantifiable reference species, encompassing both commensals and pathogens. In addition, incorporating mock genomic standards as spike-ins on the same flow cell as clinical samples can support calibration and target sequence detection [90,91]. Nonetheless, the risk of cross-contamination into clinical samples warrants consideration. While unique dual indexes enable bioinformatic distinction of synthetic mock standards from clinical reads, experimental validation remains essential [90]. Second, library preparation failed in 4 out of 70 samples due to low 16S rRNA DNA concentration. Possible explanations include technical errors during library preparation or a low microbial-to-host DNA ratio, commonly referred to as “host DNA contamination” [92]. To address this, pre-sequencing enrichment methods or assays, which selectively degrades host DNA while preserving microbial DNA, have been proposed [92]. Additionally, quantifying microbial DNA before and after library preparation using commercial real-time PCR-based assays, such as the Microbial DNA qPCR Assay and the QIAseq Library Quant Assay, can serve as valuable quality control measures [38,93]. Other factors influencing read counts in NGS include primer specificity, microbial genome complexity, sequencing depth, and bioinformatics analysis parameters [35,38]. Thus, identifying and addressing these variables is essential for improving the accuracy and reliability of NGS-based microbial research. Third, we selected the SILVA 99% general-purpose 16S rRNA database due to its strengths, including its comprehensive coverage, regular updates, widespread use in vaginal microbiome research, and high species-level resolution [35,94]. The drawbacks include the substantial size of the SILVA database, which can hinder processing speed, and the presence of redundant or unclassified taxa that may result in ambiguous outcomes. To mitigate these issues, we filtered out irrelevant or unclassified taxa and supplemented the database with taxa known to inhabit the human vagina [95]. Although this study did not compare SILVA and VAGIBIOTA directly, future evaluation of their relative performance would be valuable. Finally, due to the cross-sectional design of our study, we are unable to infer causality between CST IV-B-associated microbial pathogens and HPV infection or high-grade cervical cytopathology. Longitudinal studies are necessary to investigate these associations over time. Such studies would allow for the monitoring of microbial and virological dynamics, such as changes in viral genotype and load within individuals, providing insights into potential causal pathways and mechanisms of disease progression.

## 4. Materials and Methods

### 4.1. Clinical Samples, Mock Genomic Standard and Deep Sequencing

A subset of 70 unique cervical cytology samples classified as NILM (n = 10), ASCUS (n = 12), LSIL (n = 24), and HSIL (n = 24) were randomly selected from a larger (3000+ samples) Congressionally Directed Medical Research Programs (CDMRP) grant-funded project to test our workflow-based methods described herein. The residual liquid-based cervical cytology samples were procured consecutively from the Department of Pathology after completion of cytological diagnosis. Demographic and cytological data were abstracted from the electronic health record (AHLTA) as metadata for association with taxonomic profiling results. The subjects of the current samples had a median age of 28 years, with an interquartile range (IQR) of 24 to 32 years. The HPV deep sequencing data, previously published [96], were utilized to explore associations with the microbiome. Specifically, each sample’s dominant HPV genotype and categorical status (HPV-negative, HPV-16 negative, or HPV-16 positive) were used for analysis.

Cellular DNA was extracted according to the manufacturer’s protocol as previously validated [86,96,97]. Briefly, 10 mL of each liquid-based cytology sample was centrifuged to obtain a 400–500 μL cell pellet, which underwent off-board lysis. The pellet, placed in a 2 mL Sarstedt tube, was incubated with 40 μL of Qiagen proteinase K and 400 μL of Buffer AL at 56 °C and 9000 rpm for 10 min in a thermomixer. The resulting lysate (800–1000 μL) was loaded into a QIAsymphony carrier for automated extraction using the QIAsymphony DSP DNA Midi Kit (96) following the DNA BLOOD 1000 V7 DSP protocol on the QIAsymphony robotic workstation (Qiagen, Germantown, MD, USA) [97]. Purified DNA (200 μL), eluted in a Qiagen 96-well Elution Microtubes plate, was quantified using QIAxpert spectrophotometry (A260 dsDNA protocol) [98] and stored at −20 °C before transfer to −80 °C for long-term storage. DNA purity was determined by the A260:A280 absorbance ratio, with values > 1.8 regarded as high purity [98].

**Figure 8:**
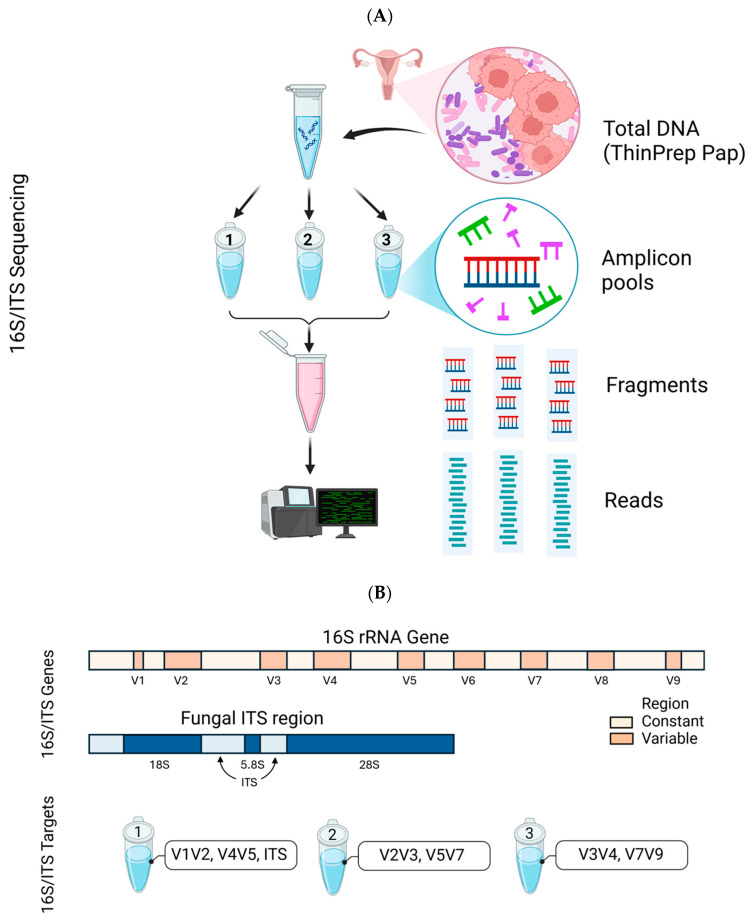
Workflow for 16S/ITS metagenomic sequencing. (**A**) DNA extracted from liquid cytology samples underwent two-step PCR: first, amplification of 16S rRNA and ITS regions using three primer pools; second, barcode addition for sample identification. Libraries were pooled and sequenced on the MiSeq platform. (**B**) The diagram shows bacterial 16S rRNA and fungal ITS gene regions targeted by the QIAseq 16S/ITS Screening Panel, highlighting coverage by three primer pools, represented by PCR tubes in the figure, were used for library enrichment. (Figures created in BioRender).

The extracted DNA from each sample (20 µL with concentration of ≥20 ng/µL) was submitted to Qiagen Genomic Services (QIAGEN, Germantown, MD, USA) for 16S/ITS amplicon NGS. The QIAseq 16S/ITS Screening Panel (Germantown, MD, USA) was used for DNA library target enrichment and downstream microbial classification [38]. Per manufacturer’s protocol, the QIAseq 16S/ITS Panel utilizes a 2-stage PCR workflow briefly described and shown below (Figure 8A). The first round of PCR (12 cycles) amplifies and enriches the 16S rRNA variable (V1V2, V2V3, V3V4, V4V5, V5V7, V7V9) and ITS regions of the extracted DNA. Each PCR reaction uses 1 µL of DNA at a concentration of 1 ng/µL (Figure 8B). The second round of PCR (14 cycles) adds library adapters to the ends of amplicons to serve as barcodes for multiplexing samples. Library QC was conducted using the Agilent^®^ 2100 Bioanalyzer^®^, following the manufacturer’s protocol, to confirm the presence of the expected amplicon peaks for 16S rRNA (~620 bp) and ITS (~440 bp) regions [38]. The QIAseq 16S/ITS Smart Control, a synthetic DNA construct, was included as a separate sample during library preparation to assess library quality and yield [38]. According to the manufacturer, the QIAseq 16S/ITS Panel can detect as little as 1 picogram of microbial DNA and 0.01 nanogram of fungal DNA [38]. The libraries were normalized quantitatively to a concentration of 2 nM for equimolar representation from each sample prior to pooling and sequencing. Paired-end bi-directional sequencing (2 × 300 bp) was performed on the MiSeq instrument (Illumina, San Diego, CA, USA) using the MiSeq Reagent Kit v3 (600-cycles) on a single flow cell for bridge amplification.

The ATCC Vaginal Microbiome Genomic Mix (ATCC MSA-1007) (American Type Culture Collection, Manassas, VA, USA) was used as a mock standard to mimic metagenomic samples and assess PCR and/or sequencing bias [39]. The product includes an even mixture of genomic DNA from six bacteria (16.7% *G. vaginalis* (ATCC 14019D-5), 16.7% *L. gasseri* (ATCC 33323D-5), 16.7% *M. hominis* (ATCC 23114D-5), 16.7% *P. bivia* (ATCC 29303D-5), 16.7% *S. agalactiae* (ATCC BAA-611D-5), and 16.7% *L. jensenii* (ATCC 25258D-5)). The genomic specification range was: 1.2 × 10^8^ genome copies/50 µL vial ± 1 log. Two (10 µL) replicate samples of the ATCC vaginal genomic mix with a DNA concentration of 7.5 ng/µL were submitted for concurrent 16S/ITS NGS as described above.

### 4.2. Customized Vaginal Microbiota Reference Database for CLC Workflows

The comprehensive SILVA rRNA gene database (https://www.arb-silva.de/ (accessed on 12 June 2022)) provides sequences for both small (16S/18S, SSU) and large (23S/28S, LSU) rRNA subunits across Bacteria, Archaea, and Eukarya [94]. The SILVA SSU 99% (v138.1) dataset, comprising 510,508 sequences, was imported into CLC and tailored for use as a reference database for vaginal microbiota studies. The customization process excluded sequences with broad or unclear taxonomic classifications at the species level, filtering out terms like “Archaea”, “Eukaryota”, “Uncultured”, “Unidentified”, and “metagenome”. Furthermore, the top 10 species from vaginal community state types (CST) I-V, as identified by the Human Microbiome Project [95], were included to ensure representation of essential vaginal commensal and pathogenic bacteria. An additional 10 pathogenic bacterial genomes, referenced in the literature, were downloaded from NCBI and incorporated into the database. The final Vaginal Microbiota Reference Database was categorized into three main sequence groups: Lactobacillus (n = 319), non-Lactobacillus (n = 882), and Gonorrhea (n = 31)/Chlamydia (n = 53), encompassing a total of 1284 unique rRNA sequences (Supplementary Table S7). The customized Vaginal Microbiota Reference Database was renamed as VAGIBIOTA for simplicity.

**Figure 9:**
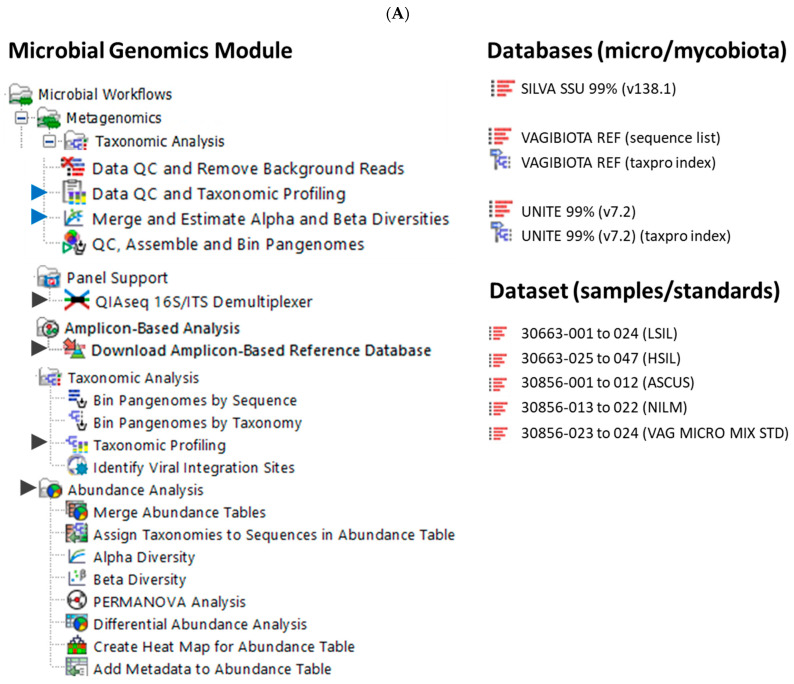

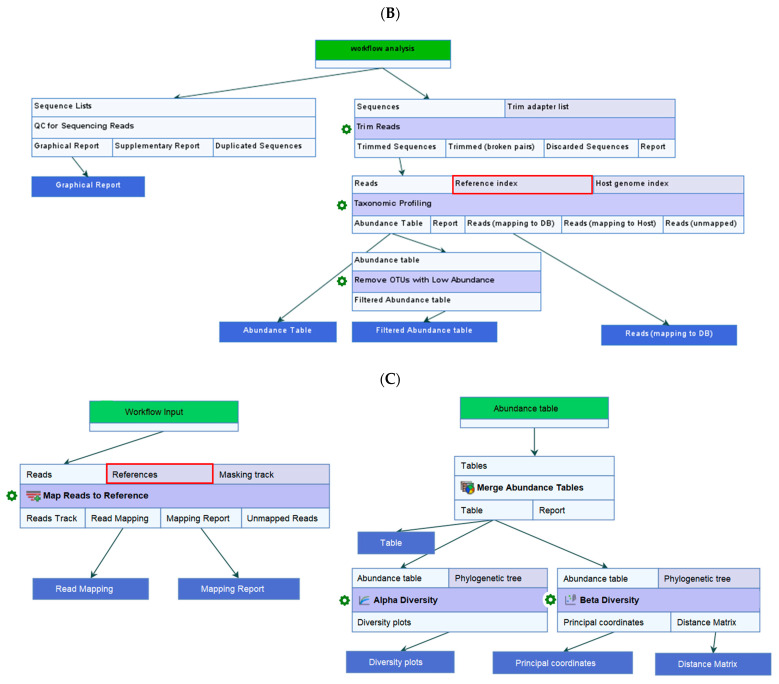
CLC Workflows, Tools, and Databases. (**A**) The Microbial Genomics Module offers workflows (blue arrow) and tools (black arrow) for taxonomic and diversity analysis (**left**), alongside the databases and dataset used in this study (**right**). (**B**) The Data QC and Taxonomic Profiling workflow uses the customized VAGIBIOTA or UNITE Reference Index (red outline) to generate QC reports and Abundance Tables from NGS reads obtained from clinical samples. These tables feed into the diversity analysis workflow. (**C**) Reads identified as “*Lactobacillus* spp. unknown” are further analyzed using the Map Reads to Reference workflow which incorporates the VAGIBIOTA Sequence List for species identification (**left**). The Merge and Estimate Alpha and Beta Diversities workflow produces diversity plots and statistics (**right**).

The SILVA database organizes taxonomic information into seven ranks [94]. For instance, *L. crispatus* is classified as follows: Domain: Bacteria; Phylum: Firmicutes; Class: Bacilli; Order: Lactobacillales; Family: Lactobacillaceae; Genus: Lactobacillus; Species: Crispatus. Additional genomes obtained from NCBI were annotated using the same taxonomy. The annotated genome sequences were then compiled into three distinct CLC database formats: (1) Vaginal Microbiome Taxonomic Profiling Index, (2) Vaginal Microbiome Sequence List, and (3) Vaginal Microbiome BLAST database (Figure 9A) [41]. The CLC Microbial Genomics Module, version 25.0.1 tools used for this process included Create Taxonomic Profiling Index, Sequence List, and Create BLAST Database [41]. These three reference databases were subsequently integrated and applied in specific workflows and tools, namely: (1) Taxonomic Profiling, (2) Map Reads to Reference, and (3) multi-BLAST tool (version 2.17.0+).

### 4.3. Data Quality Control (QC), Taxonomic Profiling, and Read Mapping

For this project, CLC Genomics Workbench versions 22.0.2 to 25.0.1 [40] and CLC Microbial Genomics Module (MGM) versions 22.1 to 25.0.1 [41] (Redwood City, CA, USA) were installed on an HP notebook equipped with an Intel i7-7500U dual-core processor running at 2.70 GHz and 8 GB of RAM. The MGM provides a range of pre-built workflows and tools, as depicted in Figure 9A. Initially, the QIAseq 16S/ITS demultiplexer tool was employed to segregate NGS reads based on the barcodes of bacterial 16S rRNA variable regions (V1V2, V2V3, V3V4, V4V5, V5V7, V7V9) and fungal ITS regions, as obtained using the QIAGEN QIAseq 16S/ITS screening panel. The tool generated sequence lists for each region, which were subsequently fed into the Data QC and Taxonomic Profiling workflow. Taxonomic Profiling then aligned each input read to a reference sequence, accompanied by qualification and quantification data to create an abundance table. The abundance table was utilized in abundance analysis workflows or tools for visualization and statistical analyses [41].

The pre-built “Data QC and Taxonomic Profiling” workflow, including its input/output data, processing steps, and directional flow (arrows), is depicted in Figure 9B. The analysis involved four main steps: Data Import, Data QC, Taxonomic Profiling, and Visualization of the Abundance Table. The process began with the import of FASTQ files generated from sequencing as paired-end (forward and reverse) reads, which were merged using the “Import Illumina” tool and placed into a newly created “input” folder. Next, the workflow was launched by selecting “Run,” initiating the following tasks: (1) selecting input files, (2) preprocessing reads through quality trimming (with a quality score cutoff of 0.05, ambiguity limit ≤ 2, and adapter trimming), (3) mapping and assigning 16S or ITS reads to the respective VAGIBIOTA or UNITE reference index, and (4) quantifying and generating an abundance table for each sample after filtering out low-abundance Operational Taxonomic Units (OTUs), defined as those with fewer than 50 reads or representing less than 1.0% of total reads.

Reads classified as “*Lactobacillus* species unknown” by taxonomic profiling were extracted and mapped to the VAGIBIOTA reference sequence list using the “Map Reads to Reference” workflow (Figure 9C). The mapping process involved three key steps: importing paired-end reads, aligning reads to reference genomes, and generating a read mapping output file. The resulting table displayed the identified microbial species along with their corresponding read counts. To validate the mapping results, the “Extract Consensus” tool was employed to retrieve the consensus sequence from the reads for BLAST analysis [41].

Following taxonomic profiling and speciating *Lactobacillus* spp. unknown reads, the vaginal microbial composition of each sample was classified into the five community state types (CST I-V) as defined by Ravel et al. [11]. CST I, II, III, and V are dominated by *Lactobacillus* spp., accounting for more than 50% of the total species. Specifically, CST I-III and V are dominated by *L. crispatus*, *L. gasseri*, *L. iners*, and *L. jensenii*, respectively. In contrast, CST IV is predominantly composed of Gram-negative, non-*Lactobacillus* species, with *Lactobacillus* spp. making up less than 50%. Gajer et al. further divided CST IV into subcategories IV-A and IV-B, with IV-A characterized by “modest proportions” of *Lactobacillus* spp. and low levels of anaerobic bacteria [13]. To quantify the proportions of *Lactobacillus* spp. among the CSTs, cluster analysis was performed to broadly group all samples into three *Lactobacillus* states: *L.*-dominant (CST I-III and V), *L.*-diminished (IV-A), and *L.*-depleted (CST IV-B). Partition clustering with k-means (k = 3) and Euclidean distance was employed to group samples based on the abundance levels (%) of *Lactobacillus* spp. The mean and 95% confidence intervals were then calculated to determine the abundance levels (%) of *Lactobacillus* spp. for each cluster, distinguishing between CST.

The final steps of taxonomic profiling were as follows. First, the taxonomic tables from all samples were combined into a single table using the “Merge Abundance Tables” tool. Next, clinical metadata, including CST classification in .xlsx format, was integrated into the merged taxon table with the “Add Metadata to Abundance Table” tool. Finally, the merged abundance table was grouped by features such as “CST,” “HPV,” and “Pap” using the “Aggregate feature” option for grouped analysis.

### 4.4. Estimate Alpha and Beta Diversities Workflow

The pre-built “Merge and Estimate Alpha and Beta Diversities” workflow (Figure 9C) was employed to analyze and compare vaginal microbial communities within samples and between groups. The analysis included three main steps: importing and merging abundance tables, conducting alpha and beta diversity analyses, and visualizing diversity plots. The workflow began by selecting “Run” to execute the following tasks: (1) selecting cleaned microbial abundance tables with appended metadata, (2) merging the abundance tables, (3) calculating α-diversity to evaluate within-sample variation, and (4) calculating β-diversity to assess variation between samples. For this study, two α-diversity measures were computed, i.e., Simpson’s index [41]: SI = 1 $-\sum_{i=1}^{n}p_{i}^{2}$, and Shannon entropy [41]: H = $\sum_{1}^{n}p_{i}$log_2_*p_i_*, where *n* was the number of microbial species found in the sample, and *p_i_* was the proportion of reads that were identified as the *ith* microbial species. The workflow produced rarefaction curves, boxplots, and automated Mann–Whitney U statistical results, which compared alpha diversity indices across groups.

β-diversity, reflecting differences in microbial composition (proportion) between groups, was assessed using Bray–Curtis distances which is a metric of dissimilarity based on species abundance. The resulting distance matrix quantified inter-sample variation. Principal Coordinate Analysis (PCoA) was used to visualize these differences in 3D space. Statistical significance between group centroids and dispersion was evaluated using the permutational multivariate analysis of variance (PERMANOVA) tool.

### 4.5. Differential Abundance and Heatmap Analysis of Cervicovaginal Microbiota

The “Differential Abundance Analysis” tool was employed to analyze vaginal microbial abundance across CST groups using CST I as the reference. The merged abundance table served as input for pairwise comparisons, generating fold changes, *p*-values, and adjusted *p*-values (FDR or Bonferroni) via the Wald test [41]. A Venn diagram was auto-generated to visualize overlaps in the pairwise comparisons. The “Create Heat Map for Abundance Table” tool was employed to generate a hierarchically clustered heat map, illustrating variations in microbial species composition across individual or grouped samples. Parameters were set with 1-Pearson correlation for distance and average linkage for clustering [41].

### 4.6. Statistical Analysis

Data were summarized using means with 95% CI, medians with interquartile ranges (IQRs), and proportions. Non-parametric hypothesis tests, namely, Wilcoxon rank-sum, Kruskal–Wallis, and PERMANOVA were applied to numerical, ordinal, and multivariate data, respectively. The Wald test assessed differential abundance, and chi-square was used for categorical comparisons. Significance was set at *p* < 0.05. Analyses were performed using the built-in functions of CLC tools and STATA/IC 17.0 (StataCorp LLC, College Station, TX, USA).

## 5. Conclusions

Our findings indicate that microbial phylogenetic composition varies markedly with the choice of 16S hypervariable region. Consequently, employing a comprehensive V1–V9 screening panel is essential for identifying the most suitable region for accurate community profiling. By systematically identifying sources of methodological bias, we established a practical framework for reliable microbiome research. These insights not only address current challenges but also open new avenues for validating and exploring HPV-associated microbiomes across diverse anatomical econiches. Continued advancements in sequencing technologies will be essential for overcoming methodological limitations and improving analytical precision. Ultimately, this work will contribute meaningfully to advancing our understanding of carcinogenesis driven by HPV and microbe-associated inflammation.

## Data Availability

The dataset (demultiplexed V1V2 and V3V4 FASTQ files) presented in this study are openly available in the NCBI Sequence Read Archive (SRA). Title: Cervicovaginal microbiome and HPV: A standardized workflow from 16S/ITS NGS to microbial community profiling. BioProject Accession Number: PRJNA1270007; BioSample Accession Numbers: SAMN48809336 to 48809403.

## Supplementary Materials

The supporting information can be downloaded at: https://www.mdpi.com/article/10.3390/ijms26168090/s1.

## Abbreviations

ASCUS, Atypical Squamous Cells of Undetermined Significance; BV, bacterial vaginitis or vaginosis; CIN, Cervical Intraepithelial Neoplasia; CVM, cervicovaginal microbiota; CST, Community State Types; GBS, Group B Streptococcus; HSIL, High-grade Squamous Intraepithelial Lesion; IQR, interquartile range; LSIL, Low-grade Squamous Intraepithelial Lesion; MGM, CLC Microbial Genomics Module; NGS, next-generation sequencing; NILM, Negative for Intraepithelial Lesion or Malignancy; PCoA, principal coordinate analysis; PID, Pelvic Inflammatory Disease; QC, Quality Control; UTI, urinary tract infection.
