# Supplementary material for: Cervicovaginal Microbiome and HPV: A Standardized Approach to 16S/ITS NGS and Microbial Community Profiling for Viral Association
Source: Int J Mol Sci. 2025 Aug 21;26(16):8090. doi: 10.3390/ijms26168090 (PMC12386612; doi:10.3390/ijms26168090)
Supplement: Supplementary file 1 [file ijms-26-08090-s001.zip › ijms-3725007_SUPP_R1_/FIG S1_TAXON PROFILES 16S V1-V9, N-66.pdf]

A

Sample ID:
30856-001
NILM
HPV-NEG

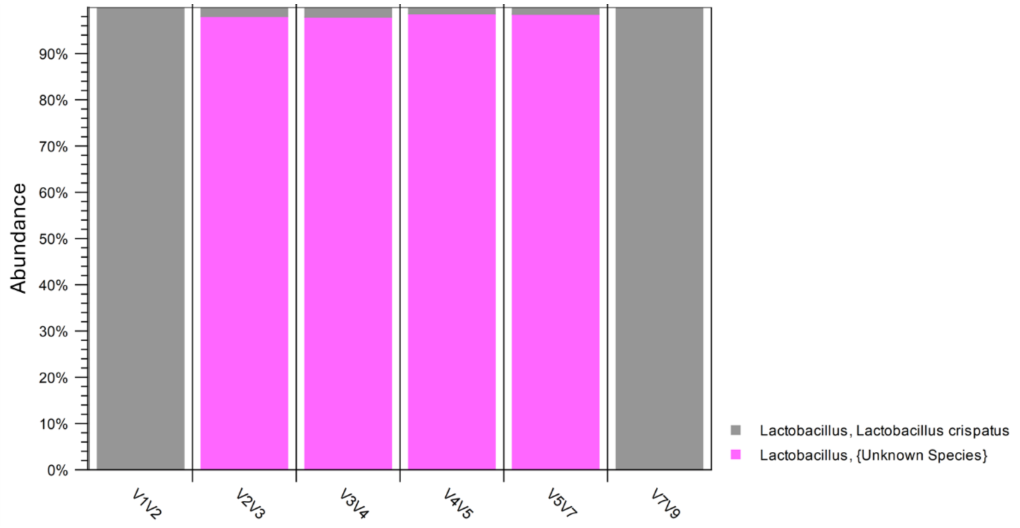

B

Sample ID:
30856-002
NILM
HPV-NEG

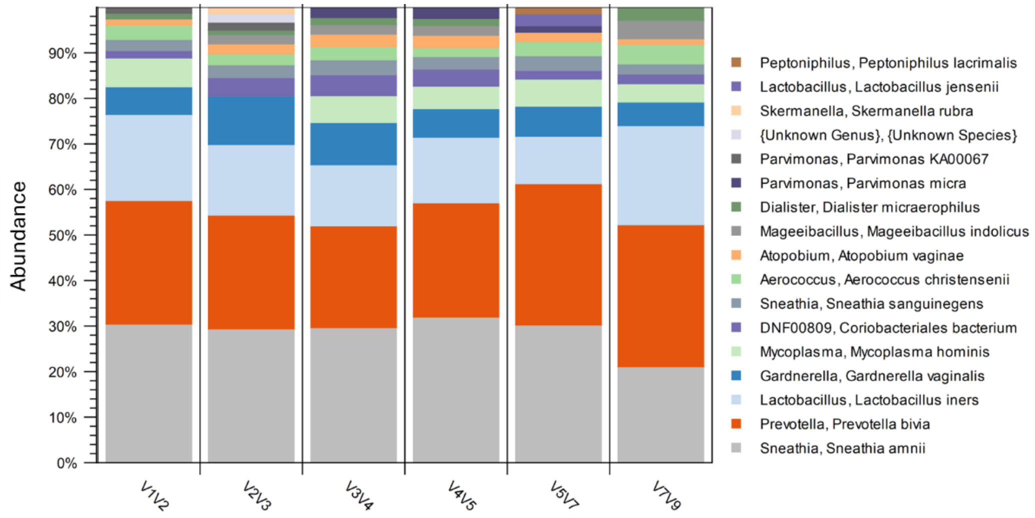

C

Sample ID:
30856-003
NILM
HPV-NEG

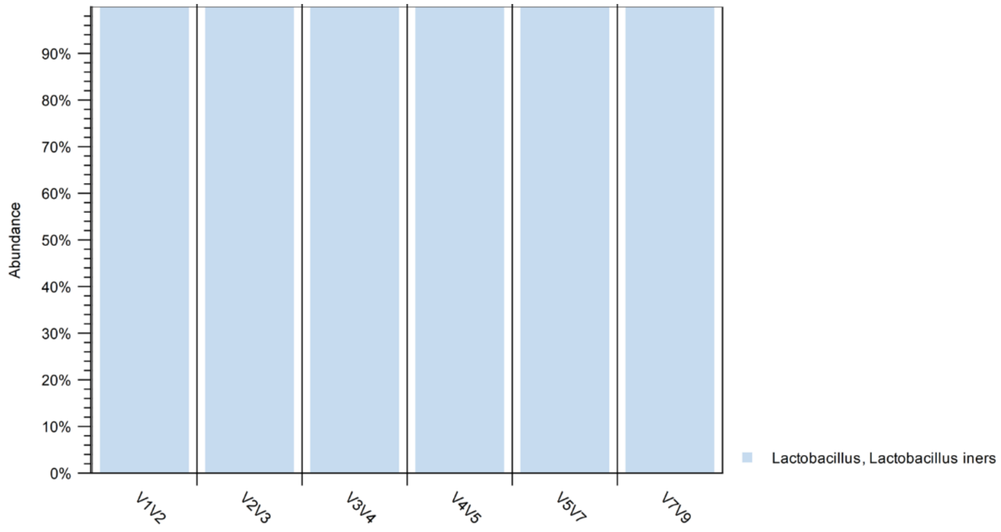

D

Sample ID:
30856-004
NILM
HPV-NEG

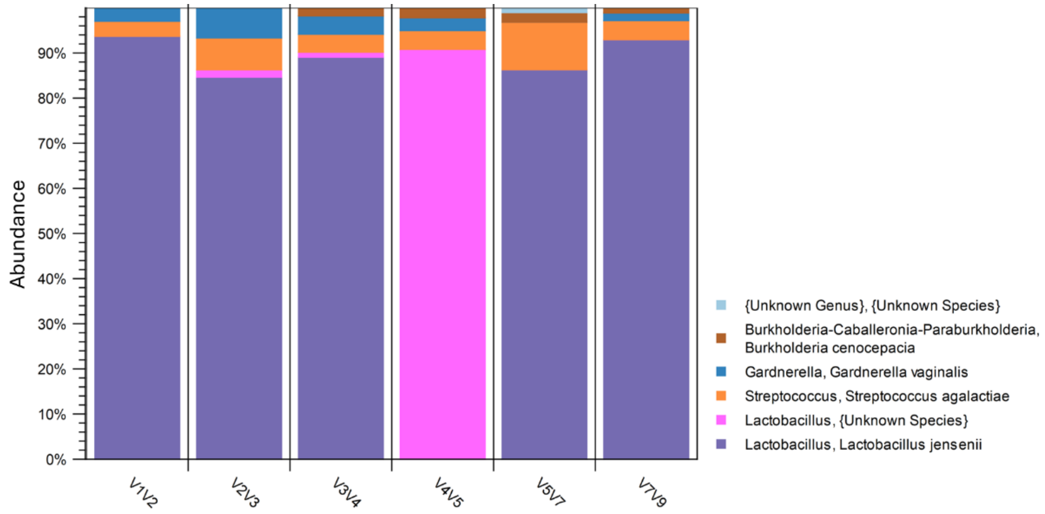

E

Sample ID:
30856-005
NILM
HPV-NEG

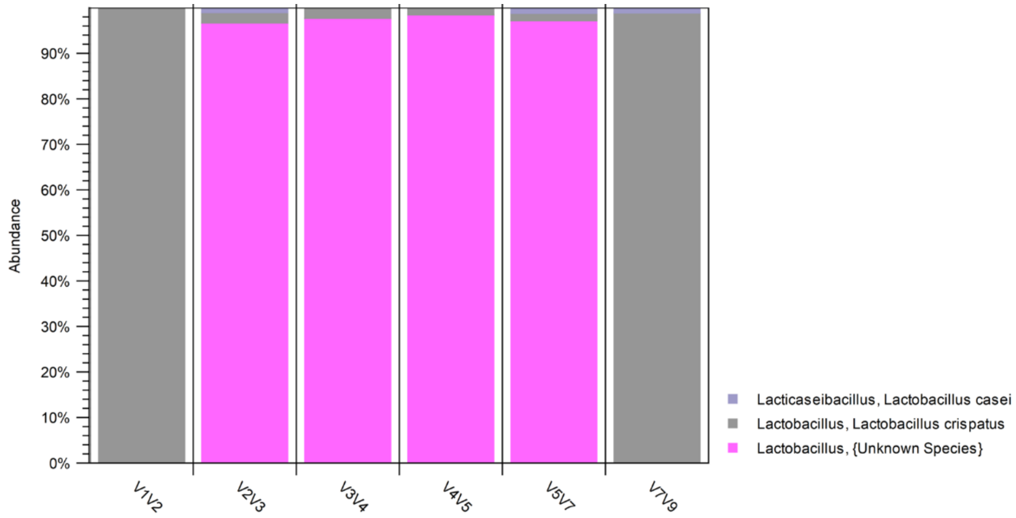

F

Sample ID:
30856-006
NILM
HPV-NEG

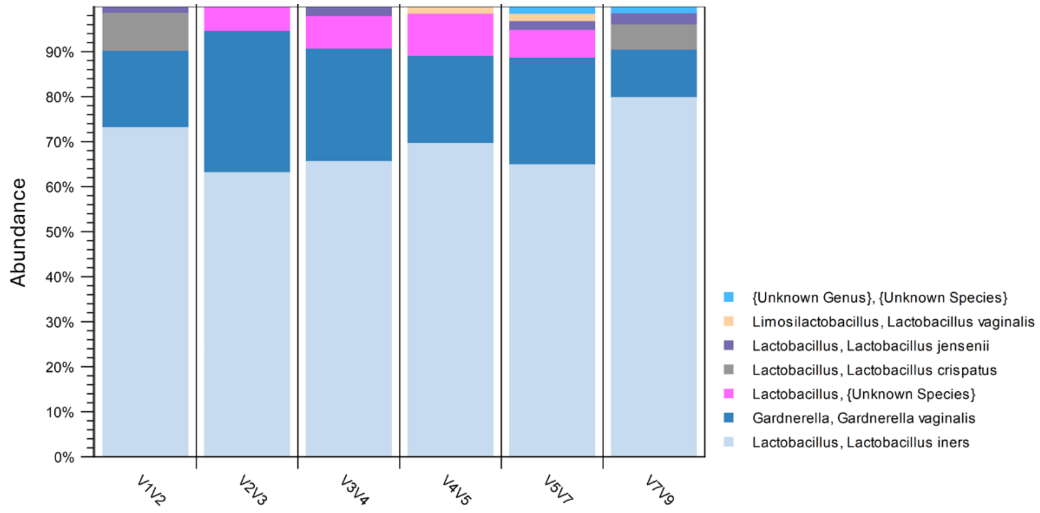

G

Sample ID:
30856-007
NILM
HPV-NEG

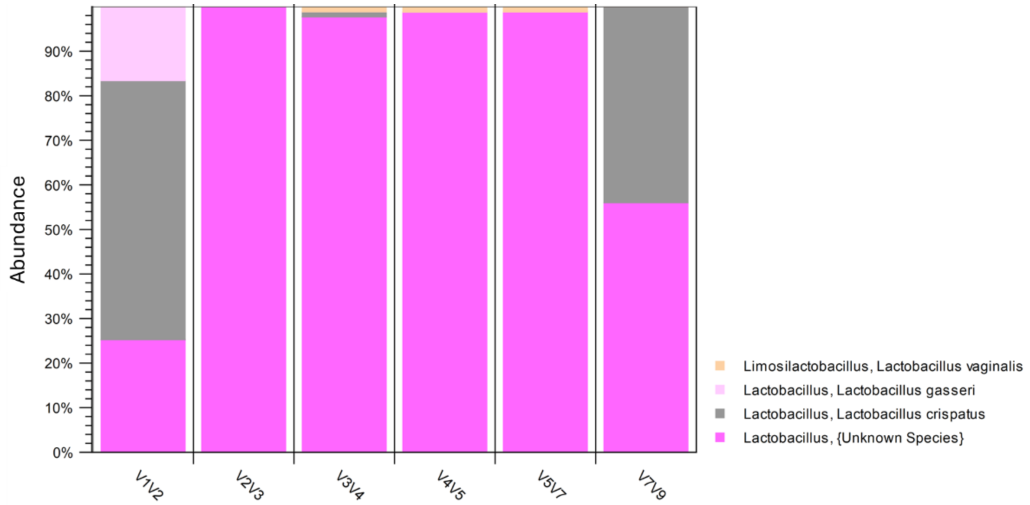

H

Sample ID:
30856-008
NILM
HPV-NEG

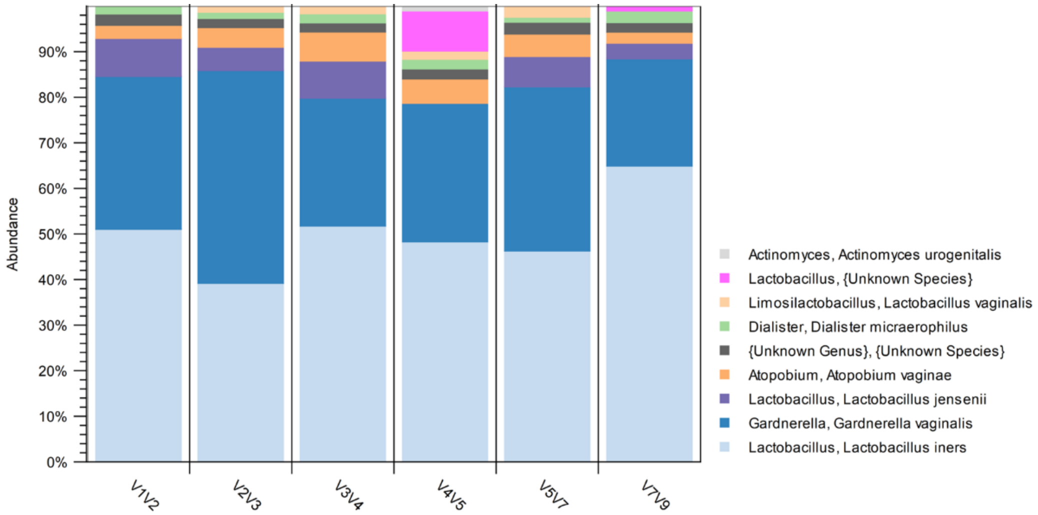

I

Sample ID:
30856-009
NILM
HPV-NEG

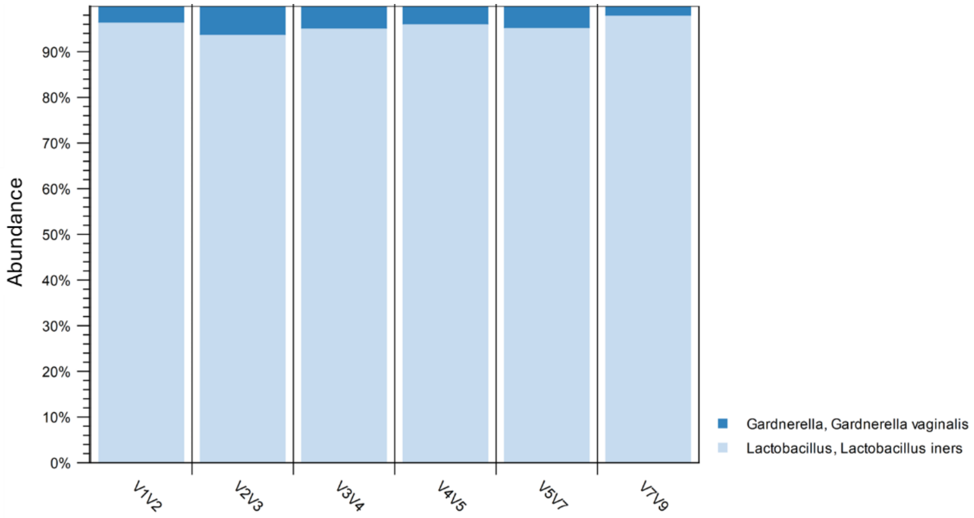

J

Sample ID:
30856-010
NILM
HPV-NEG

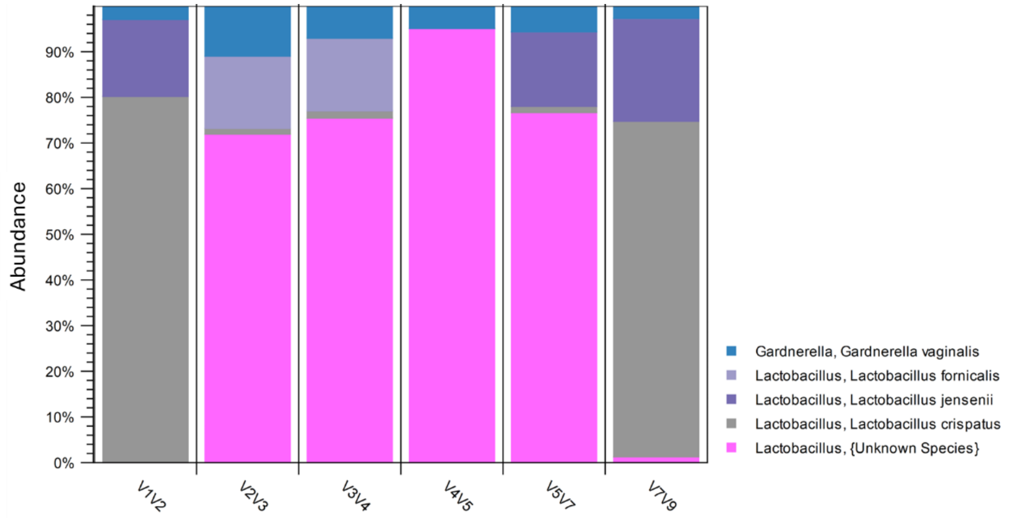

K

Sample ID:
30856-011
NILM
HPV-NEG

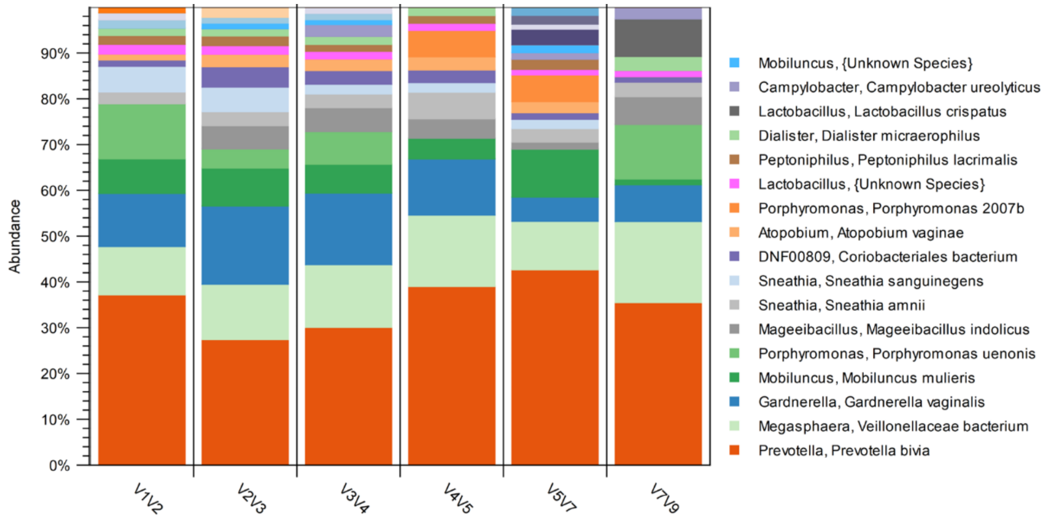

L

Sample ID:
30856-012
NILM
HPV-NEG

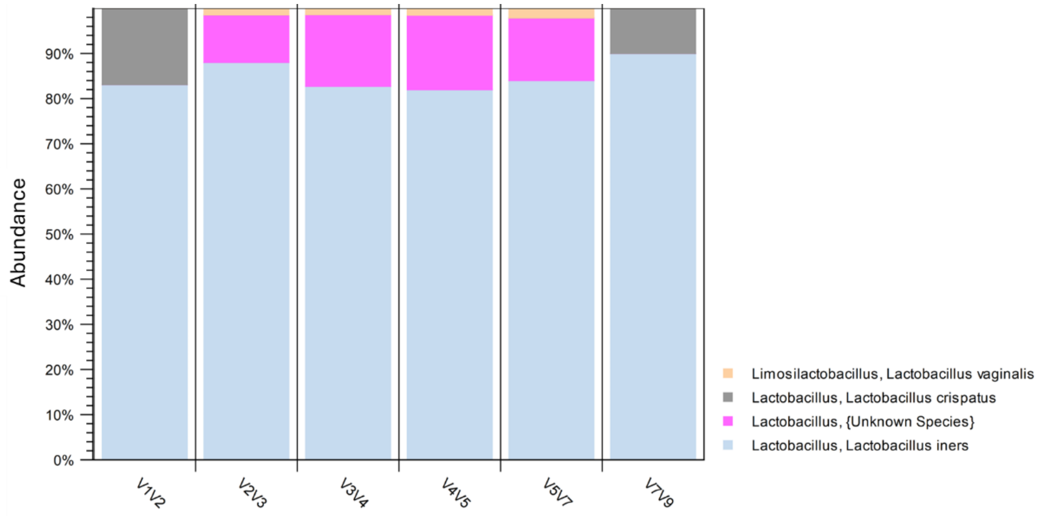

M

Sample ID: 30856-013 ASCUS HPV-NEG

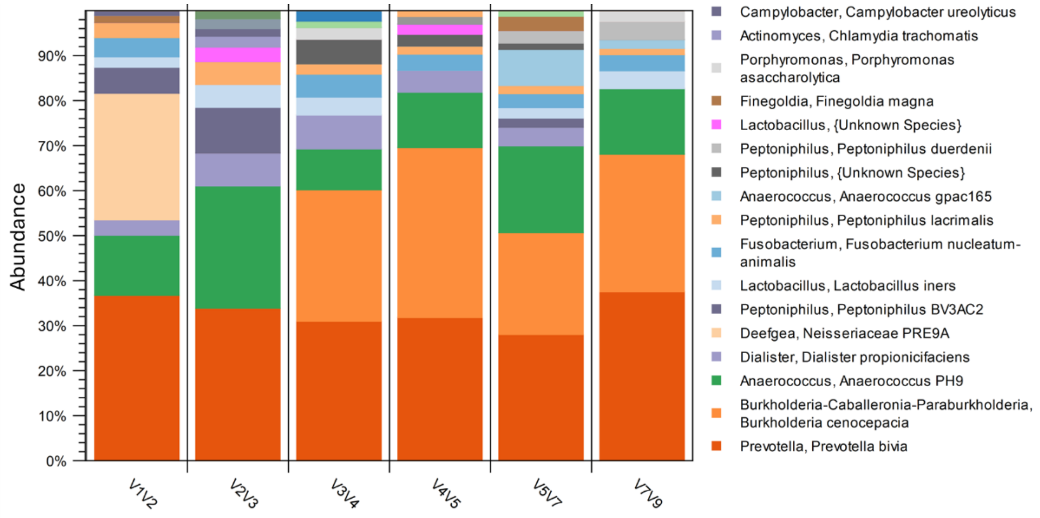

N

Sample ID: 30856-014 ASCUS HPV-NEG

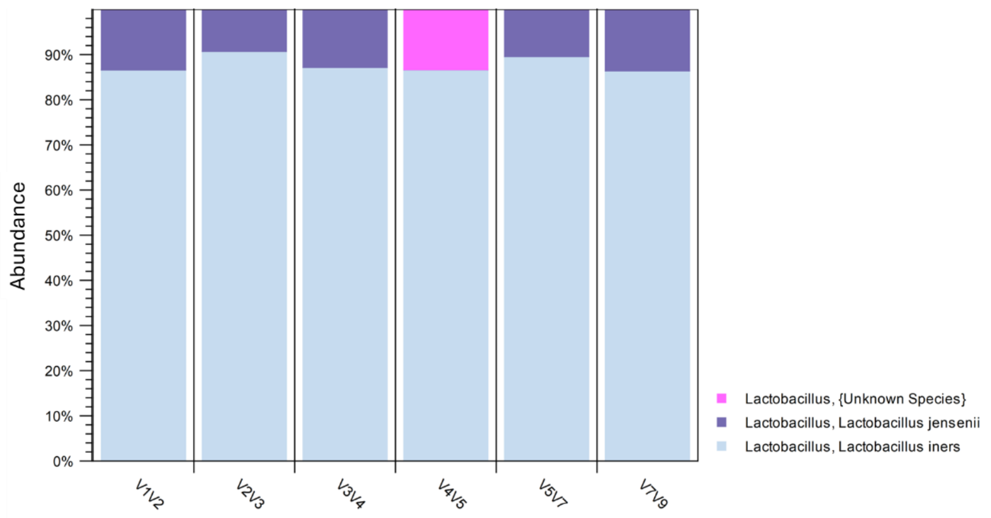

O

Sample ID: 30856-015 ASCUS HPV-NEG

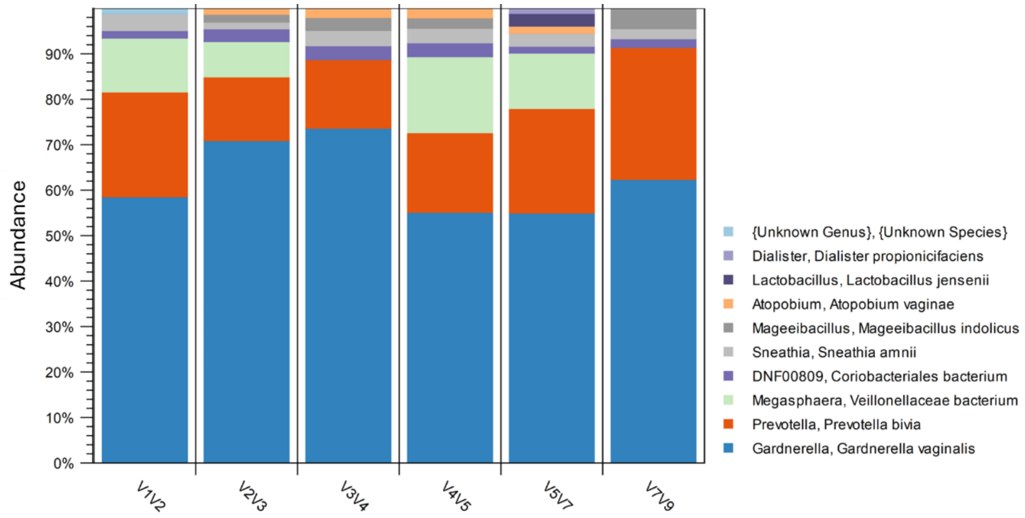

P

Sample ID:
30856-016
ASCUS
HPV-NEG

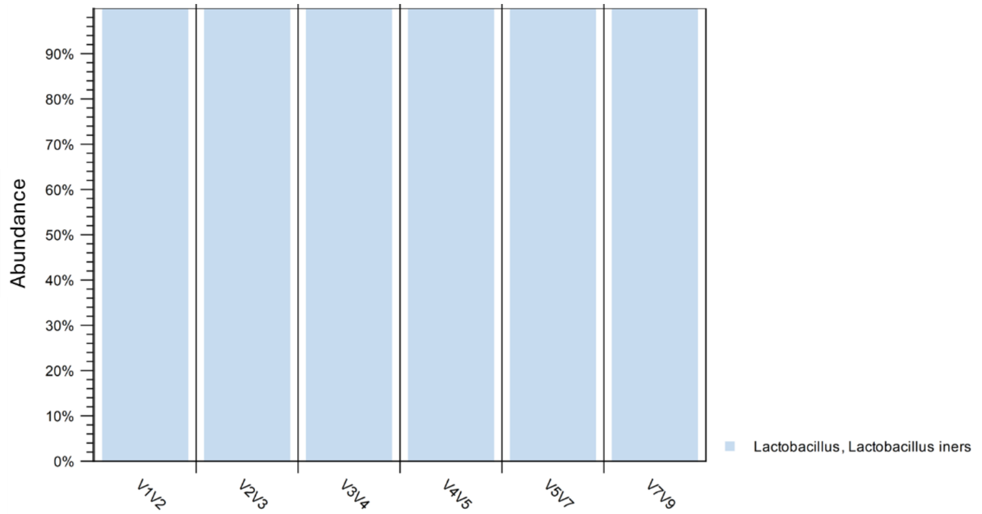

Q

Sample ID:
30856-017
ASCUS
HPV-NEG

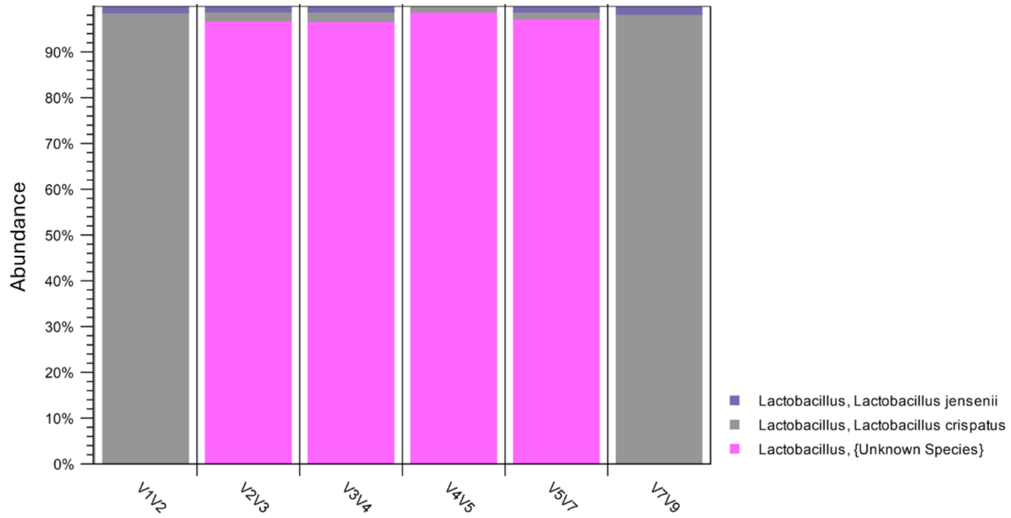

R

Sample ID:
30856-018
ASCUS
HPV-NEG

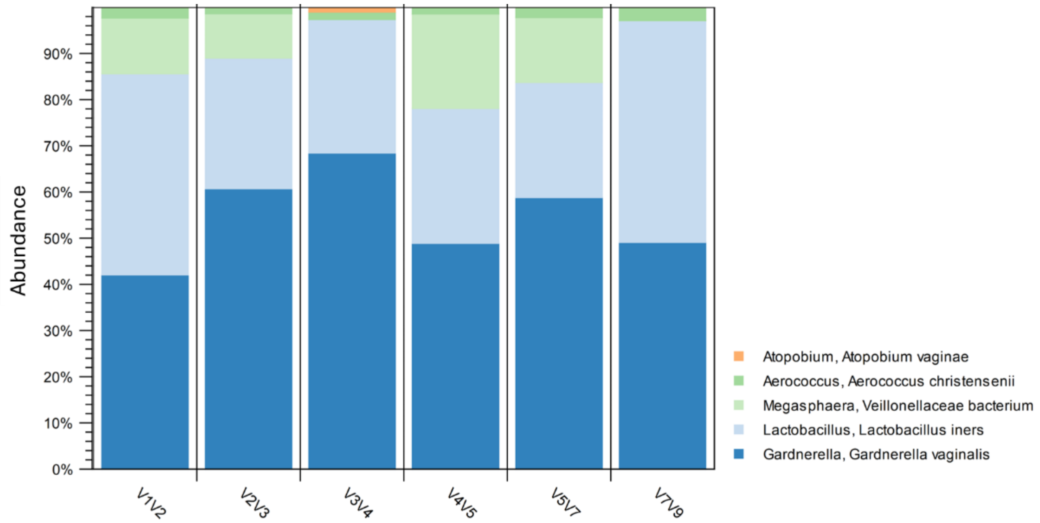

S

Sample ID:
30856-019
ASCUS
HPV-NEG

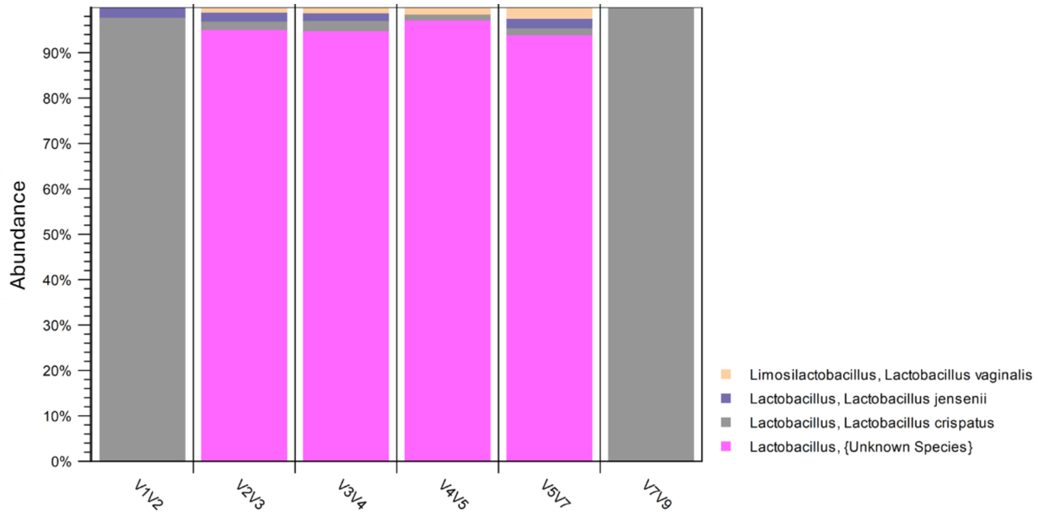

T

Sample ID:
30856-020
ASCUS
HPV-NEG

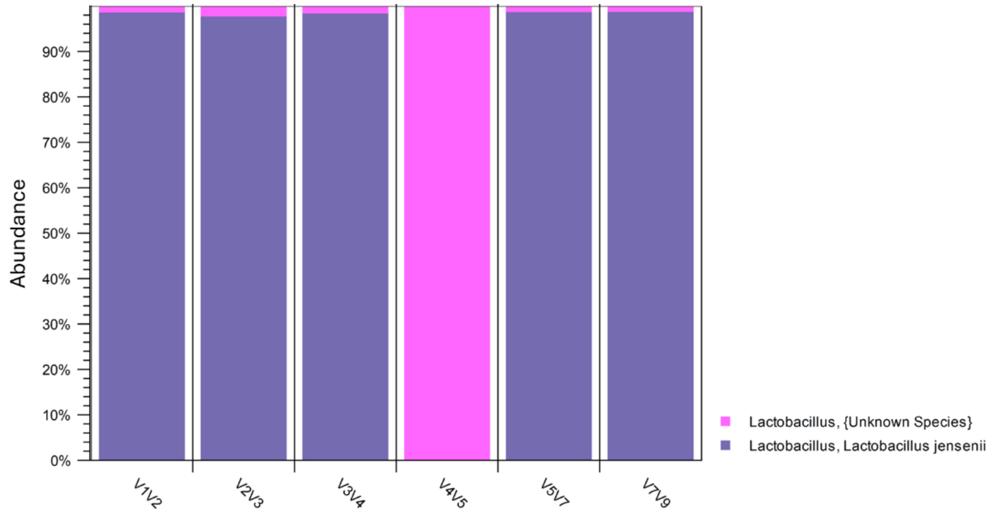

U

Sample ID:
30856-021
ASCUS
HPV-NEG

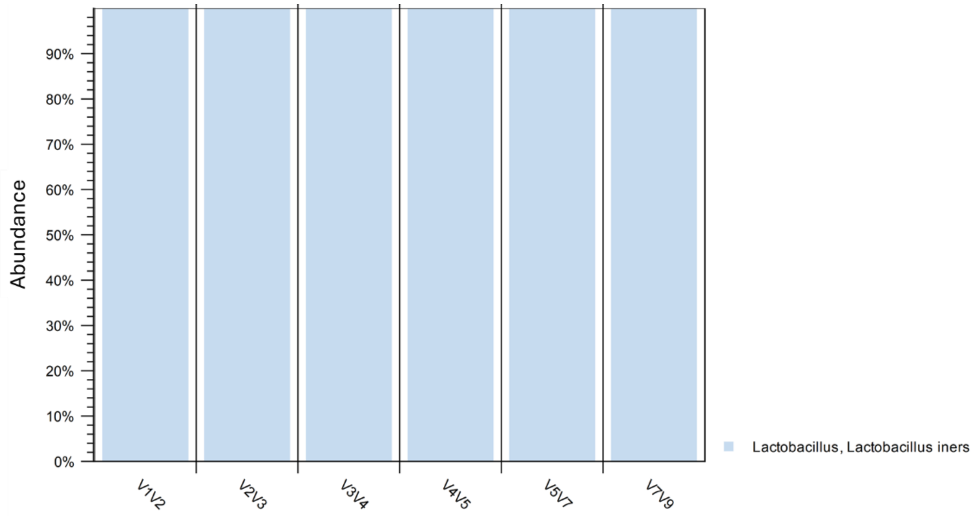

V

Sample ID:
30856-022
ASCUS
HPV-NEG

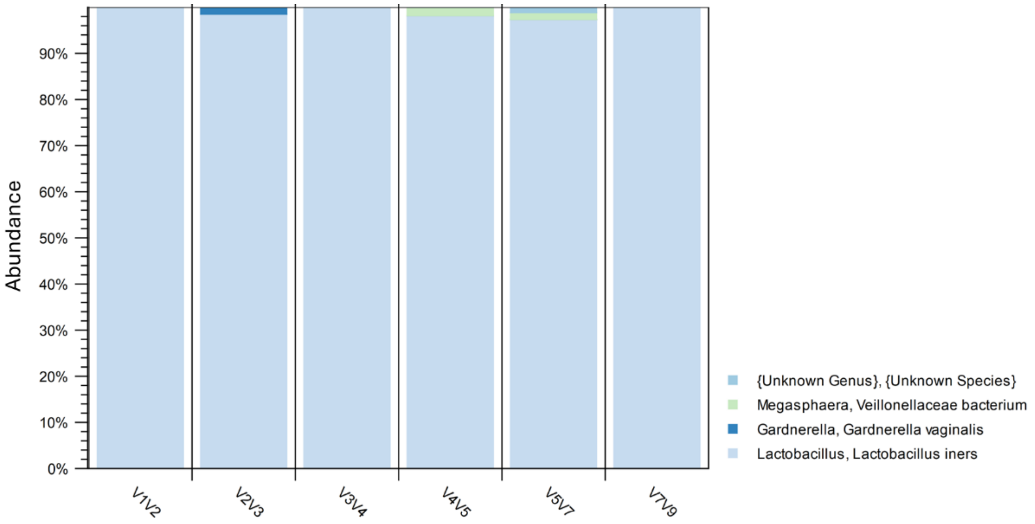

W

Sample ID:
30856-023
ATCC STD

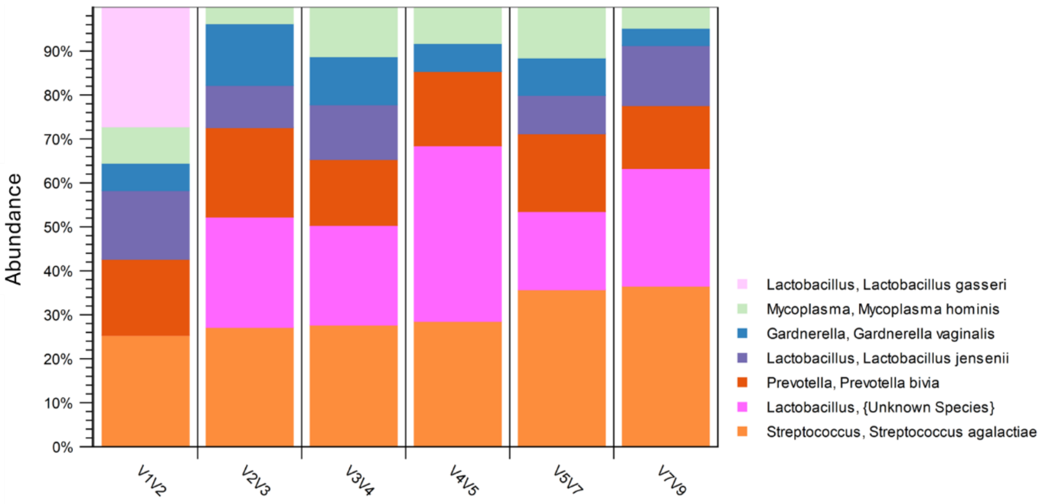

X

Sample ID:
30856-024
ATCC STD

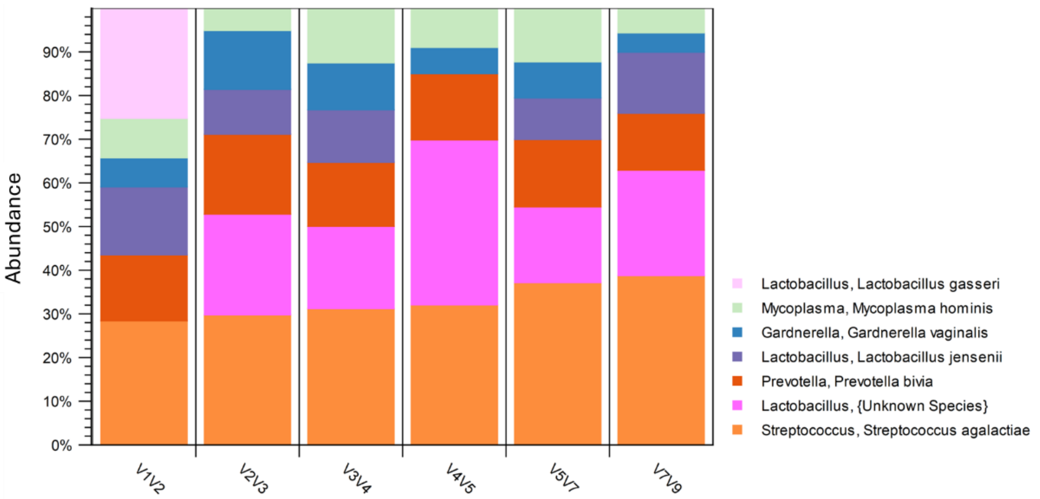

Y

Sample ID:
30663-001
LSIL
HPV-POS

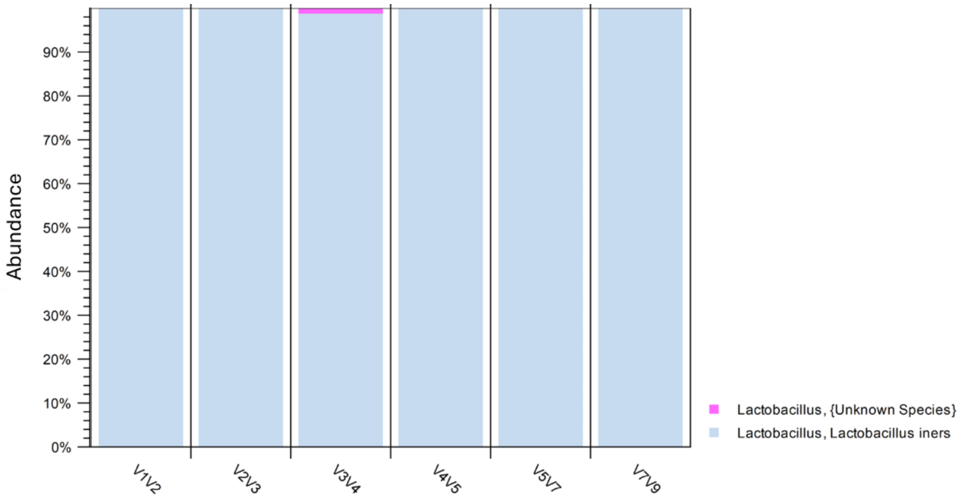

Z

Sample ID:
30663-002
LSIL
HPV-POS

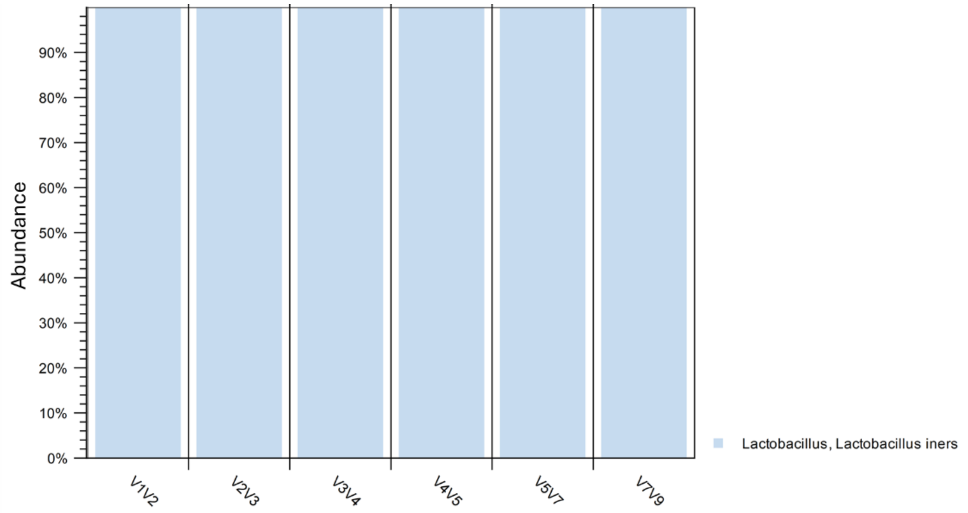

AA

Sample ID:
30663-003
LSIL
HPV-POS

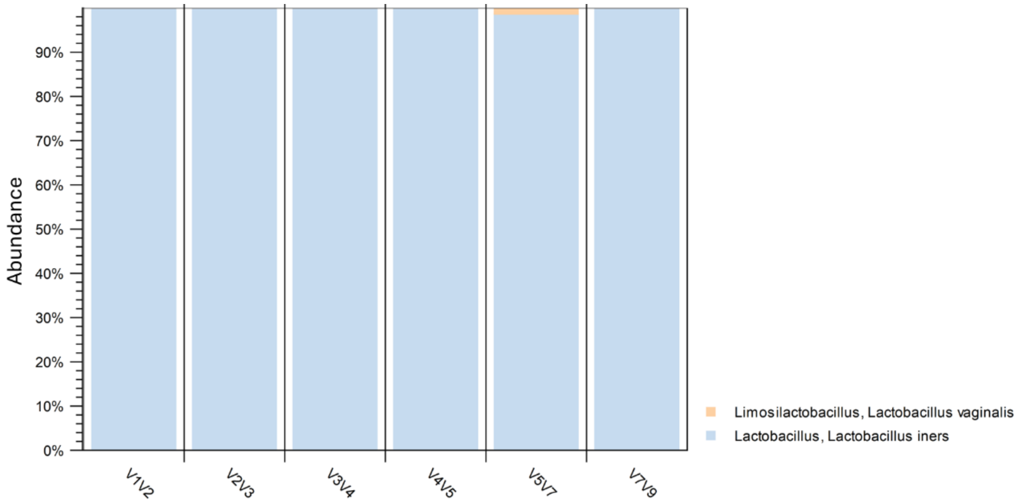

AB

Sample ID:
30663-004
LSIL
HPV-POS

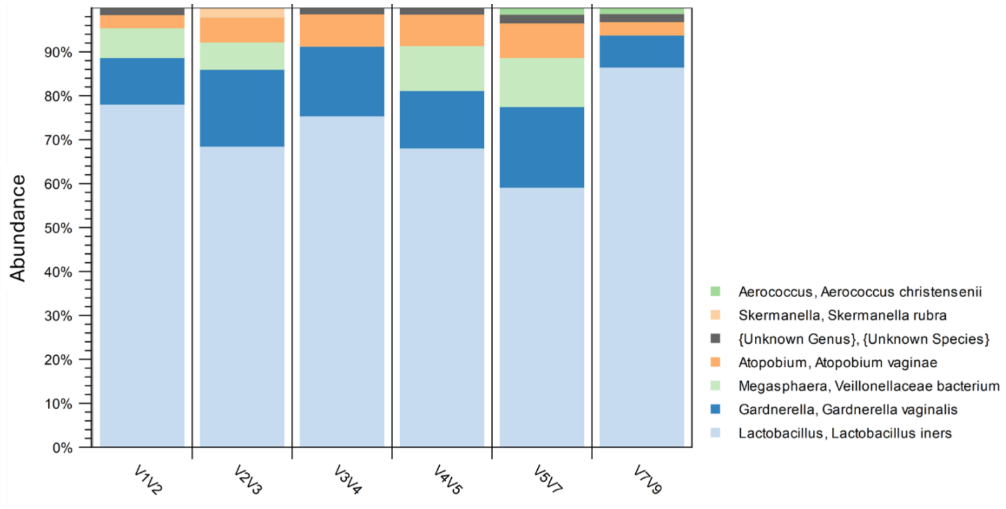

AC

Sample ID:
30663-005
LSIL
HPV-POS

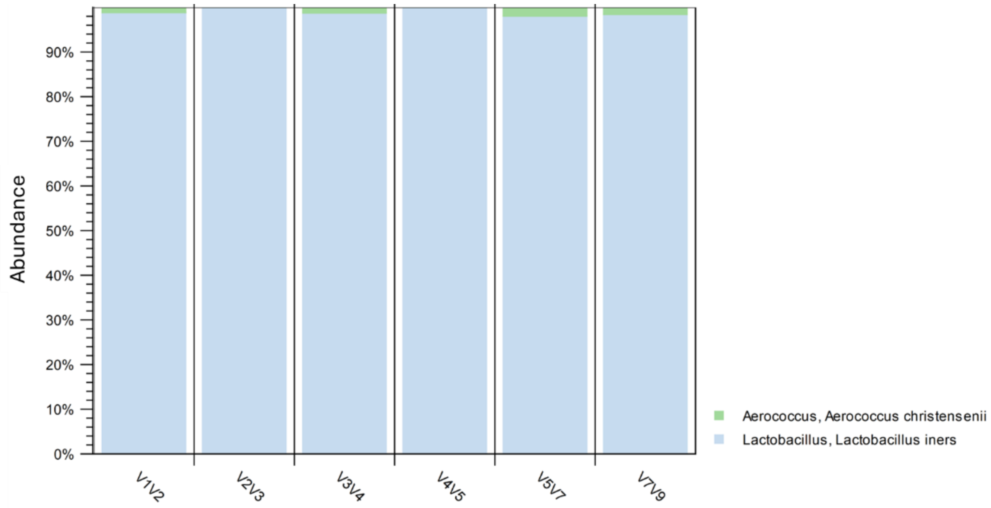

AD

Sample ID:
30663-006
LSIL
HPV-POS

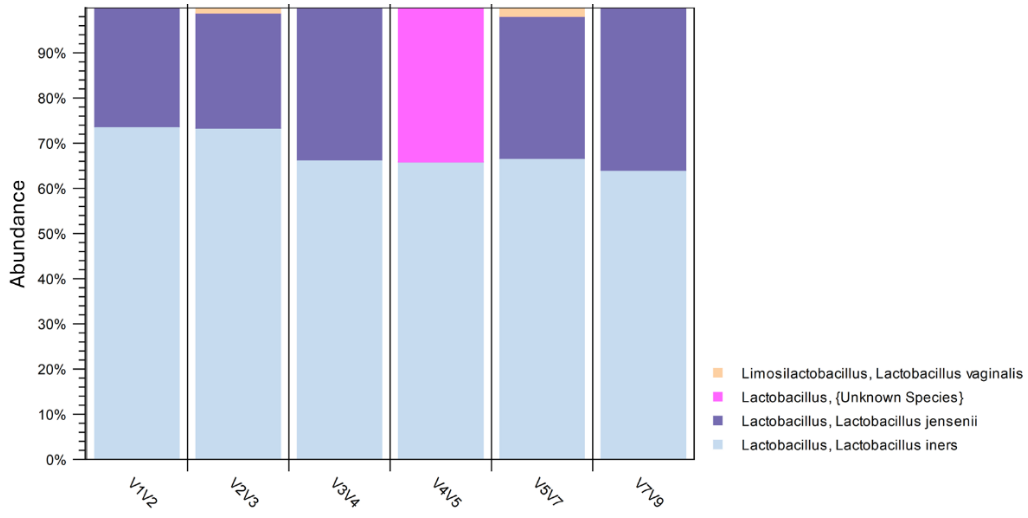

AE

Sample ID:
30663-007
LSIL
HPV-POS

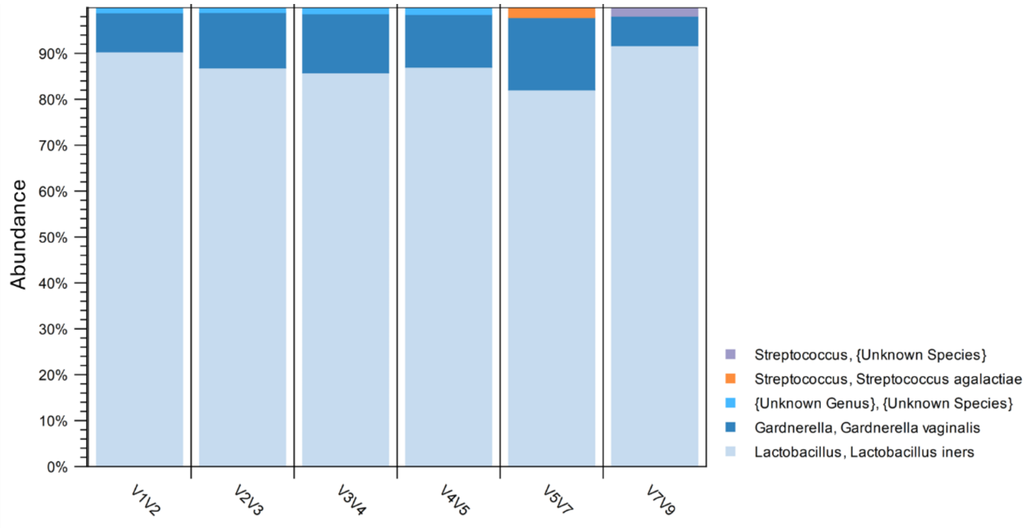

AF

Sample ID:
30663-008
LSIL
HPV-POS

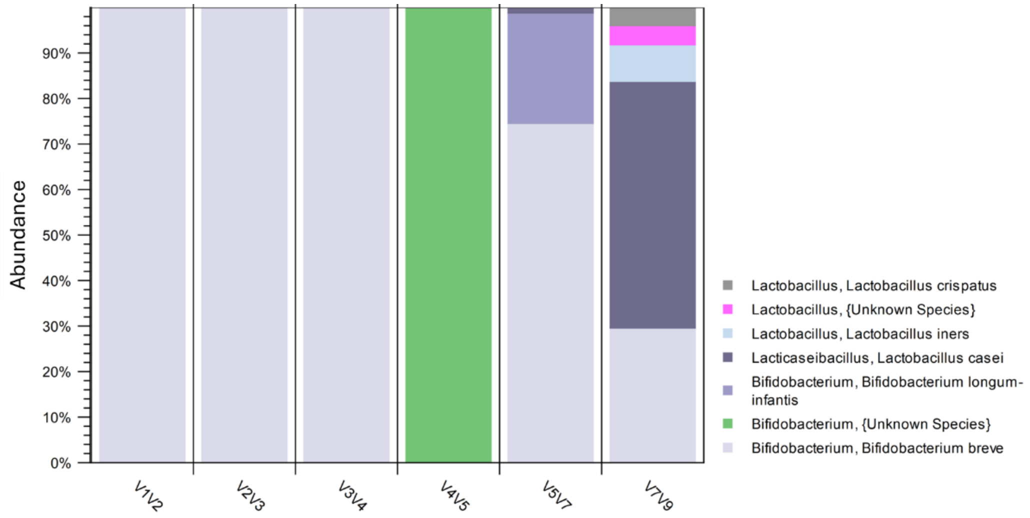

AG

Sample ID:
30663-009
LSIL
HPV-POS

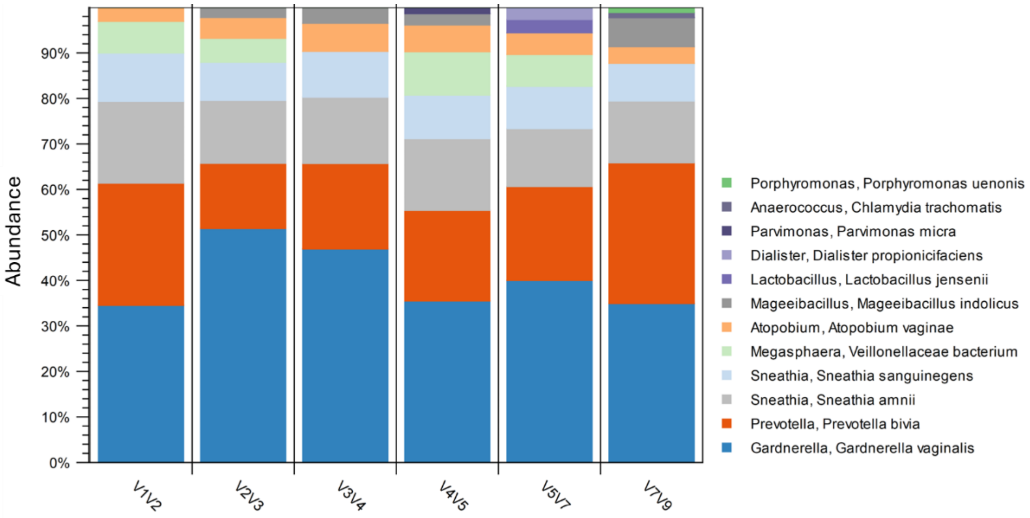

AH

Sample ID:
30663-010
LSIL
HPV-POS

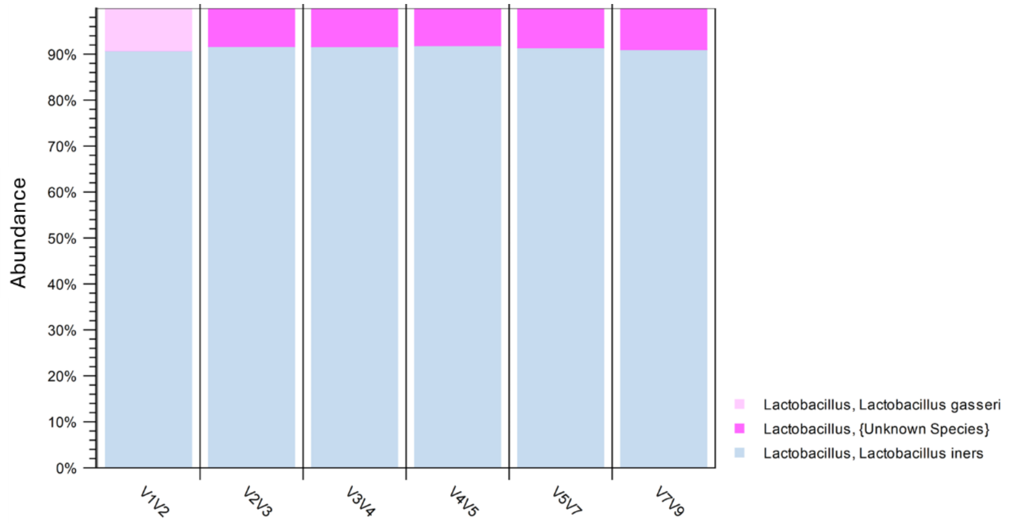

AI

Sample ID:
30663-011
LSIL
HPV-POS

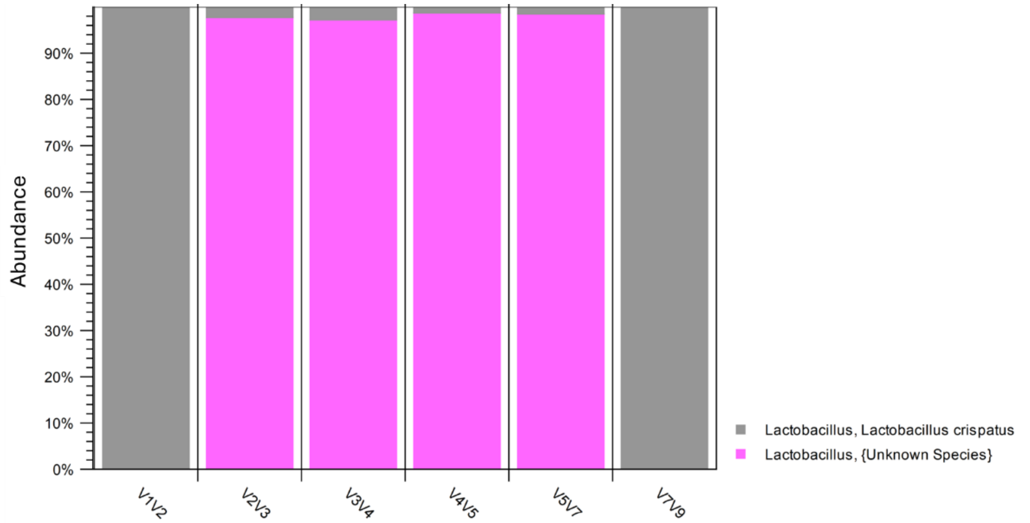

AJ

Sample ID:
30663-012
LSIL
HPV-POS

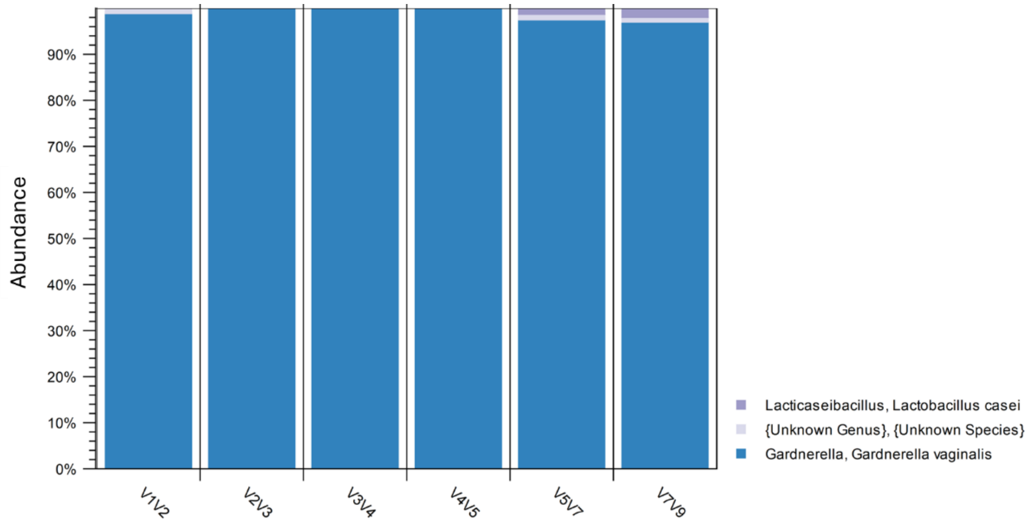

AK

Sample ID:
30663-013
LSIL
HPV-POS

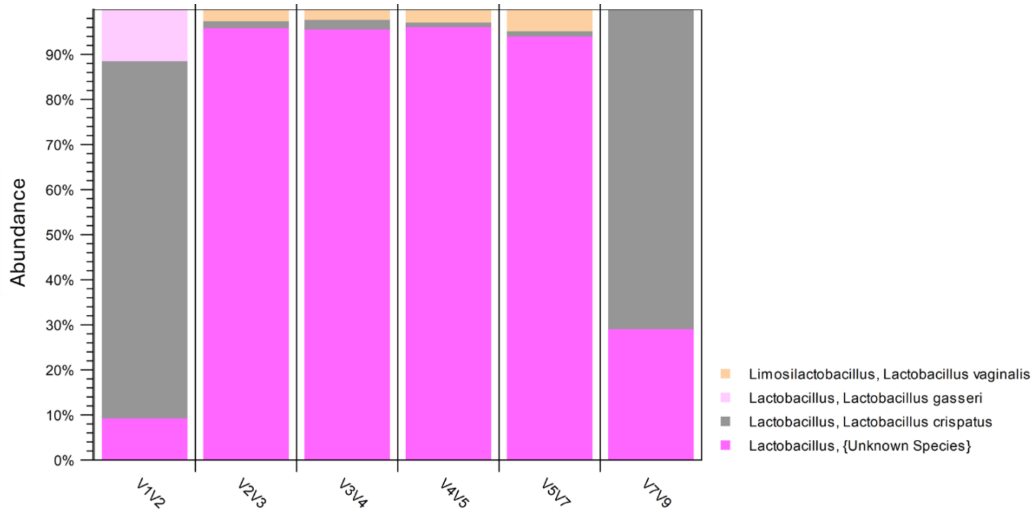

AL

Sample ID:
30663-014
LSIL
HPV-POS

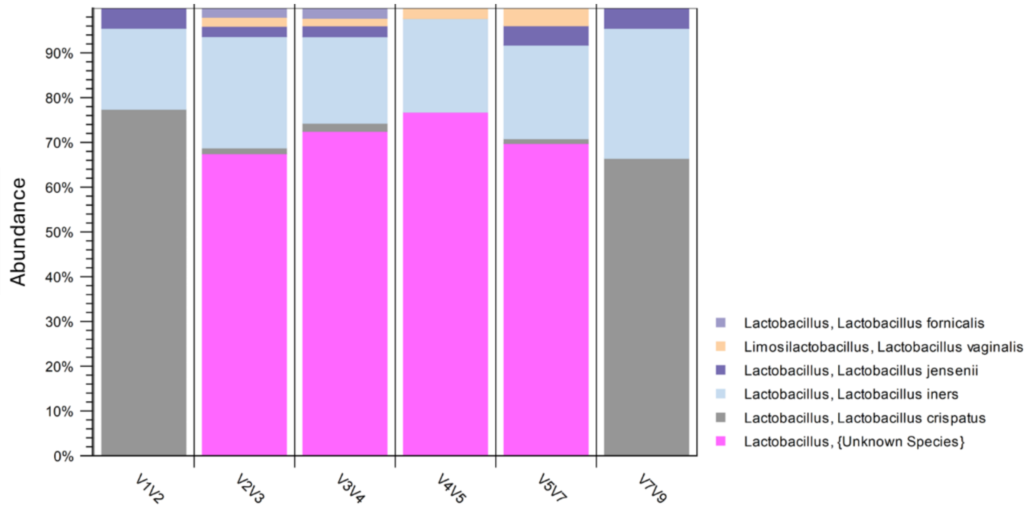

AM

Sample ID:
30663-015
LSIL
HPV-POS

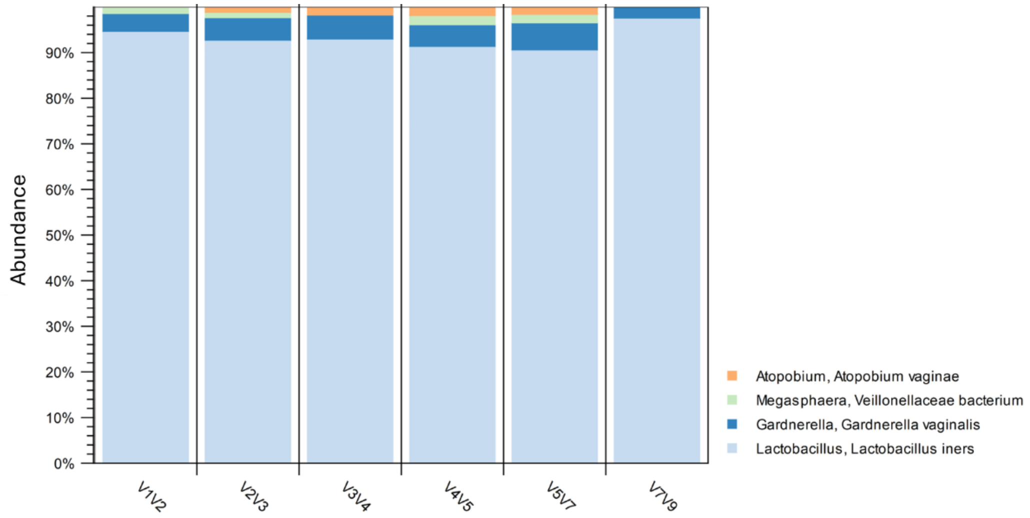

AN

Sample ID:
30663-016
LSIL
HPV-POS

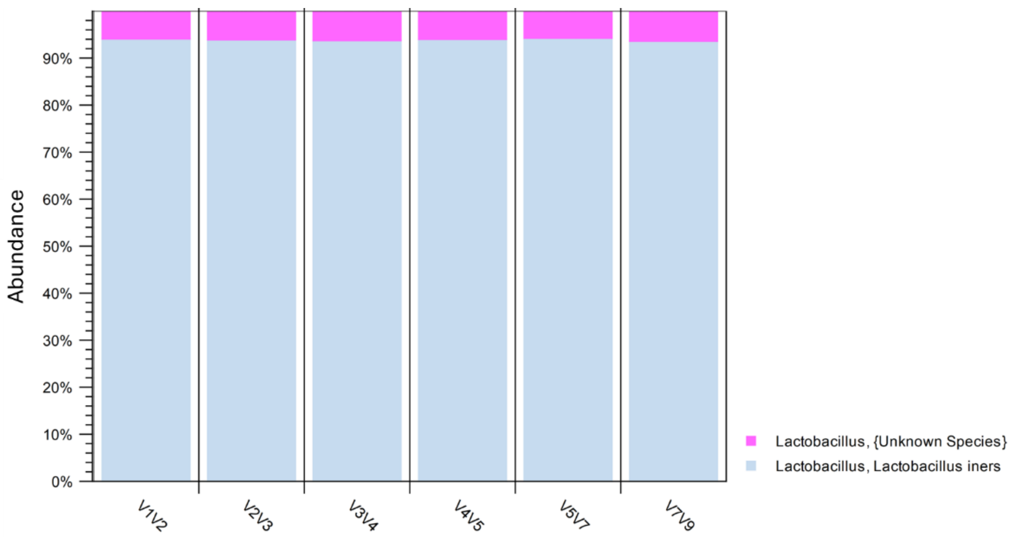

AO

Sample ID:
30663-017
LSIL
HPV-POS

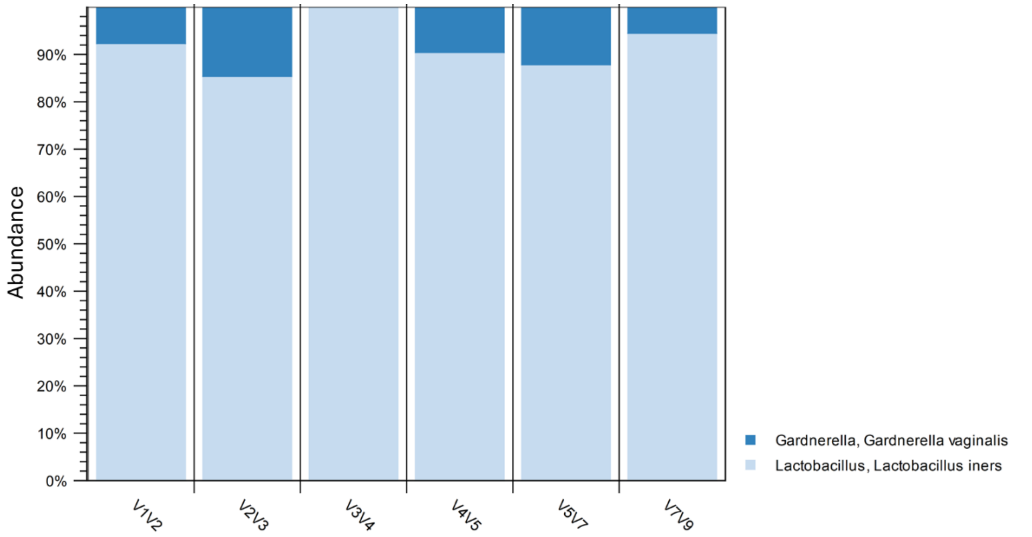

AP

Sample ID:
30663-018
LSIL
HPV-POS

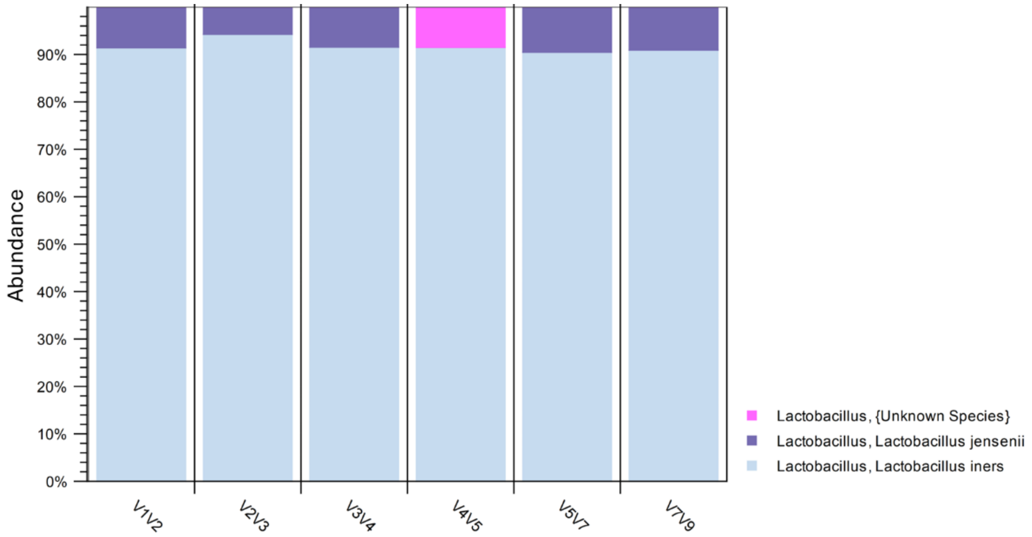

AQ

Sample ID:
30663-019
LSIL
HPV-POS

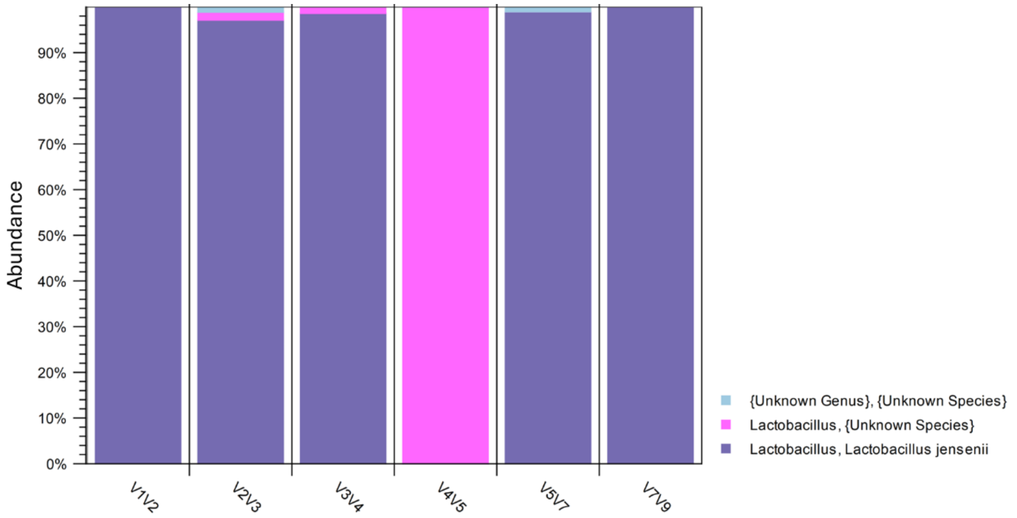

AR

Sample ID:
30663-020
LSIL
HPV-POS

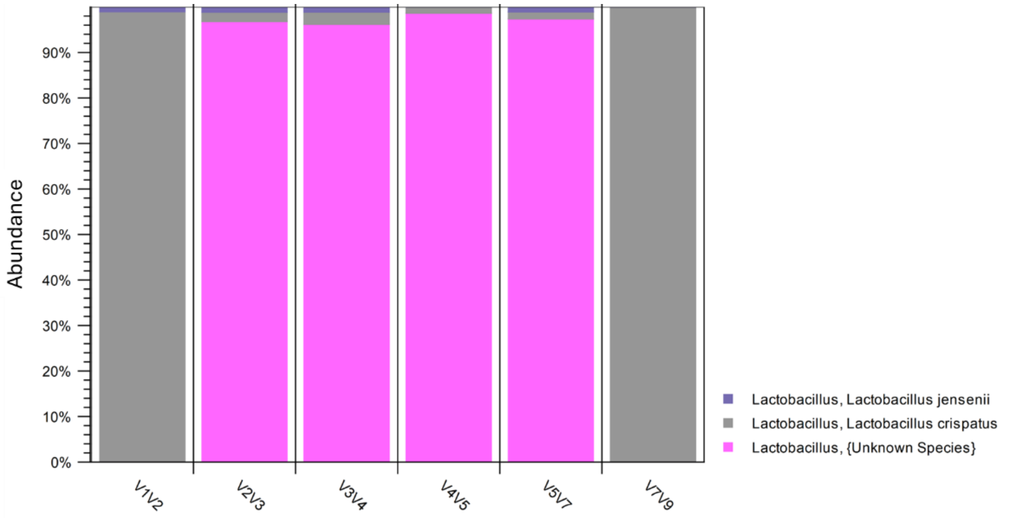

AS

Sample ID:
30663-021
LSIL
HPV-POS

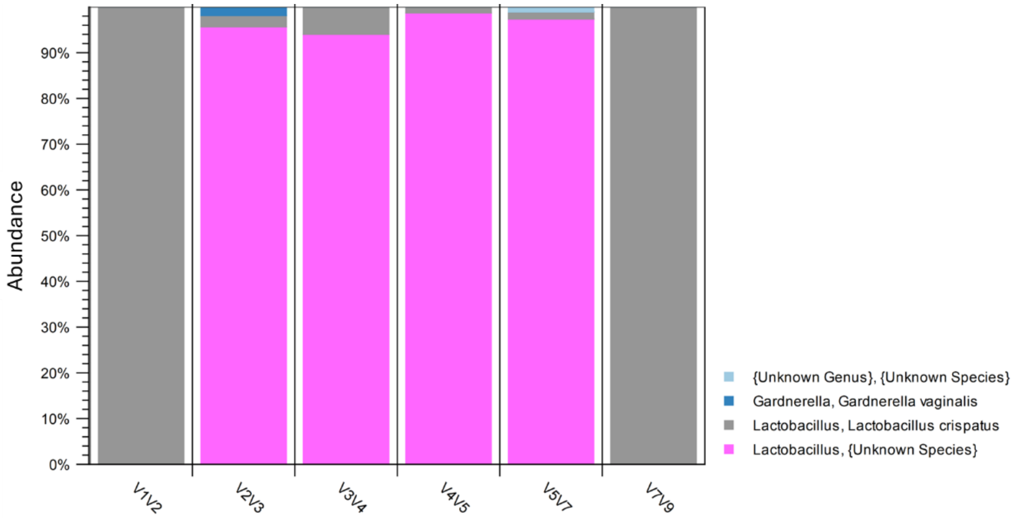

AT

Sample ID:
30663-022
LSIL
HPV-POS

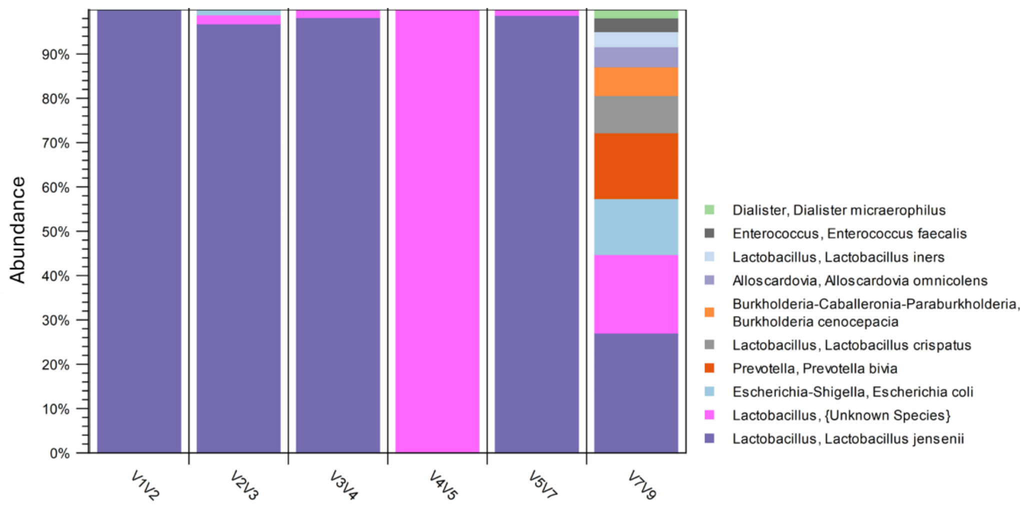

AU

Sample ID:
30663-023
LSIL
HPV-POS

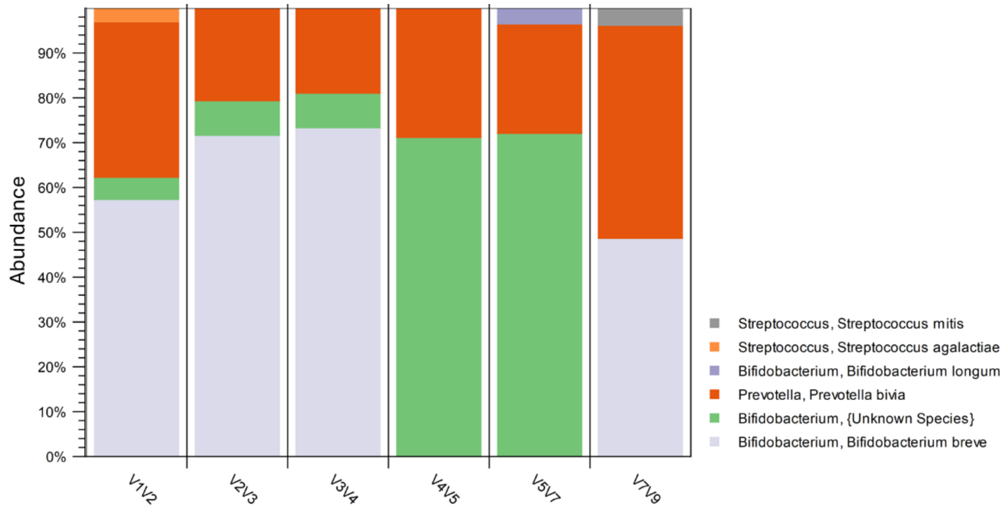

AV

Sample ID:
30663-024
LSIL
HPV-POS

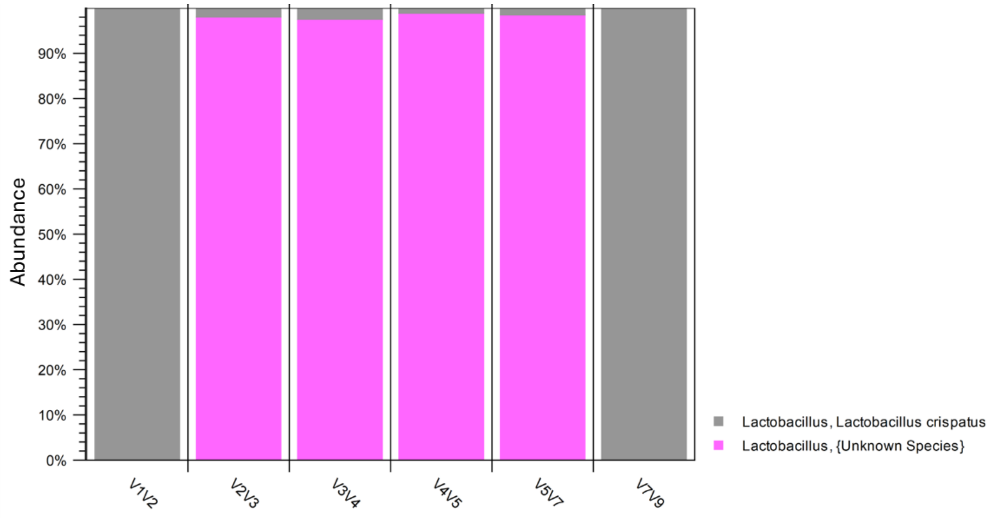

AW

Sample ID:
30663-025
HSIL
HPV-POS

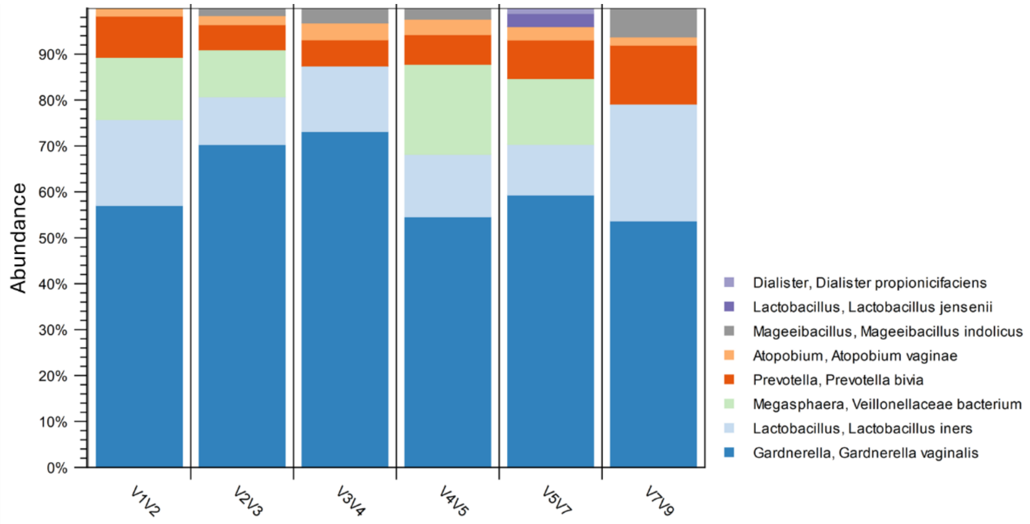

AX

Sample ID:
30663-027
HSIL
HPV-POS

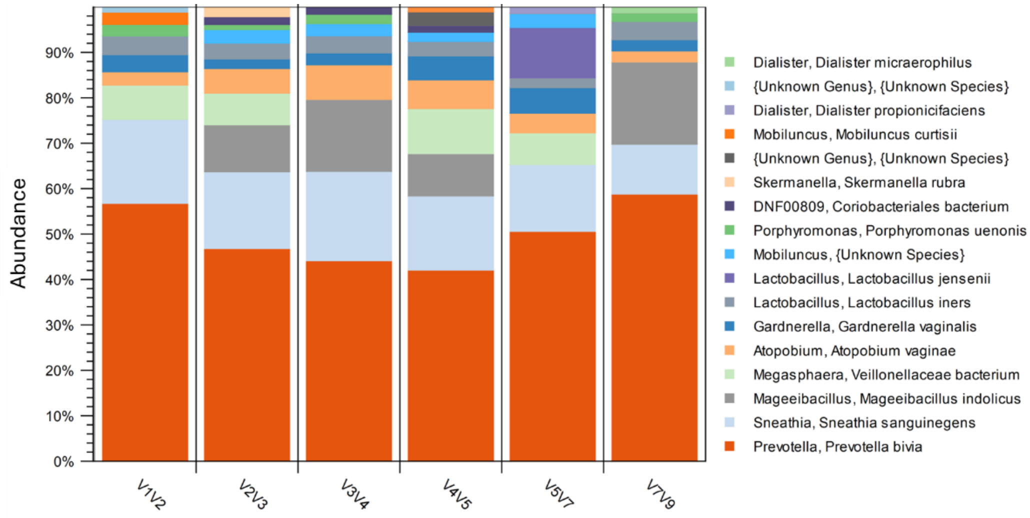

AY

Sample ID:
30663-028
HSIL
HPV-POS

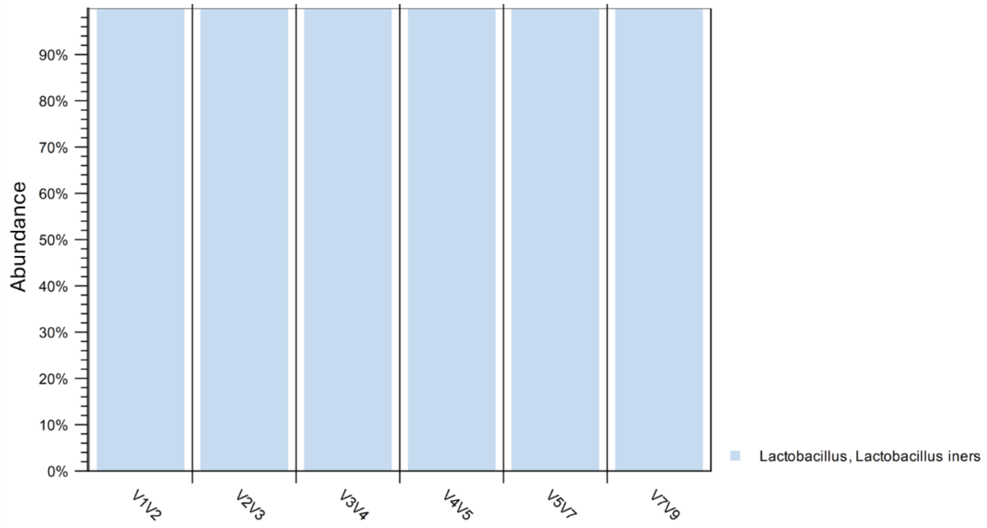

AZ

Sample ID:
30663-029
HSIL
HPV-POS

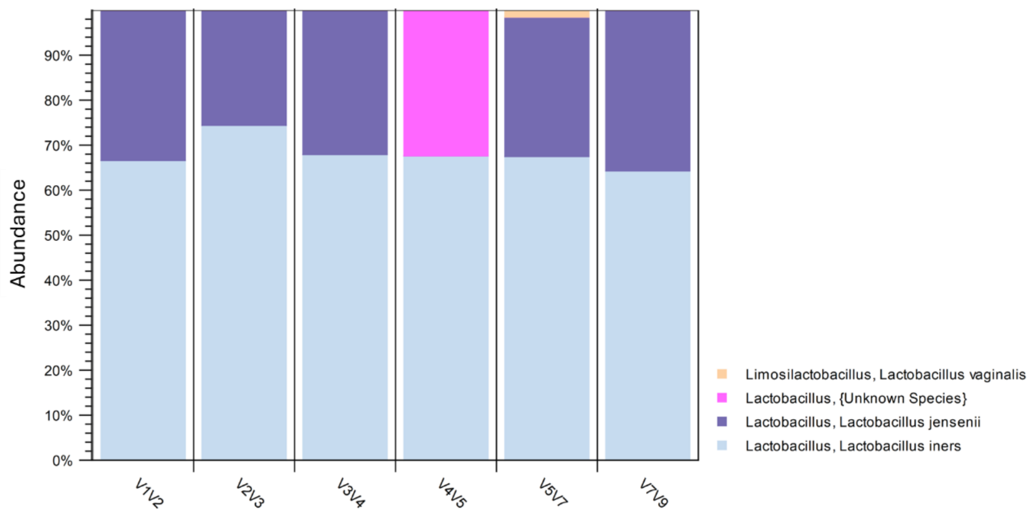

BA

Sample ID:
30663-030
HSIL
HPV-POS

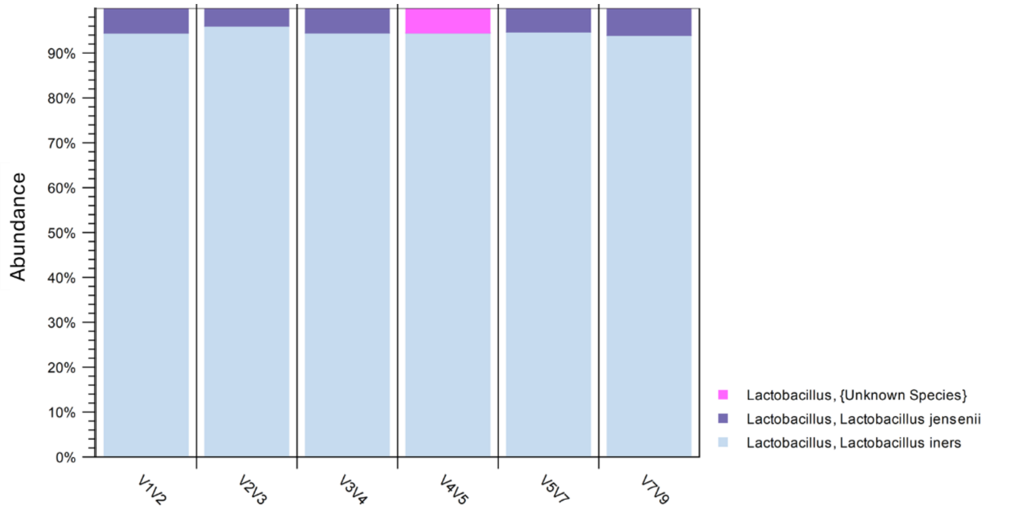

BB

Sample ID:
30663-032
HSIL
HPV-POS

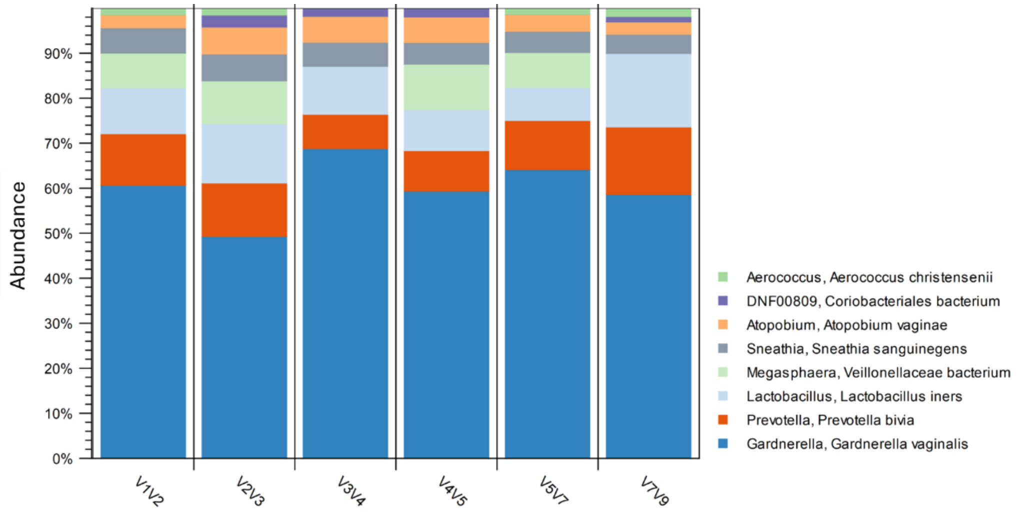

BC

Sample ID:
30663-033
HSIL
HPV-POS

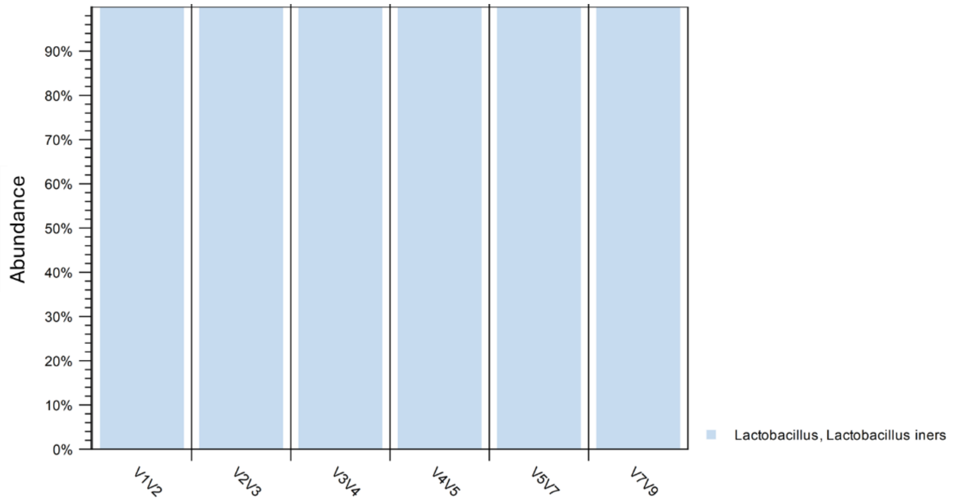

BD

Sample ID:
30663-034
HSIL
HPV-POS

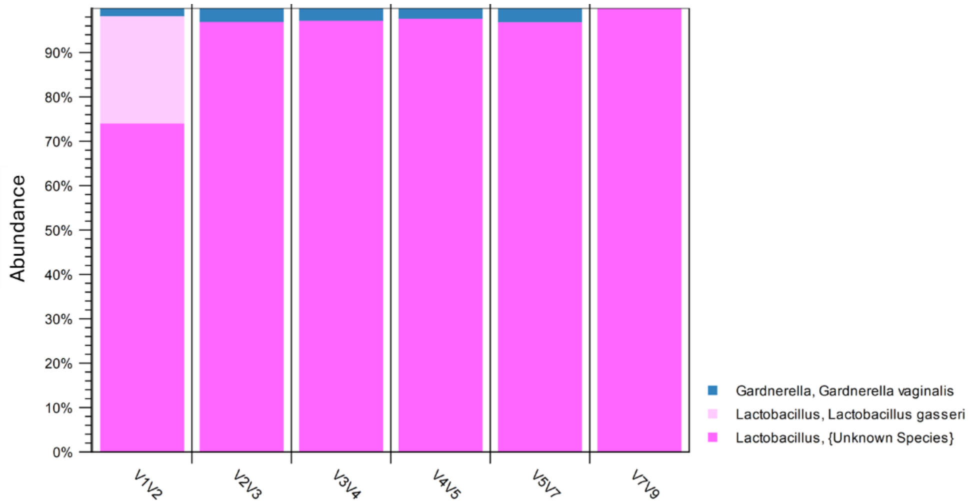

BE

Sample ID:
30663-036
HSIL
HPV-POS

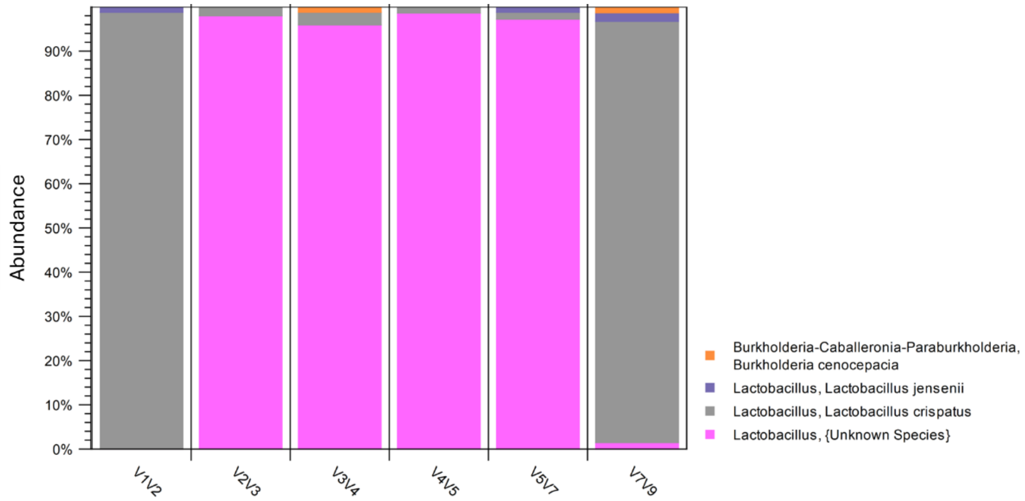

BF

Sample ID:
30663-037
HSIL
HPV-POS

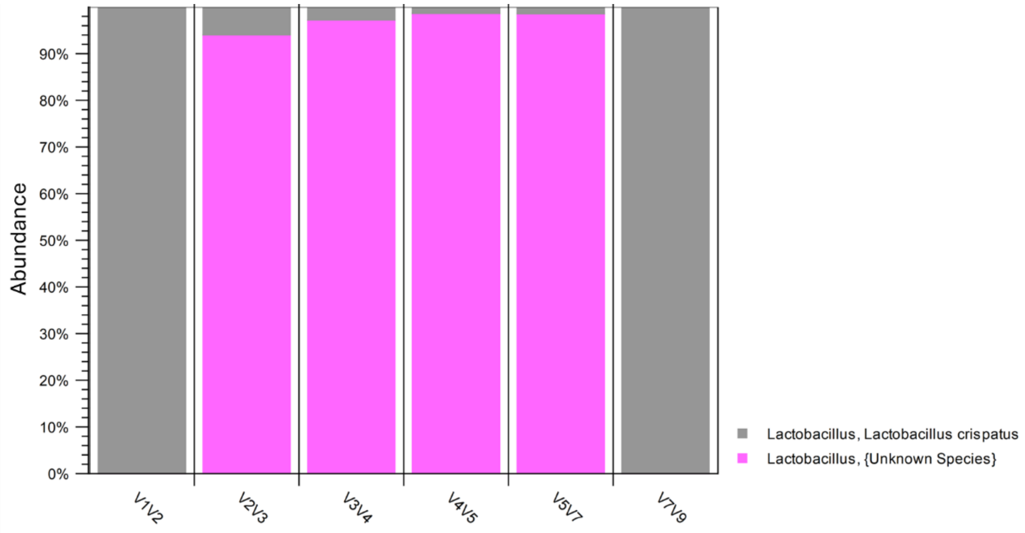

BG

Sample ID:
30663-038
HSIL
HPV-POS

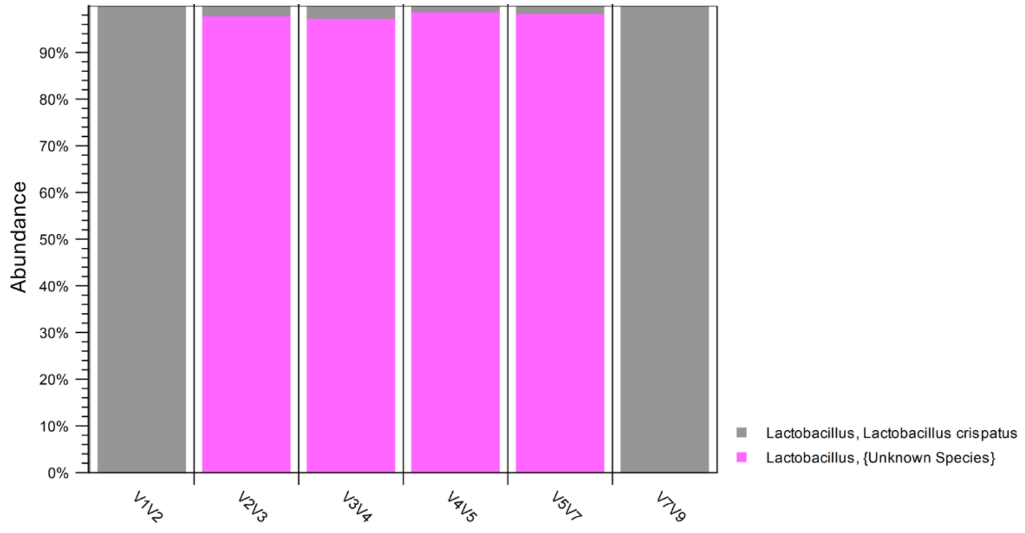

BH

Sample ID:
30663-039
HSIL
HPV-POS

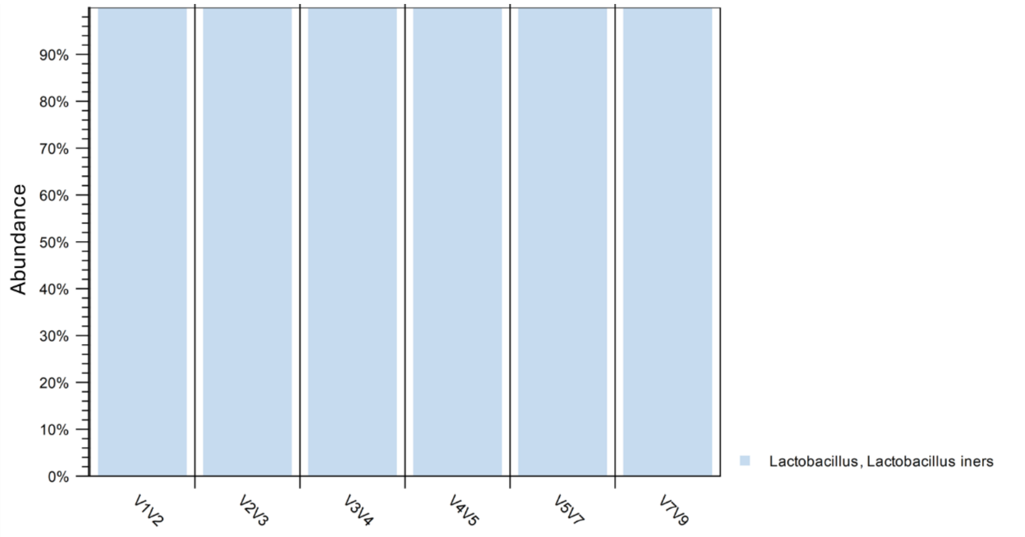

BI

Sample ID:
30663-040
HSIL
HPV-POS

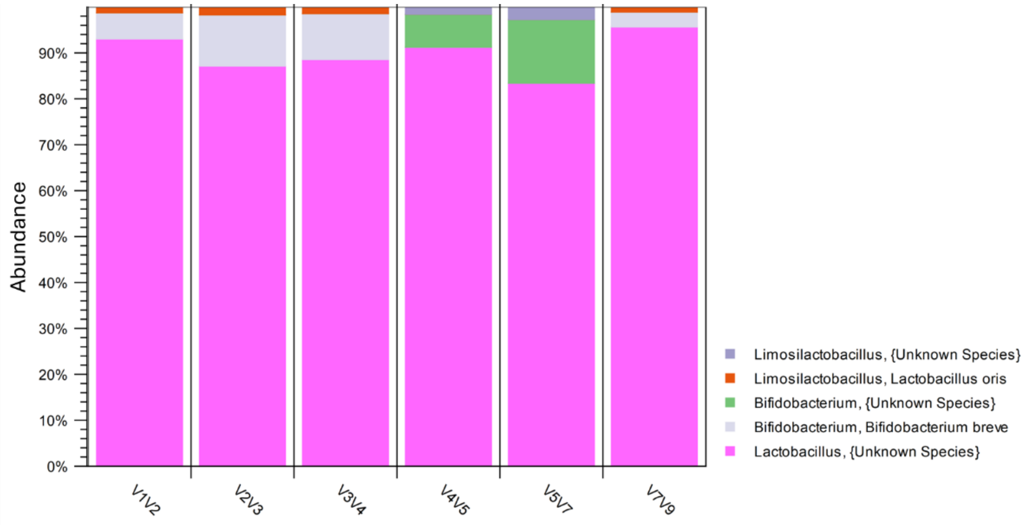

BJ

Sample ID:
30663-041
HSIL
HPV-POS

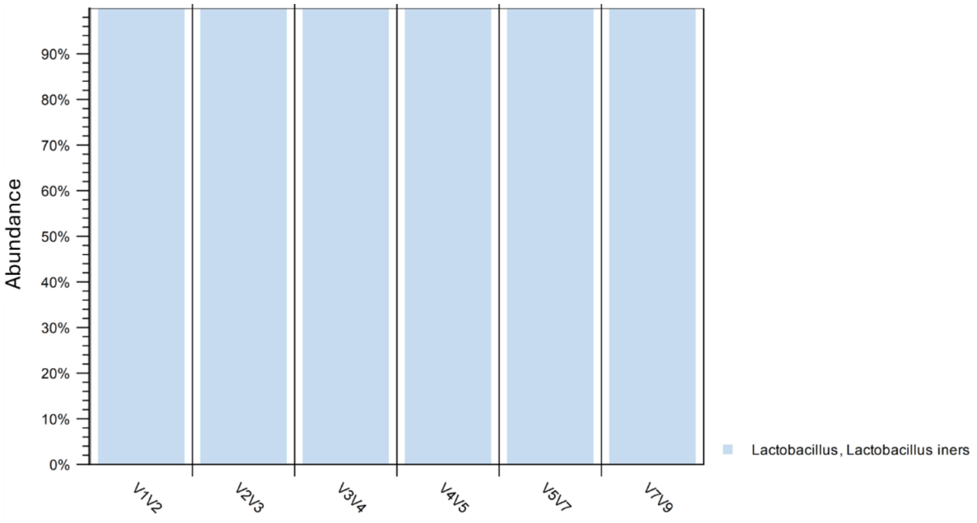

BK

Sample ID:
30663-042
HSIL
HPV-POS

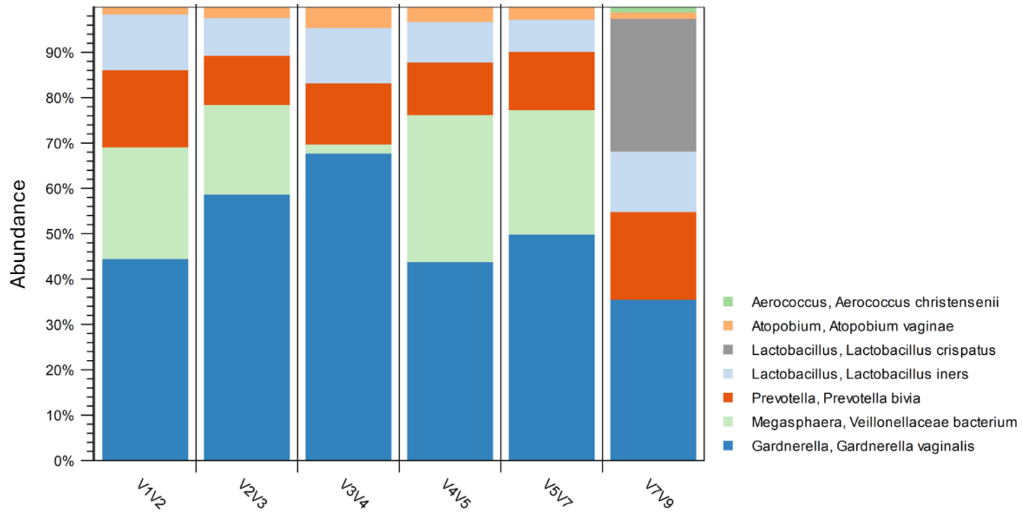

BL

Sample ID:
30663-043
HSIL
HPV-POS

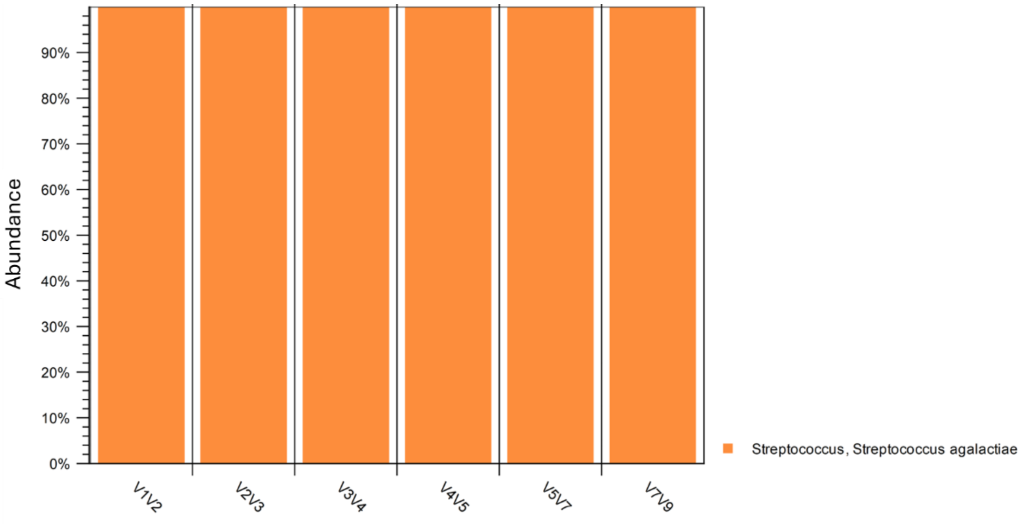

BM

Sample ID:
30663-044
HSIL
HPV-POS

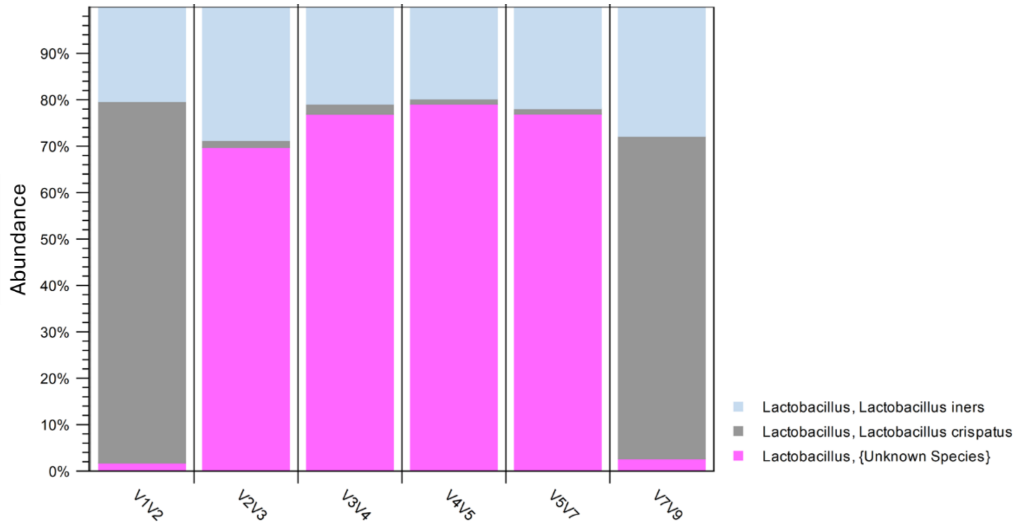

BN

Sample ID:
30663-045
HSIL
HPV-POS

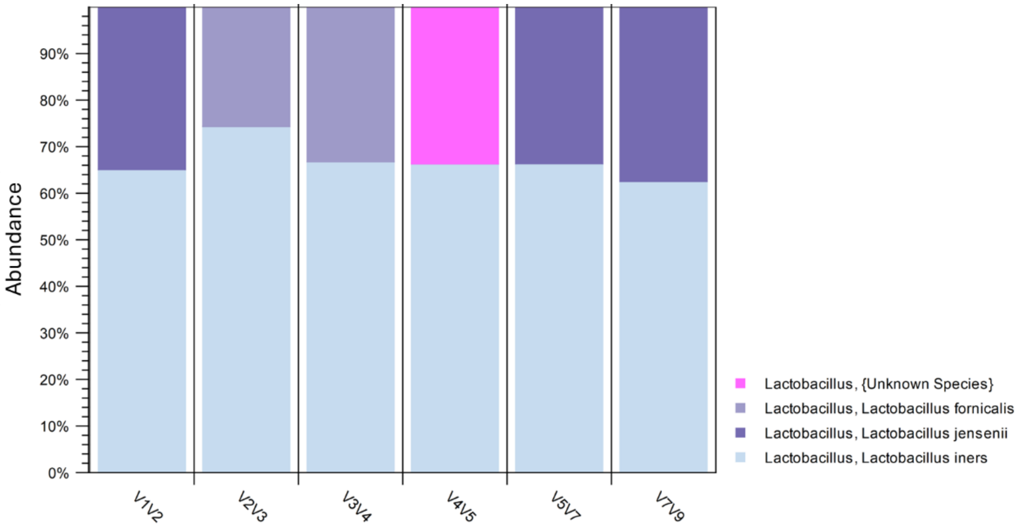

BO

Sample ID:
30663-046
HSIL
HPV-POS

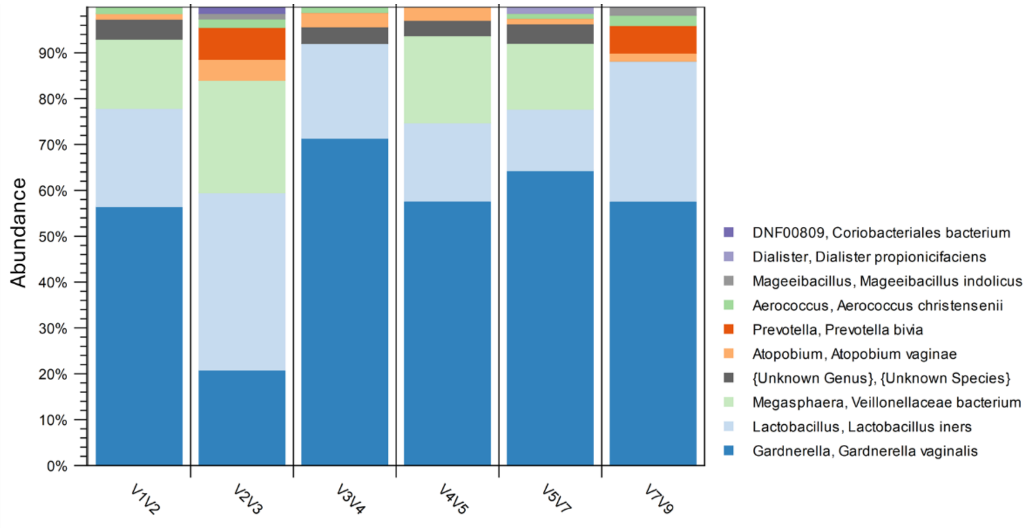

BP

Sample ID:
30663-047
HSIL
HPV-POS

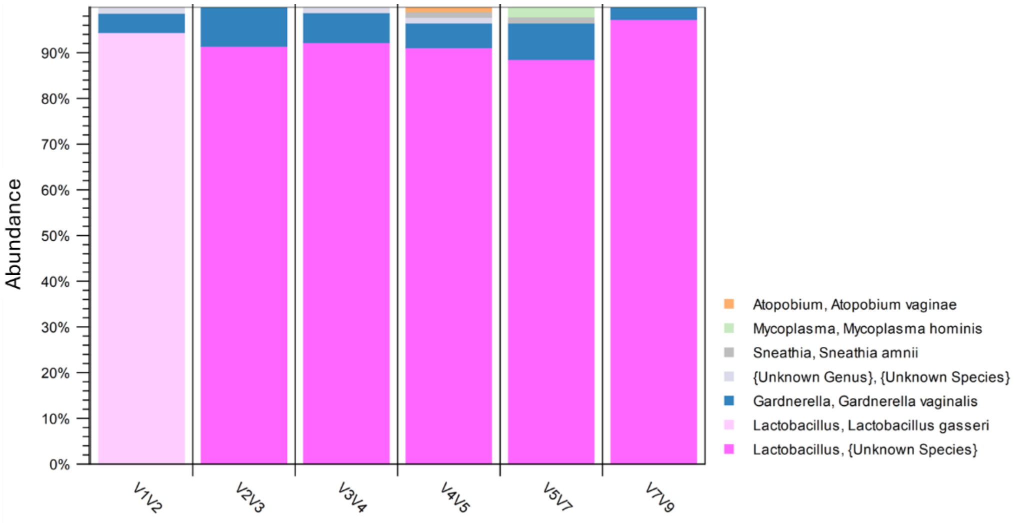

BQ

Sample ID:
30663-049
HSIL
HPV-POS

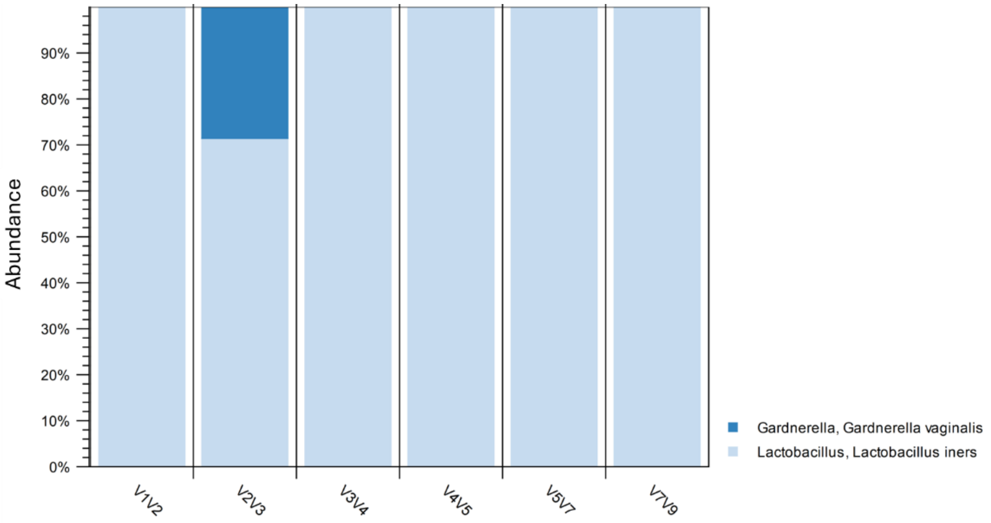

Supplementary Figure S1. Taxonomic composition of 66 cytology samples and 2 genomic standards. (**A–V, Y-BQ**) Each panel displays the sample ID, cytological diagnosis, and HPV status alongside a stacked bar chart illustrating the relative abundance of microbial species identified through 16S V1–V9 hypervariable region sequencing. (**W,X**) Stacked bar charts display the microbial composition of replicate genomic standards. The legend denotes the identified microbial genera and species.
