# Supplementary material for: Cervicovaginal Microbiome and HPV: A Standardized Approach to 16S/ITS NGS and Microbial Community Profiling for Viral Association
Source: Int J Mol Sci. 2025 Aug 21;26(16):8090. doi: 10.3390/ijms26168090 (PMC12386612; doi:10.3390/ijms26168090)
Supplement: Supplementary file 1 [file ijms-26-08090-s001.zip › ijms-3725007_SUPP_R1_/FIG S2_FUNGAL ITS READS, N-7.pdf]

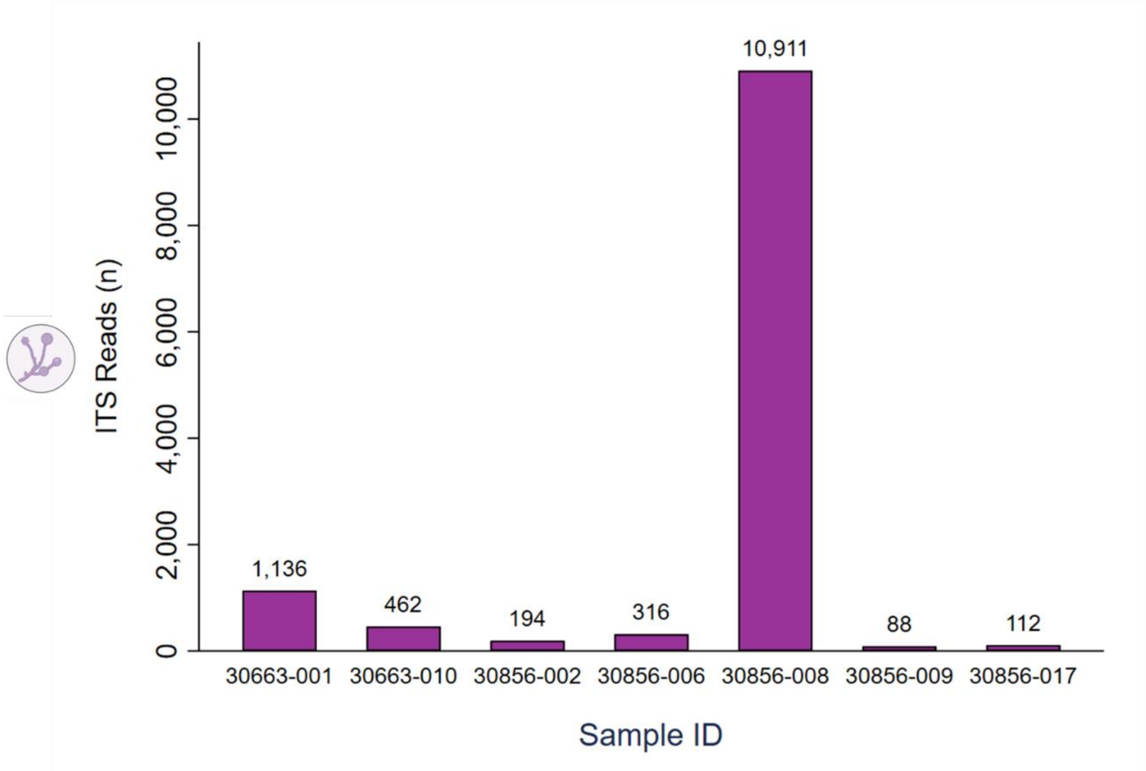

Supplementary Figure S2. Fungal taxonomic composition of cytology samples based on ITS sequencing. The bar chart shows the relative abundance of detected fungal species. *Candida albicans* was the only species identified with sufficient read depth in 7 out of 66 samples. ITS read counts, indicated on each bar, ranged from 88 to 10,911. The ITS read counts were significantly lower than those observed with 16S sequencing.
