# Supplementary material for: Cervicovaginal Microbiome and HPV: A Standardized Approach to 16S/ITS NGS and Microbial Community Profiling for Viral Association
Source: Int J Mol Sci. 2025 Aug 21;26(16):8090. doi: 10.3390/ijms26168090 (PMC12386612; doi:10.3390/ijms26168090)
Supplement: Supplementary file 1 [file ijms-26-08090-s001.zip › ijms-3725007_SUPP_R1_/TABLE S1_SUPP QC REPORT 30856-002.pdf]

Table of contents

1. Summary 3
2. Per-sequence analysis 3
    2.1 Lengths distribution 3
    2.2 GC-content 9
    2.3 Ambiguous base-content 11
    2.4 Quality distribution 12
3. Per-base analysis 13
    3.1 Coverage 14
    3.2 Nucleotide contributions 21
    3.3 GC-content 52
    3.4 Ambiguous base-content 59
    3.5 Quality distribution 67
4. Over-representation analyses 112
    4.1 Enriched 5-mers 112
    4.2 Sequence duplication levels 143
    4.3 Duplicated sequences 152

1. Summary

Creation date:	Fri Sep 30 09:32:29 CDT 2022
Generated by:	Jane
Software:	CLC Genomics Workbench 22.0.2
Based upon:	1 data set
30856-002 (paired):	3,028,632 sequences in pairs
Total sequences in data set	3,028,632 sequences
Total nucleotides in data set	832,421,592 nucleotides

2. Per-sequence analysis

2.1 Lengths distribution

Summarizes the frequencies of observed sequence lengths.
sequence length: number of bases that make up the sequence
abs: number of sequences observed at that sequence length
%: number of sequences observed at that sequence length normalized to the total number sequences in R1 / R2

sequence length	R1: abs	R1: %	R2: abs	R2: %
35	1	0.00	0	0.00
36	0	0.00	1	0.00
37	1	0.00	0	0.00
38	1	0.00	0	0.00
39	0	0.00	1	0.00
40	4	0.00	0	0.00
41	26	0.00	4	0.00
42	18	0.00	26	0.00
43	14	0.00	18	0.00
44	27	0.00	14	0.00
45	36	0.00	26	0.00
46	43	0.00	36	0.00
47	40	0.00	42	0.00
48	27	0.00	41	0.00

sequence length	R1: abs	R1: %	R2: abs	R2: %
49	32	0.00	26	0.00
50	41	0.00	31	0.00
51	24	0.00	42	0.00
52	27	0.00	25	0.00
53	18	0.00	27	0.00
54	12	0.00	18	0.00
55	3	0.00	12	0.00
56	7	0.00	3	0.00
57	2	0.00	7	0.00
58	0	0.00	2	0.00
59	2	0.00	0	0.00
60	1	0.00	2	0.00
61	0	0.00	1	0.00
64	4	0.00	0	0.00
65	3	0.00	4	0.00
66	1	0.00	3	0.00
67	1	0.00	1	0.00
68	3	0.00	1	0.00
69	2	0.00	3	0.00
70	0	0.00	2	0.00
71	1	0.00	0	0.00
72	0	0.00	1	0.00
73	0	0.00	1	0.00
78	0	0.00	1	0.00
99	1	0.00	0	0.00
101	0	0.00	1	0.00
102	1	0.00	1	0.00
103	1	0.00	0	0.00
104	1	0.00	1	0.00
105	0	0.00	1	0.00
111	1	0.00	0	0.00
112	0	0.00	1	0.00
114	11	0.00	0	0.00
115	15	0.00	10	0.00
116	31	0.00	14	0.00
117	54	0.00	28	0.00
118	42	0.00	58	0.00

sequence length	R1: abs	R1: %	R2: abs	R2: %
119	61	0.00	40	0.00
120	71	0.00	62	0.00
121	76	0.01	71	0.00
122	95	0.01	72	0.00
123	128	0.01	96	0.01
124	114	0.01	130	0.01
125	125	0.01	113	0.01
126	118	0.01	126	0.01
127	119	0.01	121	0.01
128	94	0.01	119	0.01
129	73	0.00	93	0.01
130	79	0.01	73	0.00
131	62	0.00	80	0.01
132	56	0.00	59	0.00
133	38	0.00	57	0.00
134	25	0.00	35	0.00
135	10	0.00	27	0.00
136	11	0.00	10	0.00
137	7	0.00	11	0.00
138	9	0.00	6	0.00
139	5	0.00	10	0.00
140	5	0.00	5	0.00
141	8	0.00	6	0.00
142	3	0.00	7	0.00
143	6	0.00	3	0.00
144	0	0.00	6	0.00
145	2	0.00	0	0.00
146	1	0.00	2	0.00
147	1	0.00	1	0.00
148	0	0.00	2	0.00
151	19	0.00	0	0.00
152	1	0.00	20	0.00
153	0	0.00	1	0.00
155	3	0.00	0	0.00
156	0	0.00	3	0.00
157	5	0.00	0	0.00
158	8	0.00	5	0.00

sequence length	R1: abs	R1: %	R2: abs	R2: %
159	13	0.00	8	0.00
160	33	0.00	15	0.00
161	56	0.00	32	0.00
162	67	0.00	56	0.00
163	92	0.01	65	0.00
164	130	0.01	90	0.01
165	155	0.01	126	0.01
166	180	0.01	154	0.01
167	215	0.01	180	0.01
168	229	0.02	211	0.01
169	272	0.02	232	0.02
170	298	0.02	269	0.02
171	318	0.02	301	0.02
172	323	0.02	320	0.02
173	282	0.02	320	0.02
174	256	0.02	280	0.02
175	244	0.02	256	0.02
176	195	0.01	242	0.02
177	164	0.01	192	0.01
178	124	0.01	163	0.01
179	148	0.01	127	0.01
180	71	0.00	143	0.01
181	59	0.00	69	0.00
182	45	0.00	59	0.00
183	13	0.00	44	0.00
184	13	0.00	13	0.00
185	3	0.00	12	0.00
186	4	0.00	3	0.00
187	2	0.00	6	0.00
188	7	0.00	1	0.00
189	68	0.00	6	0.00
190	71	0.00	65	0.00
191	50	0.00	68	0.00
192	503	0.03	43	0.00
193	487	0.03	483	0.03
194	346	0.02	483	0.03
195	279	0.02	318	0.02

sequence length	R1: abs	R1: %	R2: abs	R2: %
196	572	0.04	264	0.02
197	585	0.04	555	0.04
198	390	0.03	574	0.04
199	351	0.02	361	0.02
200	102	0.01	340	0.02
201	133	0.01	100	0.01
202	105	0.01	127	0.01
203	79	0.01	103	0.01
204	47	0.00	73	0.00
205	1	0.00	42	0.00
206	0	0.00	1	0.00
207	2	0.00	0	0.00
208	16	0.00	3	0.00
209	263	0.02	10	0.00
210	235	0.02	259	0.02
211	145	0.01	226	0.01
212	136	0.01	129	0.01
213	280	0.02	132	0.01
214	622	0.04	270	0.02
215	500	0.03	596	0.04
216	425	0.03	460	0.03
217	181	0.01	389	0.03
218	369	0.02	178	0.01
219	400	0.03	354	0.02
220	274	0.02	386	0.03
221	208	0.01	245	0.02
222	1	0.00	201	0.01
223	2	0.00	1	0.00
224	1	0.00	4	0.00
225	0	0.00	4	0.00
226	2	0.00	0	0.00
227	4	0.00	1	0.00
228	0	0.00	4	0.00
229	4	0.00	3	0.00
230	11	0.00	5	0.00
231	9	0.00	11	0.00
232	11	0.00	10	0.00

sequence length	R1: abs	R1: %	R2: abs	R2: %
233	9	0.00	9	0.00
234	7	0.00	10	0.00
235	4	0.00	7	0.00
236	3	0.00	12	0.00
237	9	0.00	12	0.00
238	6	0.00	14	0.00
239	8	0.00	7	0.00
240	3	0.00	8	0.00
241	8	0.00	1	0.00
242	4	0.00	7	0.00
243	3	0.00	4	0.00
244	4	0.00	4	0.00
245	4	0.00	4	0.00
246	0	0.00	5	0.00
247	1	0.00	0	0.00
248	2	0.00	1	0.00
249	1	0.00	2	0.00
250	3	0.00	0	0.00
251	10	0.00	3	0.00
252	5	0.00	11	0.00
253	6	0.00	2	0.00
254	8	0.00	7	0.00
255	10	0.00	10	0.00
256	10	0.00	11	0.00
257	7	0.00	9	0.00
258	13	0.00	7	0.00
259	19	0.00	15	0.00
260	23	0.00	17	0.00
261	18	0.00	22	0.00
262	26	0.00	16	0.00
263	15	0.00	24	0.00
264	22	0.00	13	0.00
265	27	0.00	22	0.00
266	25	0.00	25	0.00
267	21	0.00	21	0.00
268	20	0.00	18	0.00
269	22	0.00	19	0.00

sequence length	R1: abs	R1: %	R2: abs	R2: %
270	17	0.00	29	0.00
271	397	0.03	61	0.00
272	446	0.03	315	0.02
273	1,518	0.10	4,104	0.27
274	62,229	4.11	51,541	3.40
275	381,464	25.19	202,091	13.35
276	1,053,560	69.57	1,241,911	82.01

2.2 GC-content

Summarizes the distribution of GC-contents. The GC-content of a sequence is calculated as the number of GC-bases compared to all bases (including ambiguous bases).
% GC-content: relative GC-content of a sequence in percent
abs: number of sequences featuring particular GC-percentages
%: number of sequences featuring particular GC-percentages normalized to the total number of sequences in R1 / R2

% GC-content	R1: abs	R1: %	R2: abs	R2: %
20	0	0.00	1	0.00
27	0	0.00	2	0.00
28	2	0.00	0	0.00
30	0	0.00	2	0.00
31	1	0.00	3	0.00
32	2	0.00	2	0.00
33	5	0.00	5	0.00
34	2	0.00	9	0.00
35	2	0.00	12	0.00
36	15	0.00	84	0.01
37	208	0.01	186	0.01
38	56	0.00	52	0.00
39	57	0.00	85	0.01
40	91	0.01	201	0.01
41	355	0.02	526	0.03
42	2,733	0.18	680	0.04
43	48,477	3.20	1,488	0.10
44	45,067	2.98	4,699	0.31
45	22,778	1.50	28,451	1.88
46	19,620	1.30	65,801	4.35

% GC-content	R1: abs	R1: %	R2: abs	R2: %
47	108,683	7.18	53,427	3.53
48	100,899	6.66	49,699	3.28
49	205,076	13.54	145,551	9.61
50	128,273	8.47	162,694	10.74
51	187,266	12.37	168,288	11.11
52	172,614	11.40	171,569	11.33
53	142,155	9.39	231,919	15.32
54	71,596	4.73	133,444	8.81
55	44,564	2.94	79,418	5.24
56	14,146	0.93	45,559	3.01
57	34,264	2.26	32,121	2.12
58	61,943	4.09	23,101	1.53
59	72,677	4.80	18,361	1.21
60	11,275	0.74	23,173	1.53
61	14,909	0.98	43,676	2.88
62	3,273	0.22	21,771	1.44
63	1,119	0.07	5,903	0.39
64	62	0.00	1,443	0.10
65	17	0.00	338	0.02
66	9	0.00	202	0.01
67	5	0.00	88	0.01
68	11	0.00	63	0.00
69	5	0.00	20	0.00
70	1	0.00	26	0.00
71	0	0.00	18	0.00
72	1	0.00	25	0.00
73	0	0.00	11	0.00
74	0	0.00	13	0.00
75	0	0.00	6	0.00
76	2	0.00	5	0.00
77	0	0.00	4	0.00
78	0	0.00	7	0.00
79	0	0.00	5	0.00
80	0	0.00	3	0.00
81	0	0.00	2	0.00
82	0	0.00	8	0.00
83	0	0.00	4	0.00

% GC-content	R1: abs	R1: %	R2: abs	R2: %
84	0	0.00	6	0.00
85	0	0.00	3	0.00
86	0	0.00	7	0.00
87	0	0.00	3	0.00
88	0	0.00	4	0.00
89	0	0.00	3	0.00
90	0	0.00	2	0.00
91	0	0.00	3	0.00
92	0	0.00	2	0.00
93	0	0.00	5	0.00
94	0	0.00	3	0.00
95	0	0.00	3	0.00
96	0	0.00	1	0.00
97	0	0.00	4	0.00
99	0	0.00	3	0.00
100	0	0.00	10	0.00

2.3 Ambiguous base-content

Summarizes the distribution of N-contents. The N-content of a sequence is calculated as the number of ambiguous bases compared to all bases.
% ambiguous bases: relative N-content of a sequence in percent
abs: number of sequences featuring particular N-percentages
%: number of sequences featuring particular N-percentages normalized to the total number of sequences in R1 / R2

% ambiguous bases	R1: abs	R1: %	R2: abs	R2: %
0	1,513,798	99.97	1,513,462	99.94
1	277	0.02	534	0.04
2	62	0.00	36	0.00
3	43	0.00	36	0.00
4	20	0.00	22	0.00
5	10	0.00	17	0.00
6	10	0.00	2	0.00
7	5	0.00	13	0.00
8	17	0.00	16	0.00
9	10	0.00	8	0.00
10	9	0.00	16	0.00

% ambiguous bases	R1: abs	R1: %	R2: abs	R2: %
11	10	0.00	6	0.00
12	5	0.00	9	0.00
13	6	0.00	10	0.00
14	6	0.00	7	0.00
15	4	0.00	6	0.00
16	2	0.00	15	0.00
17	1	0.00	10	0.00
18	2	0.00	4	0.00
19	0	0.00	5	0.00
20	2	0.00	4	0.00
21	2	0.00	7	0.00
22	2	0.00	5	0.00
23	1	0.00	7	0.00
24	0	0.00	7	0.00
25	2	0.00	5	0.00
26	3	0.00	3	0.00
27	1	0.00	3	0.00
28	1	0.00	8	0.00
29	0	0.00	4	0.00
30	0	0.00	1	0.00
31	0	0.00	9	0.00
32	1	0.00	5	0.00
33	1	0.00	1	0.00
34	0	0.00	5	0.00
35	1	0.00	1	0.00
37	0	0.00	1	0.00
38	1	0.00	2	0.00
42	0	0.00	2	0.00
43	1	0.00	0	0.00
44	0	0.00	1	0.00
55	0	0.00	1	0.00

2.4 Quality distribution

Summarizes the distribution of average sequence quality scores. The quality of a sequence is calculated as the arithmetic mean of its base qualities.
average quality: PHRED-score

abs: number of sequences observed at that qual. score
%: number of sequences observed at that qual. score normalized to the total number of sequences in R1 / R2

average PHRED score	R1: abs	R1: %	R2: abs	R2: %
11	0	0.00	2	0.00
12	0	0.00	13	0.00
13	0	0.00	113	0.01
14	1	0.00	587	0.04
15	6	0.00	1,752	0.12
16	20	0.00	3,066	0.20
17	126	0.01	4,529	0.30
18	229	0.02	6,191	0.41
19	381	0.03	7,766	0.51
20	583	0.04	10,022	0.66
21	838	0.06	12,600	0.83
22	1,235	0.08	15,310	1.01
23	1,888	0.12	18,991	1.25
24	2,702	0.18	22,341	1.48
25	3,765	0.25	27,320	1.80
26	5,180	0.34	32,633	2.15
27	7,308	0.48	38,321	2.53
28	10,029	0.66	45,627	3.01
29	13,415	0.89	54,847	3.62
30	17,712	1.17	67,348	4.45
31	23,244	1.53	83,024	5.48
32	30,445	2.01	105,352	6.96
33	40,388	2.67	137,093	9.05
34	55,184	3.64	182,077	12.02
35	80,977	5.35	241,401	15.94
36	135,633	8.96	275,972	18.22
37	361,357	23.86	115,047	7.60
38	721,670	47.66	4,971	0.33

3. Per-base analysis

3.1 Coverage

Summarizes the number of sequences that support (cover) the individual base positions.
abs: number of sequences covering individual base positions
%: number of sequences covering individual base positions normalized to the total number of sequences

base position	R1: abs	R1: %	R2: abs	R2: %
1	1,514,316	50.00	1,514,316	50.00
2	1,514,316	50.00	1,514,316	50.00
3	1,514,316	50.00	1,514,316	50.00
4	1,514,316	50.00	1,514,316	50.00
5	1,514,316	50.00	1,514,316	50.00
6	1,514,316	50.00	1,514,316	50.00
7	1,514,316	50.00	1,514,316	50.00
8	1,514,316	50.00	1,514,316	50.00
9	1,514,316	50.00	1,514,316	50.00
10	1,514,316	50.00	1,514,316	50.00
11	1,514,316	50.00	1,514,316	50.00
12	1,514,316	50.00	1,514,316	50.00
13	1,514,316	50.00	1,514,316	50.00
14	1,514,316	50.00	1,514,316	50.00
15	1,514,316	50.00	1,514,316	50.00
16	1,514,316	50.00	1,514,316	50.00
17	1,514,316	50.00	1,514,316	50.00
18	1,514,316	50.00	1,514,316	50.00
19	1,514,316	50.00	1,514,316	50.00
20	1,514,316	50.00	1,514,316	50.00
21	1,514,316	50.00	1,514,316	50.00
22	1,514,316	50.00	1,514,316	50.00
23	1,514,316	50.00	1,514,316	50.00
24	1,514,316	50.00	1,514,316	50.00
25	1,514,316	50.00	1,514,316	50.00
26	1,514,316	50.00	1,514,316	50.00
27	1,514,316	50.00	1,514,316	50.00
28	1,514,316	50.00	1,514,316	50.00
29	1,514,316	50.00	1,514,316	50.00
30	1,514,316	50.00	1,514,316	50.00
31	1,514,316	50.00	1,514,316	50.00

base position	R1: abs	R1: %	R2: abs	R2: %
32	1,514,316	50.00	1,514,316	50.00
33	1,514,316	50.00	1,514,316	50.00
34	1,514,316	50.00	1,514,316	50.00
35	1,514,316	50.00	1,514,316	50.00
36	1,514,315	50.00	1,514,316	50.00
37	1,514,315	50.00	1,514,315	50.00
38	1,514,314	50.00	1,514,315	50.00
39	1,514,313	50.00	1,514,315	50.00
40	1,514,313	50.00	1,514,314	50.00
41	1,514,309	50.00	1,514,314	50.00
42	1,514,283	50.00	1,514,310	50.00
43	1,514,265	50.00	1,514,284	50.00
44	1,514,251	50.00	1,514,266	50.00
45	1,514,224	50.00	1,514,252	50.00
46	1,514,188	50.00	1,514,226	50.00
47	1,514,145	49.99	1,514,190	50.00
48	1,514,105	49.99	1,514,148	49.99
49	1,514,078	49.99	1,514,107	49.99
50	1,514,046	49.99	1,514,081	49.99
51	1,514,005	49.99	1,514,050	49.99
52	1,513,981	49.99	1,514,008	49.99
53	1,513,954	49.99	1,513,983	49.99
54	1,513,936	49.99	1,513,956	49.99
55	1,513,924	49.99	1,513,938	49.99
56	1,513,921	49.99	1,513,926	49.99
57	1,513,914	49.99	1,513,923	49.99
58	1,513,912	49.99	1,513,916	49.99
59	1,513,912	49.99	1,513,914	49.99
60	1,513,910	49.99	1,513,914	49.99
61	1,513,909	49.99	1,513,912	49.99
62	1,513,909	49.99	1,513,911	49.99
63	1,513,909	49.99	1,513,911	49.99
64	1,513,909	49.99	1,513,911	49.99
65	1,513,905	49.99	1,513,911	49.99
66	1,513,902	49.99	1,513,907	49.99
67	1,513,901	49.99	1,513,904	49.99
68	1,513,900	49.99	1,513,903	49.99

base position	R1: abs	R1: %	R2: abs	R2: %
69	1,513,897	49.99	1,513,902	49.99
70	1,513,895	49.99	1,513,899	49.99
71	1,513,895	49.99	1,513,897	49.99
72	1,513,894	49.99	1,513,897	49.99
73	1,513,894	49.99	1,513,896	49.99
74	1,513,894	49.99	1,513,895	49.99
75	1,513,894	49.99	1,513,895	49.99
76	1,513,894	49.99	1,513,895	49.99
77	1,513,894	49.99	1,513,895	49.99
78	1,513,894	49.99	1,513,895	49.99
79	1,513,894	49.99	1,513,894	49.99
80	1,513,894	49.99	1,513,894	49.99
81	1,513,894	49.99	1,513,894	49.99
82	1,513,894	49.99	1,513,894	49.99
83	1,513,894	49.99	1,513,894	49.99
84	1,513,894	49.99	1,513,894	49.99
85	1,513,894	49.99	1,513,894	49.99
86	1,513,894	49.99	1,513,894	49.99
87	1,513,894	49.99	1,513,894	49.99
88	1,513,894	49.99	1,513,894	49.99
89	1,513,894	49.99	1,513,894	49.99
90	1,513,894	49.99	1,513,894	49.99
91	1,513,894	49.99	1,513,894	49.99
92	1,513,894	49.99	1,513,894	49.99
93	1,513,894	49.99	1,513,894	49.99
94	1,513,894	49.99	1,513,894	49.99
95	1,513,894	49.99	1,513,894	49.99
96	1,513,894	49.99	1,513,894	49.99
97	1,513,894	49.99	1,513,894	49.99
98	1,513,894	49.99	1,513,894	49.99
99	1,513,894	49.99	1,513,894	49.99
100	1,513,893	49.99	1,513,894	49.99
101	1,513,893	49.99	1,513,894	49.99
102	1,513,893	49.99	1,513,893	49.99
103	1,513,892	49.99	1,513,892	49.99
104	1,513,891	49.99	1,513,892	49.99
105	1,513,890	49.99	1,513,891	49.99

base position	R1: abs	R1: %	R2: abs	R2: %
106	1,513,890	49.99	1,513,890	49.99
107	1,513,890	49.99	1,513,890	49.99
108	1,513,890	49.99	1,513,890	49.99
109	1,513,890	49.99	1,513,890	49.99
110	1,513,890	49.99	1,513,890	49.99
111	1,513,890	49.99	1,513,890	49.99
112	1,513,889	49.99	1,513,890	49.99
113	1,513,889	49.99	1,513,889	49.99
114	1,513,889	49.99	1,513,889	49.99
115	1,513,878	49.99	1,513,889	49.99
116	1,513,863	49.99	1,513,879	49.99
117	1,513,832	49.98	1,513,865	49.99
118	1,513,778	49.98	1,513,837	49.98
119	1,513,736	49.98	1,513,779	49.98
120	1,513,675	49.98	1,513,739	49.98
121	1,513,604	49.98	1,513,677	49.98
122	1,513,528	49.97	1,513,606	49.98
123	1,513,433	49.97	1,513,534	49.97
124	1,513,305	49.97	1,513,438	49.97
125	1,513,191	49.96	1,513,308	49.97
126	1,513,066	49.96	1,513,195	49.96
127	1,512,948	49.95	1,513,069	49.96
128	1,512,829	49.95	1,512,948	49.95
129	1,512,735	49.95	1,512,829	49.95
130	1,512,662	49.95	1,512,736	49.95
131	1,512,583	49.94	1,512,663	49.95
132	1,512,521	49.94	1,512,583	49.94
133	1,512,465	49.94	1,512,524	49.94
134	1,512,427	49.94	1,512,467	49.94
135	1,512,402	49.94	1,512,432	49.94
136	1,512,392	49.94	1,512,405	49.94
137	1,512,381	49.94	1,512,395	49.94
138	1,512,374	49.94	1,512,384	49.94
139	1,512,365	49.94	1,512,378	49.94
140	1,512,360	49.94	1,512,368	49.94
141	1,512,355	49.94	1,512,363	49.94
142	1,512,347	49.93	1,512,357	49.94

base position	R1: abs	R1: %	R2: abs	R2: %
143	1,512,344	49.93	1,512,350	49.94
144	1,512,338	49.93	1,512,347	49.93
145	1,512,338	49.93	1,512,341	49.93
146	1,512,336	49.93	1,512,341	49.93
147	1,512,335	49.93	1,512,339	49.93
148	1,512,334	49.93	1,512,338	49.93
149	1,512,334	49.93	1,512,336	49.93
150	1,512,334	49.93	1,512,336	49.93
151	1,512,334	49.93	1,512,336	49.93
152	1,512,315	49.93	1,512,336	49.93
153	1,512,314	49.93	1,512,316	49.93
154	1,512,314	49.93	1,512,315	49.93
155	1,512,314	49.93	1,512,315	49.93
156	1,512,311	49.93	1,512,315	49.93
157	1,512,311	49.93	1,512,312	49.93
158	1,512,306	49.93	1,512,312	49.93
159	1,512,298	49.93	1,512,307	49.93
160	1,512,285	49.93	1,512,299	49.93
161	1,512,252	49.93	1,512,284	49.93
162	1,512,196	49.93	1,512,252	49.93
163	1,512,129	49.93	1,512,196	49.93
164	1,512,037	49.92	1,512,131	49.93
165	1,511,907	49.92	1,512,041	49.92
166	1,511,752	49.92	1,511,915	49.92
167	1,511,572	49.91	1,511,761	49.92
168	1,511,357	49.90	1,511,581	49.91
169	1,511,128	49.89	1,511,370	49.90
170	1,510,856	49.89	1,511,138	49.90
171	1,510,558	49.88	1,510,869	49.89
172	1,510,240	49.87	1,510,568	49.88
173	1,509,917	49.85	1,510,248	49.87
174	1,509,635	49.85	1,509,928	49.86
175	1,509,379	49.84	1,509,648	49.85
176	1,509,135	49.83	1,509,392	49.84
177	1,508,940	49.82	1,509,150	49.83
178	1,508,776	49.82	1,508,958	49.82
179	1,508,652	49.81	1,508,795	49.82

base position	R1: abs	R1: %	R2: abs	R2: %
180	1,508,504	49.81	1,508,668	49.81
181	1,508,433	49.81	1,508,525	49.81
182	1,508,374	49.80	1,508,456	49.81
183	1,508,329	49.80	1,508,397	49.80
184	1,508,316	49.80	1,508,353	49.80
185	1,508,303	49.80	1,508,340	49.80
186	1,508,300	49.80	1,508,328	49.80
187	1,508,296	49.80	1,508,325	49.80
188	1,508,294	49.80	1,508,319	49.80
189	1,508,287	49.80	1,508,318	49.80
190	1,508,219	49.80	1,508,312	49.80
191	1,508,148	49.80	1,508,247	49.80
192	1,508,098	49.79	1,508,179	49.80
193	1,507,595	49.78	1,508,136	49.80
194	1,507,108	49.76	1,507,653	49.78
195	1,506,762	49.75	1,507,170	49.76
196	1,506,483	49.74	1,506,852	49.75
197	1,505,911	49.72	1,506,588	49.74
198	1,505,326	49.70	1,506,033	49.73
199	1,504,936	49.69	1,505,459	49.71
200	1,504,585	49.68	1,505,098	49.70
201	1,504,483	49.68	1,504,758	49.68
202	1,504,350	49.67	1,504,658	49.68
203	1,504,245	49.67	1,504,531	49.68
204	1,504,166	49.66	1,504,428	49.67
205	1,504,119	49.66	1,504,355	49.67
206	1,504,118	49.66	1,504,313	49.67
207	1,504,118	49.66	1,504,312	49.67
208	1,504,116	49.66	1,504,312	49.67
209	1,504,100	49.66	1,504,309	49.67
210	1,503,837	49.65	1,504,299	49.67
211	1,503,602	49.65	1,504,040	49.66
212	1,503,457	49.64	1,503,814	49.65
213	1,503,321	49.64	1,503,685	49.65
214	1,503,041	49.63	1,503,553	49.64
215	1,502,419	49.61	1,503,283	49.64
216	1,501,919	49.59	1,502,687	49.62

base position	R1: abs	R1: %	R2: abs	R2: %
217	1,501,494	49.58	1,502,227	49.60
218	1,501,313	49.57	1,501,838	49.59
219	1,500,944	49.56	1,501,660	49.58
220	1,500,544	49.55	1,501,306	49.57
221	1,500,270	49.54	1,500,920	49.56
222	1,500,062	49.53	1,500,675	49.55
223	1,500,061	49.53	1,500,474	49.54
224	1,500,059	49.53	1,500,473	49.54
225	1,500,058	49.53	1,500,469	49.54
226	1,500,058	49.53	1,500,465	49.54
227	1,500,056	49.53	1,500,465	49.54
228	1,500,052	49.53	1,500,464	49.54
229	1,500,052	49.53	1,500,460	49.54
230	1,500,048	49.53	1,500,457	49.54
231	1,500,037	49.53	1,500,452	49.54
232	1,500,028	49.53	1,500,441	49.54
233	1,500,017	49.53	1,500,431	49.54
234	1,500,008	49.53	1,500,422	49.54
235	1,500,001	49.53	1,500,412	49.54
236	1,499,997	49.53	1,500,405	49.54
237	1,499,994	49.53	1,500,393	49.54
238	1,499,985	49.53	1,500,381	49.54
239	1,499,979	49.53	1,500,367	49.54
240	1,499,971	49.53	1,500,360	49.54
241	1,499,968	49.53	1,500,352	49.54
242	1,499,960	49.53	1,500,351	49.54
243	1,499,956	49.53	1,500,344	49.54
244	1,499,953	49.53	1,500,340	49.54
245	1,499,949	49.53	1,500,336	49.54
246	1,499,945	49.53	1,500,332	49.54
247	1,499,945	49.53	1,500,327	49.54
248	1,499,944	49.53	1,500,327	49.54
249	1,499,942	49.53	1,500,326	49.54
250	1,499,941	49.53	1,500,324	49.54
251	1,499,938	49.53	1,500,324	49.54
252	1,499,928	49.52	1,500,321	49.54
253	1,499,923	49.52	1,500,310	49.54

base position	R1: abs	R1: %	R2: abs	R2: %
254	1,499,917	49.52	1,500,308	49.54
255	1,499,909	49.52	1,500,301	49.54
256	1,499,899	49.52	1,500,291	49.54
257	1,499,889	49.52	1,500,280	49.54
258	1,499,882	49.52	1,500,271	49.54
259	1,499,869	49.52	1,500,264	49.54
260	1,499,850	49.52	1,500,249	49.54
261	1,499,827	49.52	1,500,232	49.53
262	1,499,809	49.52	1,500,210	49.53
263	1,499,783	49.52	1,500,194	49.53
264	1,499,768	49.52	1,500,170	49.53
265	1,499,746	49.52	1,500,157	49.53
266	1,499,719	49.52	1,500,135	49.53
267	1,499,694	49.52	1,500,110	49.53
268	1,499,673	49.52	1,500,089	49.53
269	1,499,653	49.52	1,500,071	49.53
270	1,499,631	49.52	1,500,052	49.53
271	1,499,614	49.51	1,500,023	49.53
272	1,499,217	49.50	1,499,962	49.53
273	1,498,771	49.49	1,499,647	49.52
274	1,497,253	49.44	1,495,543	49.38
275	1,435,024	47.38	1,444,002	47.68
276	1,053,560	34.79	1,241,911	41.01

3.2 Nucleotide contributions

R1

Summarizes the coverages for the four DNA nucleotides and ambiguous bases for R1 reads.
coverage: total number of bases observed at that base position
% ACTGN: number of nucleotides observed per type and base position normalized to the total number of nucleotides observed at that position

base position	coverage	% Adenine	% Thymine	% Cytosine
1	1,514,316	21.49	21.76	33.49
2	1,514,316	20.87	21.39	35.80
3	1,514,316	20.52	35.50	22.20
4	1,514,316	34.54	23.59	20.77

base position	coverage	% Adenine	% Thymine	% Cytosine
5	1,514,316	21.25	19.13	33.61
6	1,514,316	22.77	21.44	17.92
7	1,514,316	20.77	19.30	21.98
8	1,514,316	20.41	19.20	19.62
9	1,514,316	24.46	19.24	21.96
10	1,514,316	22.13	15.59	15.10
11	1,514,316	19.72	16.66	18.98
12	1,514,316	18.93	14.59	31.98
13	1,514,316	24.84	22.27	21.71
14	1,514,316	19.24	14.77	23.87
15	1,514,316	17.04	17.16	32.91
16	1,514,316	29.88	15.44	21.61
17	1,514,316	20.01	17.70	20.79
18	1,514,316	18.77	29.65	22.26
19	1,514,316	25.50	16.87	19.45
20	1,514,316	23.62	19.54	21.69
21	1,514,316	21.69	19.52	21.08
22	1,514,316	20.86	21.34	22.48
23	1,514,316	34.69	19.27	21.78
24	1,514,316	35.78	22.06	19.55
25	1,514,316	24.84	32.52	18.81
26	1,514,316	34.56	21.48	23.42
27	1,514,316	28.96	30.86	20.23
28	1,514,316	27.38	31.73	18.93
29	1,514,316	23.68	17.65	27.67
30	1,514,316	23.89	18.17	29.44
31	1,514,316	29.22	22.13	24.48
32	1,514,316	25.40	19.10	32.27
33	1,514,316	38.80	18.06	23.86
34	1,514,316	37.85	15.08	25.70
35	1,514,316	23.82	26.30	28.08
36	1,514,315	22.18	15.67	27.56
37	1,514,315	21.95	19.15	26.97
38	1,514,314	30.69	20.88	25.06
39	1,514,313	23.91	20.15	28.36
40	1,514,313	21.50	22.83	23.53
41	1,514,309	28.41	23.92	23.82

base position	coverage	% Adenine	% Thymine	% Cytosine
42	1,514,283	34.14	23.37	21.71
43	1,514,265	32.45	23.68	22.32
44	1,514,251	19.91	23.04	28.03
45	1,514,224	22.05	31.79	20.59
46	1,514,188	23.30	23.48	26.37
47	1,514,145	24.56	35.05	14.78
48	1,514,105	24.79	22.60	14.33
49	1,514,078	37.79	23.35	14.89
50	1,514,046	27.43	29.27	18.71
51	1,514,005	23.62	24.17	23.10
52	1,513,981	25.94	22.29	22.58
53	1,513,954	37.51	21.18	15.73
54	1,513,936	26.41	20.56	14.39
55	1,513,924	24.41	20.13	27.17
56	1,513,921	29.36	21.17	19.18
57	1,513,914	34.70	21.33	16.08
58	1,513,912	27.96	24.03	22.61
59	1,513,912	28.01	23.09	14.67
60	1,513,910	31.33	19.57	24.30
61	1,513,909	33.37	16.56	21.76
62	1,513,909	30.30	13.64	16.73
63	1,513,909	29.88	18.05	23.52
64	1,513,909	28.20	15.99	16.83
65	1,513,905	26.99	29.18	15.75
66	1,513,902	25.85	17.41	17.02
67	1,513,901	29.90	17.83	23.80
68	1,513,900	27.13	19.21	17.63
69	1,513,897	26.22	25.55	19.06
70	1,513,895	24.56	19.40	17.93
71	1,513,895	34.55	21.94	18.09
72	1,513,894	29.85	27.17	18.00
73	1,513,894	23.52	22.23	18.86
74	1,513,894	34.16	22.35	19.20
75	1,513,894	31.29	21.83	21.01
76	1,513,894	21.80	21.77	17.59
77	1,513,894	24.38	20.95	17.68
78	1,513,894	27.05	26.51	20.97

base position	coverage	% Adenine	% Thymine	% Cytosine
79	1,513,894	26.64	29.22	19.81
80	1,513,894	28.81	30.30	17.97
81	1,513,894	28.92	32.15	15.27
82	1,513,894	33.61	20.51	20.90
83	1,513,894	28.19	21.82	14.85
84	1,513,894	28.48	20.80	15.17
85	1,513,894	32.16	22.20	15.68
86	1,513,894	27.57	27.67	16.13
87	1,513,894	28.93	29.45	16.35
88	1,513,894	29.85	25.14	11.67
89	1,513,894	27.71	33.08	12.01
90	1,513,894	37.46	24.30	11.69
91	1,513,894	36.87	24.05	13.22
92	1,513,894	33.33	24.03	14.41
93	1,513,894	28.76	23.55	18.67
94	1,513,894	21.98	22.86	29.04
95	1,513,894	26.63	27.04	17.51
96	1,513,894	22.87	21.58	27.26
97	1,513,894	22.99	30.61	22.10
98	1,513,894	22.20	28.05	21.39
99	1,513,894	21.93	32.32	21.05
100	1,513,893	21.56	29.90	23.63
101	1,513,893	27.02	20.15	23.79
102	1,513,893	23.65	22.90	23.39
103	1,513,892	22.49	27.65	22.93
104	1,513,891	30.19	19.65	21.73
105	1,513,890	21.03	22.62	20.22
106	1,513,890	22.09	21.59	19.05
107	1,513,890	23.86	18.78	18.02
108	1,513,890	35.07	17.79	17.72
109	1,513,890	34.65	17.20	18.29
110	1,513,890	30.70	16.29	18.89
111	1,513,890	37.65	18.38	17.94
112	1,513,889	38.03	16.49	20.06
113	1,513,889	36.21	17.56	20.28
114	1,513,889	37.46	17.87	19.80
115	1,513,878	34.43	17.89	20.73

base position	coverage	% Adenine	% Thymine	% Cytosine
116	1,513,863	33.21	20.57	20.85
117	1,513,832	32.84	20.90	19.40
118	1,513,778	26.41	22.21	22.49
119	1,513,736	25.20	21.43	20.44
120	1,513,675	28.58	19.86	20.58
121	1,513,604	24.52	22.00	24.15
122	1,513,528	26.40	23.80	20.10
123	1,513,433	24.97	21.90	23.93
124	1,513,305	23.66	25.72	22.57
125	1,513,191	28.91	25.45	22.14
126	1,513,066	27.71	20.16	24.90
127	1,512,948	28.88	21.79	27.07
128	1,512,829	28.98	24.11	24.91
129	1,512,735	33.78	20.32	25.29
130	1,512,662	30.81	19.46	26.45
131	1,512,583	35.40	17.75	23.88
132	1,512,521	28.97	22.56	22.40
133	1,512,465	33.86	23.20	20.75
134	1,512,427	31.56	24.51	20.18
135	1,512,402	28.82	24.97	18.13
136	1,512,392	31.47	21.56	19.05
137	1,512,381	29.93	20.22	21.84
138	1,512,374	28.87	22.96	19.50
139	1,512,365	22.78	24.59	22.87
140	1,512,360	25.39	24.71	19.41
141	1,512,355	24.83	25.05	18.61
142	1,512,347	26.13	21.56	22.76
143	1,512,344	19.94	22.73	24.80
144	1,512,338	21.07	21.10	21.78
145	1,512,338	22.12	20.88	24.27
146	1,512,336	18.83	29.96	21.98
147	1,512,335	27.71	19.88	21.41
148	1,512,334	24.55	19.45	24.57
149	1,512,334	23.62	18.62	26.48
150	1,512,334	23.44	23.51	24.28
151	1,512,334	30.33	20.87	21.64
152	1,512,315	27.72	21.35	23.86

base position	coverage	% Adenine	% Thymine	% Cytosine
153	1,512,314	27.00	18.82	23.61
154	1,512,314	26.27	23.06	23.85
155	1,512,314	28.52	18.85	18.30
156	1,512,311	25.81	16.39	21.73
157	1,512,311	31.08	16.89	21.52
158	1,512,306	33.02	18.11	17.79
159	1,512,298	27.59	18.51	16.41
160	1,512,285	22.79	18.50	17.60
161	1,512,252	26.30	19.74	15.70
162	1,512,196	24.73	18.83	16.67
163	1,512,129	29.04	21.09	15.02
164	1,512,037	26.47	19.20	17.95
165	1,511,907	26.41	19.09	11.90
166	1,511,752	24.44	22.30	15.82
167	1,511,572	23.93	22.54	17.78
168	1,511,357	23.42	24.99	17.56
169	1,511,128	26.87	25.68	17.57
170	1,510,856	32.18	21.80	17.00
171	1,510,558	25.67	24.84	23.26
172	1,510,240	22.62	30.07	18.89
173	1,509,917	31.61	23.80	21.19
174	1,509,635	26.25	21.89	27.96
175	1,509,379	27.63	21.27	19.34
176	1,509,135	24.23	27.48	19.12
177	1,508,940	26.82	20.70	22.63
178	1,508,776	26.48	23.51	24.48
179	1,508,652	25.11	19.21	24.32
180	1,508,504	30.42	21.37	23.12
181	1,508,433	29.03	20.23	21.29
182	1,508,374	30.60	15.86	22.52
183	1,508,329	32.38	16.91	23.79
184	1,508,316	27.90	16.86	19.93
185	1,508,303	30.11	16.62	23.20
186	1,508,300	26.25	21.23	22.93
187	1,508,296	29.27	19.38	22.90
188	1,508,294	32.14	17.23	22.22
189	1,508,287	30.58	24.13	17.21

base position	coverage	% Adenine	% Thymine	% Cytosine
190	1,508,219	31.35	20.45	18.49
191	1,508,148	29.87	21.38	20.85
192	1,508,098	30.59	20.47	17.62
193	1,507,595	30.22	18.38	22.07
194	1,507,108	29.04	24.10	21.71
195	1,506,762	31.88	22.28	17.81
196	1,506,483	26.10	23.32	21.11
197	1,505,911	28.97	22.26	19.22
198	1,505,326	29.61	26.63	19.98
199	1,504,936	30.67	24.57	20.55
200	1,504,585	26.38	22.57	21.11
201	1,504,483	31.75	16.70	18.45
202	1,504,350	27.81	24.29	18.37
203	1,504,245	28.31	22.36	18.07
204	1,504,166	27.53	20.01	20.41
205	1,504,119	26.57	18.63	20.77
206	1,504,118	31.90	20.61	17.08
207	1,504,118	31.47	19.62	20.79
208	1,504,116	30.08	19.16	17.67
209	1,504,100	25.77	22.02	21.13
210	1,503,837	26.79	17.52	20.07
211	1,503,602	24.86	21.53	22.20
212	1,503,457	23.58	22.63	21.00
213	1,503,321	21.51	20.14	21.10
214	1,503,041	25.90	22.34	21.69
215	1,502,419	26.27	19.19	25.22
216	1,501,919	26.06	19.59	26.18
217	1,501,494	29.23	20.48	20.23
218	1,501,313	26.83	21.02	21.07
219	1,500,944	29.26	17.79	22.91
220	1,500,544	28.47	21.17	20.86
221	1,500,270	30.09	18.26	22.34
222	1,500,062	26.87	21.02	20.37
223	1,500,061	29.41	19.12	19.74
224	1,500,059	24.90	26.51	22.14
225	1,500,058	23.29	27.50	19.73
226	1,500,058	23.28	23.09	18.43

base position	coverage	% Adenine	% Thymine	% Cytosine
227	1,500,056	25.19	23.71	18.08
228	1,500,052	29.52	19.63	19.00
229	1,500,052	30.89	18.74	23.79
230	1,500,048	25.58	19.32	19.97
231	1,500,037	28.49	23.57	19.00
232	1,500,028	27.61	19.80	22.62
233	1,500,017	32.81	20.26	20.04
234	1,500,008	31.15	18.01	20.74
235	1,500,001	26.21	17.36	21.48
236	1,499,997	25.97	21.10	25.07
237	1,499,994	26.65	23.55	21.70
238	1,499,985	28.59	20.29	23.62
239	1,499,979	27.85	19.60	23.21
240	1,499,971	26.10	22.91	24.79
241	1,499,968	20.73	21.96	27.53
242	1,499,960	21.07	23.93	30.54
243	1,499,956	24.91	26.90	26.19
244	1,499,953	20.87	24.57	26.91
245	1,499,949	23.45	24.23	27.54
246	1,499,945	22.52	25.96	28.08
247	1,499,945	22.64	22.73	24.30
248	1,499,944	22.00	22.48	24.63
249	1,499,942	18.47	25.08	28.26
250	1,499,941	21.72	24.57	25.58
251	1,499,938	22.97	25.23	23.59
252	1,499,928	23.20	19.34	21.31
253	1,499,923	24.73	21.51	21.07
254	1,499,917	25.59	18.37	19.38
255	1,499,909	27.97	17.05	19.52
256	1,499,899	28.87	13.97	21.81
257	1,499,889	26.03	20.54	16.05
258	1,499,882	24.96	21.51	19.88
259	1,499,869	27.80	14.65	21.30
260	1,499,850	28.43	20.10	16.31
261	1,499,827	24.47	13.81	19.46
262	1,499,809	28.87	13.96	19.01
263	1,499,783	31.03	16.74	14.65

base position	coverage	% Adenine	% Thymine	% Cytosine
264	1,499,768	27.45	17.03	13.75
265	1,499,746	28.85	19.98	15.51
266	1,499,719	27.02	15.55	14.79
267	1,499,694	29.95	18.89	13.34
268	1,499,673	31.22	17.70	16.84
269	1,499,653	29.25	20.34	15.31
270	1,499,631	25.35	19.62	14.74
271	1,499,614	26.56	18.50	20.34
272	1,499,217	25.22	17.55	23.81
273	1,498,771	25.62	21.16	24.92
274	1,497,253	26.73	28.90	16.42
275	1,435,024	23.41	25.63	21.31
276	1,053,560	0.00	34.44	24.65

% Guanine	% Ambiguous
23.24	0.03
21.94	0.00
21.78	0.00
21.10	0.00
26.00	0.00
37.86	0.00
37.95	0.00
40.76	0.00
34.34	0.00
47.18	0.00
44.64	0.00
34.50	0.00
31.17	0.00
42.12	0.00
32.89	0.00
33.07	0.00
41.49	0.00
29.31	0.01
38.17	0.00
35.15	0.00
37.72	0.00
35.32	0.00

% Guanine	% Ambiguous
24.26	0.00
22.61	0.00
23.83	0.00
20.53	0.00
19.95	0.00
21.97	0.00
30.99	0.01
28.50	0.00
24.17	0.00
23.22	0.00
19.27	0.01
21.36	0.00
21.80	0.00
34.58	0.00
31.93	0.00
23.37	0.00
27.58	0.00
32.13	0.00
23.85	0.00
20.78	0.00
21.55	0.00
29.01	0.00
25.57	0.01
26.85	0.00
25.61	0.00
38.27	0.00
23.97	0.00
24.59	0.00
29.11	0.01
29.19	0.00
25.58	0.01
38.64	0.00
28.29	0.00
30.30	0.00
27.90	0.00
25.39	0.00
34.23	0.00

% Guanine	% Ambiguous
24.79	0.00
28.31	0.00
39.34	0.00
28.55	0.00
38.99	0.00
28.08	0.00
39.72	0.00
28.47	0.00
36.02	0.00
29.16	0.00
38.11	0.00
25.41	0.01
24.99	0.00
35.38	0.00
24.29	0.00
25.87	0.00
38.84	0.00
36.99	0.01
25.47	0.00
24.33	0.00
22.90	0.03
23.66	0.00
24.98	0.00
35.13	0.01
35.54	0.00
29.96	0.01
28.62	0.00
25.25	0.02
33.33	0.00
27.20	0.00
26.54	0.00
25.85	0.00
28.24	0.00
29.02	0.00
26.12	0.00
28.82	0.00
28.29	0.00

% Guanine	% Ambiguous
24.31	0.00
28.37	0.00
24.71	0.00
24.91	0.00
29.05	0.00
30.05	0.00
26.93	0.00
28.43	0.00
36.14	0.00
37.26	0.00
39.34	0.00
29.43	0.00
29.86	0.00
34.12	0.00
26.03	0.00
25.42	0.00
25.95	0.00
24.87	0.00
26.95	0.00
25.37	0.00
26.86	0.00
28.89	0.00
32.93	0.00
30.98	0.00
29.33	0.00
29.70	0.00
29.21	0.00
28.04	0.00
23.50	0.00
27.22	0.00
22.26	0.00
22.00	0.00
20.60	0.00
23.27	0.00
22.97	0.00
26.07	0.00
22.18	0.00

% Guanine	% Ambiguous
23.75	0.00
28.08	0.00
27.92	0.00
28.01	0.00
28.67	0.00
29.76	0.00
30.49	0.00
31.50	0.00
29.55	0.00
32.53	0.00
36.05	0.00
32.73	0.00
29.23	0.01
30.98	0.02
31.42	0.01
31.27	0.00
28.77	0.00
27.15	0.01
27.05	0.01
30.57	0.01
26.81	0.00
34.33	0.00
36.07	0.00
30.51	0.00
31.08	0.00
37.48	0.00
41.11	0.00
38.25	0.01
39.76	0.01
34.84	0.01
36.38	0.00
42.60	0.00
37.44	0.00
35.74	0.00
34.04	0.00
29.88	0.00
29.01	0.00

% Guanine	% Ambiguous
26.23	0.00
28.42	0.00
23.39	0.00
23.90	0.00
31.75	0.00
29.17	0.00
29.85	0.00
25.52	0.00
31.36	0.00
25.09	0.00
29.44	0.00
31.02	0.00
26.91	0.00
35.31	0.00
30.08	0.00
29.59	0.00
28.45	0.00
28.42	0.00
28.09	0.00
29.72	0.00
27.89	0.00
31.32	0.00
29.33	0.00
25.15	0.00
28.03	0.00
29.47	0.00
29.55	0.00
23.78	0.00
24.21	0.00
29.94	0.00
33.09	0.00
29.52	0.00
31.25	0.00
32.05	0.00
34.03	0.00
30.42	0.00
28.12	0.00

% Guanine	% Ambiguous
33.09	0.00
31.08	0.00
35.61	0.00
31.41	0.00
32.78	0.00
37.25	0.00
30.07	0.00
29.32	0.00
28.17	0.00
30.06	0.00
31.08	0.00
30.03	0.00
29.50	0.00
29.30	0.00
31.73	0.00
31.72	0.00
26.45	0.00
29.48	0.00
35.20	0.00
33.02	0.00
31.86	0.00
26.59	0.00
35.14	0.00
28.95	0.00
29.96	0.00
26.89	0.00
30.10	0.00
34.95	0.00
27.86	0.00
28.08	0.01
27.50	0.00
29.35	0.00
26.20	0.00
29.78	0.00
24.46	0.00
21.99	0.00
27.65	0.00

% Guanine	% Ambiguous
24.78	0.00
23.44	0.00
30.33	0.00
30.89	0.00
28.19	0.00
28.12	0.00
28.21	0.00
36.15	0.00
32.69	0.00
36.65	0.00
35.46	0.00
35.35	0.00
37.37	0.00
33.65	0.00
36.26	0.00
35.15	0.00
42.26	0.00
38.16	0.00
37.57	0.00
41.77	0.00
35.65	0.00
42.65	0.00
37.82	0.00
34.24	0.01
35.11	0.00
33.87	6.43
34.60	0.00
33.43	0.00
28.31	0.00
27.95	0.00
29.65	0.00
40.91	0.00

R2

Summarizes the coverages for the four DNA nucleotides and ambiguous bases for R2 reads.
coverage: total number of bases observed at that base position

% ACTGN: number of nucleotides observed per type and base position normalized to the total number of nucleotides observed at that position

base position	coverage	% Adenine	% Thymine	% Cytosine
1	1,514,316	20.70	22.92	25.21
2	1,514,316	34.63	27.76	18.91
3	1,514,316	21.99	25.55	30.57
4	1,514,316	21.80	36.35	20.48
5	1,514,316	33.19	21.61	26.07
6	1,514,316	17.93	24.32	40.56
7	1,514,316	16.87	31.42	28.57
8	1,514,316	19.58	25.45	32.81
9	1,514,316	13.34	23.80	31.31
10	1,514,316	10.11	20.16	36.18
11	1,514,316	8.70	16.67	41.15
12	1,514,316	5.50	33.23	41.13
13	1,514,316	17.77	21.91	36.37
14	1,514,316	6.06	35.21	34.22
15	1,514,316	8.33	28.86	46.04
16	1,514,316	8.84	43.01	29.48
17	1,514,316	21.22	28.14	25.74
18	1,514,316	20.66	27.41	31.04
19	1,514,316	13.31	42.12	25.36
20	1,514,316	12.63	28.83	37.98
21	1,514,316	12.05	26.40	36.34
22	1,514,316	12.84	41.98	21.07
23	1,514,316	20.74	27.11	19.59
24	1,514,316	22.59	36.66	20.01
25	1,514,316	18.33	38.26	22.21
26	1,514,316	16.82	38.83	25.29
27	1,514,316	19.85	31.14	21.16
28	1,514,316	20.23	30.40	26.53
29	1,514,316	14.72	43.71	24.72
30	1,514,316	19.98	28.87	34.89
31	1,514,316	15.44	26.50	37.08
32	1,514,316	14.99	24.08	37.88
33	1,514,316	13.10	23.99	39.29
34	1,514,316	17.33	23.90	31.89

base position	coverage	% Adenine	% Thymine	% Cytosine
35	1,514,316	13.72	20.44	42.01
36	1,514,316	14.90	23.23	28.87
37	1,514,315	11.67	30.00	35.06
38	1,514,315	13.53	38.79	24.47
39	1,514,315	13.07	39.28	28.18
40	1,514,314	11.32	44.28	30.25
41	1,514,314	12.47	30.47	43.76
42	1,514,310	15.30	30.46	29.90
43	1,514,284	17.99	34.48	36.30
44	1,514,266	18.33	34.68	29.33
45	1,514,252	17.58	32.00	38.19
46	1,514,226	15.52	34.44	32.85
47	1,514,190	16.82	36.58	24.50
48	1,514,148	16.33	25.56	34.85
49	1,514,107	32.90	24.60	20.08
50	1,514,081	19.73	18.42	24.13
51	1,514,050	17.79	18.20	36.30
52	1,514,008	14.34	18.04	25.45
53	1,513,983	14.92	31.09	25.26
54	1,513,956	17.39	17.66	40.82
55	1,513,938	30.57	15.58	31.17
56	1,513,926	20.97	14.32	32.83
57	1,513,923	17.58	31.67	33.68
58	1,513,916	20.81	32.79	31.68
59	1,513,914	24.97	28.25	29.94
60	1,513,914	24.53	27.52	39.01
61	1,513,912	26.21	30.99	29.68
62	1,513,911	23.57	30.25	26.93
63	1,513,911	24.35	36.96	24.64
64	1,513,911	18.47	32.35	35.41
65	1,513,911	18.59	32.34	34.30
66	1,513,907	27.91	31.15	25.01
67	1,513,904	18.84	27.69	22.82
68	1,513,903	20.72	32.12	24.93
69	1,513,902	19.16	28.92	25.97
70	1,513,899	26.42	26.55	26.20
71	1,513,897	17.90	24.71	28.13

base position	coverage	% Adenine	% Thymine	% Cytosine
72	1,513,897	16.78	28.24	36.06
73	1,513,896	18.26	32.16	31.72
74	1,513,895	25.13	21.99	27.07
75	1,513,895	24.54	22.91	37.42
76	1,513,895	25.60	22.55	37.55
77	1,513,895	26.42	30.91	28.64
78	1,513,895	28.47	31.54	25.62
79	1,513,894	29.43	20.31	33.21
80	1,513,894	32.27	20.80	21.07
81	1,513,894	25.23	24.73	29.83
82	1,513,894	22.92	23.06	33.24
83	1,513,894	30.34	25.43	22.51
84	1,513,894	18.29	34.81	24.01
85	1,513,894	15.51	29.99	29.66
86	1,513,894	14.90	27.26	22.11
87	1,513,894	13.54	27.60	20.89
88	1,513,894	13.56	35.45	27.09
89	1,513,894	16.52	27.01	24.84
90	1,513,894	14.48	36.87	25.81
91	1,513,894	14.57	37.31	24.63
92	1,513,894	17.08	25.39	35.24
93	1,513,894	19.41	31.10	28.16
94	1,513,894	20.81	31.40	26.54
95	1,513,894	25.88	18.86	36.13
96	1,513,894	26.58	19.14	34.87
97	1,513,894	31.01	23.81	29.08
98	1,513,894	27.47	21.61	31.25
99	1,513,894	35.14	19.31	27.15
100	1,513,894	22.60	30.93	28.35
101	1,513,894	30.33	21.18	31.88
102	1,513,893	20.76	35.08	27.21
103	1,513,892	19.36	24.78	38.95
104	1,513,892	19.18	36.73	29.92
105	1,513,891	31.91	22.73	29.53
106	1,513,890	25.03	20.78	40.03
107	1,513,890	23.03	18.98	34.20
108	1,513,890	26.08	20.49	40.88

base position	coverage	% Adenine	% Thymine	% Cytosine
109	1,513,890	37.02	20.06	31.65
110	1,513,890	26.75	29.48	28.36
111	1,513,890	25.72	26.34	31.99
112	1,513,890	24.91	25.05	33.94
113	1,513,889	26.10	14.65	41.51
114	1,513,889	39.34	14.03	28.18
115	1,513,889	26.13	14.85	39.05
116	1,513,879	24.06	17.46	38.72
117	1,513,865	22.17	22.49	25.70
118	1,513,837	21.79	18.83	36.63
119	1,513,779	22.45	28.90	26.17
120	1,513,739	31.91	21.46	26.78
121	1,513,677	19.45	22.43	36.92
122	1,513,606	26.71	21.65	28.79
123	1,513,534	18.80	18.21	39.31
124	1,513,438	21.21	21.94	30.41
125	1,513,308	24.25	22.68	27.97
126	1,513,195	20.50	15.90	30.15
127	1,513,069	21.19	17.70	27.64
128	1,512,948	32.44	15.68	28.18
129	1,512,829	27.29	16.75	28.60
130	1,512,736	23.31	28.69	26.82
131	1,512,663	20.49	29.10	29.78
132	1,512,583	19.89	16.96	40.75
133	1,512,524	18.08	18.57	42.00
134	1,512,467	26.08	19.49	29.11
135	1,512,432	18.37	22.34	37.86
136	1,512,405	17.44	26.26	36.31
137	1,512,395	18.99	24.78	36.03
138	1,512,384	25.17	25.30	28.11
139	1,512,378	17.29	24.27	35.38
140	1,512,368	18.07	23.27	35.06
141	1,512,363	17.50	34.63	28.34
142	1,512,357	19.80	31.40	31.22
143	1,512,350	20.26	33.99	27.65
144	1,512,347	20.76	28.37	33.71
145	1,512,341	22.10	22.75	35.29

base position	coverage	% Adenine	% Thymine	% Cytosine
146	1,512,341	23.66	29.01	31.82
147	1,512,339	20.77	25.70	29.54
148	1,512,338	21.60	29.76	34.83
149	1,512,336	32.23	24.10	29.12
150	1,512,336	21.74	23.64	40.54
151	1,512,336	19.16	35.88	30.21
152	1,512,336	17.72	27.90	41.14
153	1,512,316	22.06	33.52	31.00
154	1,512,315	25.71	29.60	31.36
155	1,512,315	16.43	26.95	31.60
156	1,512,315	15.23	34.98	34.90
157	1,512,312	15.24	38.38	34.27
158	1,512,312	14.24	34.42	36.37
159	1,512,307	20.29	29.46	31.99
160	1,512,299	22.28	28.23	36.30
161	1,512,284	14.23	31.06	40.32
162	1,512,252	26.66	26.89	31.41
163	1,512,196	16.03	25.52	30.35
164	1,512,131	15.92	39.08	28.98
165	1,512,041	19.80	34.00	28.96
166	1,511,915	17.04	34.25	31.37
167	1,511,761	18.24	27.87	33.43
168	1,511,581	18.74	25.04	33.81
169	1,511,370	22.09	25.58	27.09
170	1,511,138	29.58	19.89	28.73
171	1,510,869	23.18	26.16	26.61
172	1,510,568	23.10	19.86	23.78
173	1,510,248	24.85	19.60	35.12
174	1,509,928	33.00	21.54	25.82
175	1,509,648	30.62	22.07	25.27
176	1,509,392	23.87	27.61	25.35
177	1,509,150	25.66	28.57	29.05
178	1,508,958	23.53	24.53	33.36
179	1,508,795	23.43	28.86	26.31
180	1,508,668	25.67	25.88	24.44
181	1,508,525	21.13	30.54	28.71
182	1,508,456	18.53	29.15	26.87

base position	coverage	% Adenine	% Thymine	% Cytosine
183	1,508,397	16.66	30.30	24.63
184	1,508,353	15.14	39.44	25.73
185	1,508,340	15.58	36.99	26.76
186	1,508,328	18.04	30.69	26.15
187	1,508,325	24.87	29.08	27.08
188	1,508,319	14.94	29.62	29.06
189	1,508,318	15.61	28.38	38.24
190	1,508,312	18.66	28.71	32.08
191	1,508,247	23.49	28.51	26.08
192	1,508,179	22.94	28.24	28.14
193	1,508,136	24.93	28.48	23.15
194	1,507,653	24.04	23.31	23.72
195	1,507,170	22.77	23.37	25.02
196	1,506,852	22.68	28.70	25.53
197	1,506,588	20.80	36.84	20.37
198	1,506,033	18.63	38.24	22.53
199	1,505,459	19.77	35.51	23.33
200	1,505,098	19.63	25.06	32.73
201	1,504,758	26.78	26.17	23.83
202	1,504,658	16.66	27.28	32.22
203	1,504,531	20.72	28.33	29.56
204	1,504,428	15.81	33.78	30.25
205	1,504,355	14.31	29.39	33.08
206	1,504,313	20.14	25.58	28.41
207	1,504,312	19.35	24.53	28.42
208	1,504,312	21.95	24.04	31.36
209	1,504,309	14.45	25.55	39.66
210	1,504,299	14.61	36.90	27.58
211	1,504,040	15.65	34.11	29.06
212	1,503,814	21.08	25.74	29.02
213	1,503,685	20.03	29.10	32.05
214	1,503,553	19.51	28.08	34.02
215	1,503,283	20.65	30.31	28.93
216	1,502,687	23.54	29.26	29.68
217	1,502,227	23.18	26.32	31.50
218	1,501,838	18.17	25.31	37.31
219	1,501,660	19.07	25.94	31.26

base position	coverage	% Adenine	% Thymine	% Cytosine
220	1,501,306	18.31	27.65	27.22
221	1,500,920	17.48	25.83	38.99
222	1,500,675	17.40	27.38	34.06
223	1,500,474	18.33	32.62	22.19
224	1,500,473	22.78	22.74	25.11
225	1,500,469	14.55	23.43	32.75
226	1,500,465	21.64	24.80	27.30
227	1,500,465	18.27	28.02	29.31
228	1,500,464	16.69	26.42	28.33
229	1,500,460	15.29	26.09	35.83
230	1,500,457	15.61	28.93	33.51
231	1,500,452	16.02	31.13	34.77
232	1,500,441	14.70	40.29	27.07
233	1,500,431	16.37	40.61	25.87
234	1,500,422	20.36	39.30	24.22
235	1,500,412	28.57	29.50	26.24
236	1,500,405	23.45	28.95	28.93
237	1,500,393	20.32	27.49	28.33
238	1,500,381	18.41	25.55	39.93
239	1,500,367	19.44	24.61	40.33
240	1,500,360	22.41	24.18	37.37
241	1,500,352	30.40	23.31	29.10
242	1,500,351	29.55	25.76	28.81
243	1,500,344	22.28	32.06	30.84
244	1,500,340	32.96	22.72	30.61
245	1,500,336	31.24	23.39	29.19
246	1,500,332	24.64	29.77	27.02
247	1,500,327	20.58	31.82	30.56
248	1,500,327	20.87	24.12	39.60
249	1,500,326	20.22	24.84	37.89
250	1,500,324	19.12	23.24	28.38
251	1,500,324	20.02	24.36	28.36
252	1,500,321	27.65	27.60	29.55
253	1,500,310	19.46	32.55	33.83
254	1,500,308	27.01	24.60	34.34
255	1,500,301	23.80	23.73	37.10
256	1,500,291	14.06	24.60	45.86

base position	coverage	% Adenine	% Thymine	% Cytosine
257	1,500,280	15.42	25.33	37.35
258	1,500,271	14.30	24.90	46.29
259	1,500,264	12.79	29.89	41.31
260	1,500,249	11.74	30.75	40.65
261	1,500,232	12.76	25.95	36.95
262	1,500,210	13.34	24.13	42.55
263	1,500,194	15.94	23.53	38.43
264	1,500,170	16.71	22.20	39.31
265	1,500,157	11.90	22.77	43.03
266	1,500,135	14.77	28.22	34.95
267	1,500,110	13.34	35.17	29.00
268	1,500,089	19.70	25.01	32.33
269	1,500,071	15.33	22.02	39.83
270	1,500,052	15.25	24.16	31.07
271	1,500,023	18.90	32.06	29.79
272	1,499,962	25.69	26.90	28.90
273	1,499,647	19.24	34.04	28.70
274	1,495,543	24.05	31.07	28.36
275	1,444,002	28.68	21.22	34.80
276	1,241,911	0.00	31.07	61.10

% Guanine	% Ambiguous
31.10	0.07
18.70	0.00
21.89	0.00
21.38	0.00
19.13	0.00
17.19	0.00
23.14	0.00
22.15	0.00
31.55	0.00
33.55	0.00
33.48	0.00
20.13	0.00
23.95	0.00
24.51	0.00
16.78	0.00

% Guanine	% Ambiguous
18.68	0.00
24.90	0.00
20.89	0.00
19.21	0.00
20.56	0.00
25.21	0.00
24.11	0.00
32.56	0.00
20.73	0.00
21.19	0.00
19.06	0.00
27.84	0.00
22.84	0.00
16.85	0.00
16.26	0.00
20.98	0.00
23.06	0.00
23.62	0.00
26.88	0.00
23.82	0.00
32.99	0.00
23.27	0.00
23.20	0.00
19.46	0.00
14.15	0.01
13.30	0.00
24.34	0.00
11.22	0.00
17.66	0.00
12.22	0.00
17.19	0.00
22.10	0.00
23.26	0.00
22.41	0.01
37.71	0.00
27.70	0.00
42.16	0.00

% Guanine	% Ambiguous
28.73	0.00
24.12	0.01
22.68	0.00
31.87	0.01
17.06	0.00
14.71	0.00
16.84	0.00
8.94	0.00
13.12	0.00
19.25	0.00
14.05	0.00
13.77	0.00
14.77	0.00
15.93	0.00
30.65	0.00
22.24	0.00
25.94	0.00
20.84	0.00
29.26	0.00
18.93	0.00
17.86	0.00
25.81	0.00
15.12	0.00
14.29	0.00
14.02	0.00
14.37	0.00
17.05	0.00
25.86	0.00
20.21	0.00
20.78	0.00
21.73	0.00
22.89	0.00
20.63	4.23
35.71	0.00
37.96	0.00
23.90	0.00
31.62	0.01

% Guanine	% Ambiguous
22.84	0.00
23.48	0.00
22.29	0.00
21.32	0.01
21.24	0.00
19.14	0.00
19.41	0.00
16.09	0.00
19.67	0.00
18.40	0.00
18.11	0.00
16.62	0.00
16.95	0.00
16.90	0.01
14.17	0.00
15.83	0.00
14.16	0.00
23.78	0.01
12.55	0.00
11.27	0.00
15.41	0.00
15.95	0.00
16.10	0.00
17.73	0.00
18.45	0.01
19.97	0.00
19.75	0.00
29.63	0.00
22.74	0.00
22.48	0.00
19.85	0.00
21.20	0.00
22.84	0.00
23.68	0.00
26.44	0.00
25.09	0.01
33.45	0.00

% Guanine	% Ambiguous
33.47	0.00
23.71	0.00
27.36	0.00
21.17	0.01
20.64	0.00
22.40	0.00
21.34	0.00
25.32	0.00
21.44	0.00
19.99	0.00
20.19	0.00
21.42	0.00
23.06	0.00
23.60	0.00
19.53	0.00
17.57	0.00
18.09	0.00
17.15	0.00
19.85	0.00
15.51	0.00
24.00	0.00
13.80	0.00
14.53	0.01
14.07	0.00
14.74	0.00
13.25	0.00
13.42	0.00
13.33	0.00
25.01	0.01
14.89	0.01
12.10	0.00
14.97	0.00
18.25	0.01
13.19	0.00
14.39	0.01
15.04	0.01
28.05	0.04

% Guanine	% Ambiguous
16.01	0.01
17.24	0.01
17.33	0.01
20.45	0.01
22.40	0.01
25.23	0.01
21.80	0.01
24.05	0.00
33.26	0.00
20.44	0.00
19.64	0.01
22.04	0.00
23.16	0.00
16.71	0.00
18.58	0.00
21.39	0.00
24.00	0.00
19.62	0.00
25.45	0.00
28.40	0.00
19.69	0.00
20.67	0.00
25.11	0.00
18.97	0.00
26.38	0.00
17.77	0.00
20.55	0.00
21.92	0.00
20.67	0.00
23.44	0.01
28.93	0.00
28.84	0.00
23.08	0.00
21.99	0.00
20.59	0.00
21.38	0.00
22.57	0.00

% Guanine	% Ambiguous
23.22	0.00
23.83	0.00
21.38	0.01
20.15	0.01
23.22	0.00
25.87	0.00
27.70	0.00
22.65	0.00
20.33	0.00
20.91	0.00
21.18	0.00
24.13	0.03
18.81	0.00
18.38	0.01
20.11	0.00
17.51	0.01
19.00	0.00
19.19	0.01
23.73	0.00
26.82	0.00
17.69	0.00
21.16	0.00
26.86	0.00
29.36	0.00
29.27	0.00
26.26	0.00
24.39	0.01
28.55	0.00
22.79	0.00
21.95	0.00
18.08	0.00
17.94	0.00
17.15	0.00
16.12	0.00
15.68	0.00
18.67	0.00
23.86	0.00

% Guanine	% Ambiguous
16.11	0.00
15.62	0.00
16.04	0.00
17.18	0.00
15.88	0.00
14.82	0.00
13.70	0.00
16.18	0.00
18.57	0.00
17.04	0.00
15.41	0.00
17.05	0.00
29.26	0.00
27.26	0.00
15.19	0.01
14.16	0.00
14.05	0.00
15.36	0.01
15.48	0.00
21.90	0.01
14.51	0.00
16.01	0.00
16.86	0.00
24.34	0.00
19.98	0.00
22.09	0.00
21.77	0.01
22.30	0.00
22.05	0.00
22.49	0.00
22.95	0.00
22.81	0.00
29.51	0.00
19.24	0.01
18.51	0.00
18.02	0.00
16.52	0.00

% Guanine	% Ambiguous
15.31	0.00
7.83	0.00

3.3 GC-content

Summarizes the cumulative coverage of G- and C-bases.
coverage: total number of bases observed at that base position
abs: number of G- and C-bases observed at current position
%: number of G- and C-bases observed at current position normalized to the total number of bases observed at that position in R1 / R2

base position	R1: abs	R1: %	R2: abs	R2: %
1	858,986	56.72	852,792	56.32
2	874,423	57.74	569,484	37.61
3	666,021	43.98	794,417	52.46
4	634,034	41.87	633,806	41.85
5	902,780	59.62	684,457	45.20
6	844,763	55.79	874,554	57.75
7	907,600	59.93	783,015	51.71
8	914,424	60.39	832,302	54.96
9	852,543	56.30	951,891	62.86
10	943,102	62.28	1,055,983	69.73
11	963,329	63.61	1,130,075	74.63
12	1,006,611	66.47	927,769	61.27
13	800,785	52.88	913,410	60.32
14	999,230	65.99	889,400	58.73
15	996,424	65.80	951,241	62.82
16	828,001	54.68	729,252	48.16
17	943,198	62.29	766,775	50.64
18	780,988	51.57	786,408	51.93
19	872,584	57.62	674,886	44.57
20	860,749	56.84	886,433	58.54
21	890,339	58.79	932,027	61.55
22	875,342	57.80	684,175	45.18
23	697,197	46.04	789,716	52.15
24	638,480	42.16	617,012	40.75
25	645,690	42.64	657,287	43.40
26	665,689	43.96	671,568	44.35

base position	R1: abs	R1: %	R2: abs	R2: %
27	608,449	40.18	741,991	49.00
28	619,223	40.89	747,604	49.37
29	888,271	58.66	629,492	41.57
30	877,428	57.94	774,642	51.15
31	736,717	48.65	879,160	58.06
32	840,363	55.49	922,716	60.93
33	653,173	43.13	952,591	62.91
34	712,774	47.07	889,924	58.77
35	755,243	49.87	996,955	65.84
36	940,960	62.14	936,830	61.86
37	891,921	58.90	883,338	58.33
38	733,344	48.43	721,867	47.67
39	847,137	55.94	721,467	47.64
40	842,965	55.67	672,234	44.39
41	721,899	47.67	864,152	57.07
42	643,370	42.49	821,323	54.24
43	664,291	43.87	719,673	47.53
44	863,742	57.04	711,536	46.99
45	698,968	46.16	763,382	50.41
46	805,806	53.22	757,772	50.04
47	611,500	40.39	705,521	46.59
48	796,532	52.61	879,786	58.10
49	588,351	38.86	643,401	42.49
50	655,504	43.29	936,342	61.84
51	790,463	52.21	969,042	64.00
52	783,809	51.77	1,023,653	67.61
53	625,417	41.31	817,412	53.99
54	802,863	53.03	983,139	64.94
55	839,665	55.46	815,310	53.85
56	749,034	49.48	979,513	64.70
57	665,686	43.97	768,193	50.74
58	726,783	48.01	702,349	46.39
59	740,289	48.90	708,255	46.78
60	743,241	49.09	725,879	47.95
61	757,983	50.07	647,932	42.80
62	848,747	56.06	699,162	46.18
63	788,281	52.07	585,735	38.69

base position	R1: abs	R1: %	R2: abs	R2: %
64	844,979	55.81	744,598	49.18
65	663,595	43.83	742,799	49.06
66	859,020	56.74	619,826	40.94
67	791,315	52.27	809,500	53.47
68	812,272	53.65	714,041	47.17
69	730,036	48.22	785,917	51.91
70	848,366	56.04	712,100	47.04
71	658,564	43.50	868,880	57.39
72	650,704	42.98	832,482	54.99
73	821,159	54.24	750,608	49.58
74	658,308	43.48	800,480	52.88
75	709,683	46.88	795,511	52.55
76	854,300	56.43	784,835	51.84
77	827,621	54.67	645,864	42.66
78	703,006	46.44	605,474	39.99
79	668,324	44.15	760,855	50.26
80	618,675	40.87	710,371	46.92
81	589,357	38.93	757,533	50.04
82	694,484	45.87	817,885	54.03
83	756,647	49.98	669,664	44.23
84	767,665	50.71	710,010	46.90
85	690,939	45.64	761,207	50.28
86	677,547	44.76	875,432	57.83
87	629,772	41.60	890,981	58.85
88	681,267	45.00	771,899	50.99
89	593,517	39.20	854,739	56.46
90	578,814	38.23	736,520	48.65
91	591,586	39.08	728,434	48.12
92	645,655	42.65	870,995	57.53
93	721,965	47.69	749,100	49.48
94	835,052	55.16	723,427	47.79
95	701,346	46.33	836,676	55.27
96	840,966	55.55	821,633	54.27
97	702,478	46.40	683,907	45.18
98	753,238	49.76	770,832	50.92
99	692,688	45.76	689,580	45.55
100	734,937	48.55	703,397	46.46

base position	R1: abs	R1: %	R2: abs	R2: %
101	799,882	52.84	734,158	48.49
102	809,063	53.44	668,489	44.16
103	754,858	49.86	845,573	55.85
104	759,350	50.16	667,436	44.09
105	853,128	56.35	686,702	45.36
106	852,524	56.31	820,420	54.19
107	868,328	57.36	877,701	57.98
108	713,766	47.15	808,909	53.43
109	728,956	48.15	649,695	42.92
110	802,512	53.01	662,646	43.77
111	665,628	43.97	725,744	47.94
112	688,543	45.48	757,552	50.04
113	699,835	46.23	896,804	59.24
114	676,193	44.67	705,883	46.63
115	721,773	47.68	893,530	59.02
116	699,581	46.21	885,195	58.47
117	700,213	46.25	837,739	55.34
118	777,817	51.38	898,834	59.37
119	807,952	53.37	736,436	48.65
120	780,381	51.56	705,880	46.63
121	809,477	53.48	879,767	58.12
122	753,715	49.80	781,469	51.63
123	804,143	53.13	953,349	62.99
124	766,004	50.62	860,403	56.85
125	690,648	45.64	802,931	53.06
126	788,687	52.13	962,332	63.60
127	746,334	49.33	924,577	61.11
128	709,706	46.91	784,975	51.88
129	694,300	45.90	846,680	55.97
130	752,181	49.73	726,053	48.00
131	708,633	46.85	762,573	50.41
132	733,086	48.47	955,097	63.14
133	649,395	42.94	958,071	63.34
134	664,491	43.94	823,234	54.43
135	698,848	46.21	896,803	59.30
136	710,388	46.97	851,429	56.30
137	753,877	49.85	850,348	56.23

base position	R1: abs	R1: %	R2: abs	R2: %
138	728,454	48.17	749,042	49.53
139	795,931	52.63	883,774	58.44
140	754,743	49.90	887,150	58.66
141	757,913	50.11	723,962	47.87
142	791,169	52.31	738,006	48.80
143	867,009	57.33	691,872	45.75
144	874,590	57.83	769,220	50.86
145	861,991	57.00	833,942	55.14
146	774,417	51.21	715,692	47.32
147	792,413	52.40	809,567	53.53
148	846,758	55.99	735,525	48.63
149	873,445	57.75	660,170	43.65
150	802,340	53.05	825,862	54.61
151	737,936	48.79	679,867	44.95
152	769,996	50.92	822,509	54.39
153	819,317	54.18	671,741	44.42
154	766,251	50.67	675,896	44.69
155	795,939	52.63	856,096	56.61
156	874,093	57.80	753,017	49.79
157	786,900	52.03	701,288	46.37
158	739,087	48.87	776,395	51.34
159	815,102	53.90	759,807	50.24
160	887,852	58.71	748,388	49.49
161	815,849	53.95	827,402	54.71
162	853,377	56.43	702,394	46.45
163	753,935	49.86	883,226	58.41
164	821,528	54.33	680,302	44.99
165	823,984	54.50	698,477	46.19
166	805,252	53.27	736,354	48.70
167	809,008	53.52	814,527	53.88
168	779,842	51.60	849,719	56.21
169	717,010	47.45	790,749	52.32
170	695,235	46.02	763,513	50.53
171	747,613	49.49	765,367	50.66
172	714,387	47.30	861,616	57.04
173	673,219	44.59	838,980	55.55
174	782,887	51.86	686,459	45.46

base position	R1: abs	R1: %	R2: abs	R2: %
175	771,253	51.10	714,162	47.31
176	728,748	48.29	732,180	48.51
177	791,870	52.48	690,645	45.76
178	754,459	50.00	783,685	51.94
179	839,935	55.67	719,777	47.71
180	727,179	48.21	730,863	48.44
181	765,314	50.74	729,024	48.33
182	807,570	53.54	789,180	52.32
183	764,812	50.71	799,980	53.04
184	833,219	55.24	684,956	45.41
185	803,573	53.28	715,417	47.43
186	792,257	52.53	773,244	51.26
187	774,557	51.35	694,582	46.05
188	763,697	50.63	836,177	55.44
189	683,160	45.29	844,857	56.01
190	727,067	48.21	793,886	52.63
191	735,171	48.75	723,967	48.00
192	738,056	48.94	736,167	48.81
193	774,819	51.39	702,544	46.58
194	706,297	46.86	793,821	52.65
195	690,725	45.84	811,741	53.86
196	761,919	50.58	732,593	48.62
197	734,382	48.77	638,113	42.35
198	658,762	43.76	649,465	43.12
199	673,594	44.76	673,183	44.72
200	768,090	51.05	832,382	55.30
201	775,442	51.54	707,951	47.05
202	720,516	47.90	843,348	56.05
203	741,926	49.32	766,486	50.95
204	789,149	52.46	758,314	50.41
205	824,236	54.80	847,026	56.30
206	714,407	47.50	816,475	54.28
207	735,656	48.91	844,236	56.12
208	763,419	50.76	812,447	54.01
209	785,290	52.21	902,545	60.00
210	837,353	55.68	729,354	48.48
211	806,130	53.61	755,598	50.24

base position	R1: abs	R1: %	R2: abs	R2: %
212	808,684	53.79	799,224	53.15
213	877,155	58.35	764,795	50.86
214	777,921	51.76	787,837	52.40
215	819,409	54.54	737,215	49.04
216	816,379	54.36	709,143	47.19
217	755,091	50.29	758,509	50.49
218	782,948	52.15	848,669	56.51
219	794,697	52.95	825,736	54.99
220	755,647	50.36	811,240	54.04
221	774,859	51.65	850,827	56.69
222	781,569	52.10	828,581	55.21
223	772,023	51.47	735,921	49.05
224	728,937	48.59	817,328	54.47
225	738,247	49.21	930,610	62.02
226	804,429	53.63	803,573	53.55
227	766,529	51.10	805,787	53.70
228	762,805	50.85	853,541	56.89
229	755,645	50.37	879,530	58.62
230	826,643	55.11	832,138	55.46
231	719,227	47.95	792,948	52.85
232	788,779	52.58	675,317	45.01
233	703,988	46.93	645,494	43.02
234	762,625	50.84	605,250	40.34
235	846,465	56.43	628,973	41.92
236	793,952	52.93	714,199	47.60
237	746,715	49.78	783,019	52.19
238	766,707	51.11	840,754	56.04
239	788,342	52.56	839,493	55.95
240	764,888	50.99	801,340	53.41
241	859,676	57.31	694,414	46.28
242	824,994	55.00	670,422	44.68
243	722,697	48.18	685,140	45.67
244	818,402	54.56	664,834	44.31
245	784,838	52.32	680,692	45.37
246	772,721	51.52	683,943	45.59
247	819,414	54.63	714,198	47.60
248	832,715	55.52	825,327	55.01

base position	R1: abs	R1: %	R2: abs	R2: %
249	846,760	56.45	824,290	54.94
250	805,554	53.71	864,716	57.64
251	776,923	51.80	834,491	55.62
252	861,880	57.46	671,254	44.74
253	806,286	53.76	720,034	47.99
254	840,442	56.03	725,938	48.39
255	824,613	54.98	787,006	52.46
256	857,358	57.16	920,234	61.34
257	801,258	53.42	888,821	59.24
258	802,963	53.54	912,113	60.80
259	863,272	57.56	859,817	57.31
260	771,943	51.47	862,796	57.51
261	925,665	61.72	919,540	61.29
262	857,498	57.17	938,028	62.53
263	783,272	52.23	907,939	60.52
264	832,660	55.52	916,300	61.08
265	767,302	51.16	980,063	65.33
266	861,381	57.44	855,159	57.01
267	767,191	51.16	772,384	51.49
268	766,009	51.08	829,298	55.28
269	756,054	50.42	939,689	62.64
270	728,904	48.61	908,844	60.59
271	823,865	54.94	735,461	49.03
272	858,059	57.23	711,087	47.41
273	797,746	53.23	700,628	46.72
274	664,325	44.37	671,183	44.88
275	731,260	50.96	723,575	50.11
276	690,727	65.56	855,986	68.92

3.4 Ambiguous base-content

Summarizes the cumulative coverage of ambiguous bases.
coverage: total number of bases observed at that base position
abs: number of ambiguous bases observed at current position

%: number of ambiguous bases observed at current position normalized to the total number of bases observed at that position in R1 / R2

base position	R1: abs	R1: %	R2: abs	R2: %
1	403	0.03	1,005	0.07
5	0	0.00	1	0.00
6	1	0.00	6	0.00
7	0	0.00	23	0.00
9	0	0.00	12	0.00
10	0	0.00	40	0.00
11	0	0.00	6	0.00
13	1	0.00	2	0.00
14	0	0.00	20	0.00
16	2	0.00	2	0.00
18	133	0.01	26	0.00
20	0	0.00	2	0.00
22	0	0.00	4	0.00
24	1	0.00	2	0.00
25	0	0.00	12	0.00
26	11	0.00	0	0.00
27	0	0.00	46	0.00
28	1	0.00	27	0.00
29	188	0.01	2	0.00
30	17	0.00	3	0.00
31	0	0.00	49	0.00
32	60	0.00	5	0.00
33	110	0.01	0	0.00
34	4	0.00	1	0.00
36	64	0.00	0	0.00
37	24	0.00	11	0.00
38	32	0.00	55	0.00
39	0	0.00	26	0.00
40	40	0.00	121	0.01
41	11	0.00	4	0.00
42	33	0.00	29	0.00
43	0	0.00	72	0.00
44	65	0.00	64	0.00
45	102	0.01	8	0.00

base position	R1: abs	R1: %	R2: abs	R2: %
46	32	0.00	8	0.00
47	50	0.00	7	0.00
48	32	0.00	75	0.00
49	17	0.00	77	0.01
50	44	0.00	36	0.00
51	92	0.01	0	0.00
52	49	0.00	37	0.00
53	85	0.01	5	0.00
54	3	0.00	102	0.01
55	34	0.00	0	0.00
56	28	0.00	120	0.01
57	2	0.00	2	0.00
58	4	0.00	5	0.00
59	0	0.00	30	0.00
60	1	0.00	29	0.00
61	54	0.00	49	0.00
62	29	0.00	18	0.00
63	30	0.00	8	0.00
64	1	0.00	4	0.00
65	46	0.00	0	0.00
66	3	0.00	0	0.00
67	59	0.00	2	0.00
68	58	0.00	4	0.00
69	61	0.00	15	0.00
70	16	0.00	0	0.00
71	130	0.01	1	0.00
72	34	0.00	3	0.00
73	56	0.00	12	0.00
74	5	0.00	9	0.00
75	0	0.00	42	0.00
76	2	0.00	60	0.00
77	85	0.01	25	0.00
78	9	0.00	8	0.00
79	1	0.00	17	0.00
80	468	0.03	16	0.00
81	0	0.00	25	0.00
82	25	0.00	16	0.00

base position	R1: abs	R1: %	R2: abs	R2: %
83	96	0.01	24	0.00
84	53	0.00	28	0.00
85	82	0.01	63,969	4.23
86	19	0.00	59	0.00
87	305	0.02	10	0.00
88	20	0.00	1	0.00
89	6	0.00	91	0.01
90	73	0.00	13	0.00
91	0	0.00	56	0.00
92	0	0.00	14	0.00
93	0	0.00	90	0.01
94	3	0.00	46	0.00
95	0	0.00	32	0.00
96	0	0.00	14	0.00
97	7	0.00	23	0.00
98	0	0.00	4	0.00
99	0	0.00	28	0.00
100	0	0.00	24	0.00
101	2	0.00	28	0.00
102	5	0.00	68	0.00
103	0	0.00	79	0.01
104	0	0.00	6	0.00
105	3	0.00	25	0.00
106	33	0.00	4	0.00
107	2	0.00	116	0.01
108	0	0.00	28	0.00
109	11	0.00	38	0.00
110	0	0.00	18	0.00
111	11	0.00	23	0.00
112	0	0.00	21	0.00
113	23	0.00	34	0.00
114	64	0.00	146	0.01
115	13	0.00	25	0.00
116	0	0.00	20	0.00
117	14	0.00	16	0.00
118	12	0.00	1	0.00
119	11	0.00	40	0.00

base position	R1: abs	R1: %	R2: abs	R2: %
120	49	0.00	0	0.00
121	5	0.00	50	0.00
122	0	0.00	9	0.00
123	8	0.00	0	0.00
124	17	0.00	43	0.00
125	11	0.00	115	0.01
126	7	0.00	44	0.00
127	17	0.00	31	0.00
128	0	0.00	30	0.00
130	3	0.00	98	0.01
131	17	0.00	35	0.00
132	35	0.00	63	0.00
133	0	0.00	31	0.00
134	8	0.00	74	0.00
135	1	0.00	13	0.00
136	2	0.00	47	0.00
137	3	0.00	59	0.00
138	1	0.00	43	0.00
139	2	0.00	38	0.00
140	0	0.00	34	0.00
141	13	0.00	28	0.00
142	6	0.00	58	0.00
143	23	0.00	18	0.00
144	39	0.00	41	0.00
145	65	0.00	56	0.00
146	112	0.01	43	0.00
147	227	0.02	40	0.00
148	108	0.01	72	0.00
149	21	0.00	135	0.01
150	9	0.00	41	0.00
151	85	0.01	10	0.00
152	208	0.01	9	0.00
153	113	0.01	41	0.00
154	7	0.00	53	0.00
155	2	0.00	136	0.01
156	0	0.00	79	0.01
157	4	0.00	61	0.00

base position	R1: abs	R1: %	R2: abs	R2: %
158	0	0.00	74	0.00
159	1	0.00	78	0.01
160	11	0.00	68	0.00
161	85	0.01	76	0.01
162	154	0.01	144	0.01
163	169	0.01	559	0.04
164	0	0.00	145	0.01
165	56	0.00	101	0.01
166	0	0.00	95	0.01
167	0	0.00	129	0.01
168	0	0.00	93	0.01
169	7	0.00	108	0.01
170	0	0.00	121	0.01
171	4	0.00	36	0.00
172	26	0.00	68	0.00
173	10	0.00	53	0.00
174	0	0.00	97	0.01
175	0	0.00	42	0.00
176	0	0.00	64	0.00
177	0	0.00	23	0.00
178	0	0.00	65	0.00
179	0	0.00	3	0.00
180	0	0.00	72	0.00
181	0	0.00	16	0.00
182	0	0.00	30	0.00
183	3	0.00	55	0.00
184	0	0.00	18	0.00
186	0	0.00	32	0.00
187	0	0.00	7	0.00
188	0	0.00	38	0.00
189	0	0.00	17	0.00
190	0	0.00	47	0.00
191	4	0.00	7	0.00
192	21	0.00	68	0.00
193	0	0.00	81	0.01
194	15	0.00	39	0.00
195	0	0.00	16	0.00

base position	R1: abs	R1: %	R2: abs	R2: %
196	0	0.00	46	0.00
197	0	0.00	34	0.00
198	7	0.00	54	0.00
199	0	0.00	43	0.00
200	0	0.00	39	0.00
201	42	0.00	34	0.00
202	29	0.00	53	0.00
203	0	0.00	111	0.01
204	29	0.00	129	0.01
205	0	0.00	34	0.00
206	0	0.00	8	0.00
208	0	0.00	22	0.00
209	1	0.00	28	0.00
210	0	0.00	44	0.00
211	0	0.00	67	0.00
212	1	0.00	522	0.03
213	0	0.00	71	0.00
214	15	0.00	118	0.01
215	1	0.00	4	0.00
216	13	0.00	92	0.01
217	14	0.00	0	0.00
218	0	0.00	76	0.01
219	9	0.00	42	0.00
220	43	0.00	50	0.00
221	0	0.00	30	0.00
222	0	0.00	71	0.00
224	0	0.00	59	0.00
225	1	0.00	17	0.00
226	5	0.00	68	0.00
227	0	0.00	90	0.01
228	39	0.00	39	0.00
230	0	0.00	57	0.00
231	0	0.00	24	0.00
232	0	0.00	45	0.00
233	0	0.00	52	0.00
234	0	0.00	55	0.00
235	0	0.00	63	0.00

base position	R1: abs	R1: %	R2: abs	R2: %
236	0	0.00	70	0.00
237	178	0.01	27	0.00
238	0	0.00	26	0.00
239	0	0.00	58	0.00
240	0	0.00	29	0.00
241	0	0.00	1	0.00
242	1	0.00	11	0.00
243	2	0.00	58	0.00
244	0	0.00	16	0.00
245	0	0.00	19	0.00
246	2	0.00	42	0.00
247	0	0.00	70	0.00
248	7	0.00	6	0.00
249	0	0.00	35	0.00
250	0	0.00	34	0.00
251	0	0.00	39	0.00
252	0	0.00	81	0.01
253	13	0.00	67	0.00
254	0	0.00	10	0.00
255	0	0.00	85	0.01
256	0	0.00	69	0.00
257	0	0.00	88	0.01
258	0	0.00	58	0.00
259	0	0.00	62	0.00
260	14	0.00	45	0.00
261	0	0.00	59	0.00
262	2	0.00	62	0.00
263	0	0.00	61	0.00
264	0	0.00	76	0.01
265	0	0.00	72	0.00
266	0	0.00	52	0.00
267	22	0.00	54	0.00
268	114	0.01	55	0.00
269	0	0.00	42	0.00
270	96,385	6.43	27	0.00
271	0	0.00	93	0.01
272	0	0.00	21	0.00

base position	R1: abs	R1: %	R2: abs	R2: %
273	0	0.00	16	0.00
274	0	0.00	25	0.00
275	0	0.00	1	0.00
276	2	0.00	47	0.00

3.5 Quality distribution

R1

Summarizes the base-quality distribution along the base positions for R1 reads.
coverage: total number of quality values observed at that base position
median & percentiles: median & percentiles of quality scores observed at that base position

base position	coverage	5%ile	25%ile	Median
1	1,514,316	33	34	34
2	1,514,316	34	34	34
3	1,514,316	34	34	34
4	1,514,316	34	34	34
5	1,514,316	34	34	34
6	1,514,316	36	38	38
7	1,514,316	36	38	38
8	1,514,316	35	38	38
9	1,514,316	35	38	38
10	1,514,316	36	38	38
11	1,514,316	35	38	38
12	1,514,316	35	38	38
13	1,514,316	34	38	38
14	1,514,316	34	38	38
15	1,514,316	34	38	38
16	1,514,316	34	38	38
17	1,514,316	35	38	38
18	1,514,316	34	38	38
19	1,514,316	35	38	38
20	1,514,316	35	38	38
21	1,514,316	35	38	38
22	1,514,316	35	38	38
23	1,514,316	35	38	38
24	1,514,316	35	38	38

base position	coverage	5%ile	25%ile	Median
25	1,514,316	34	38	38
26	1,514,316	34	38	38
27	1,514,316	34	38	38
28	1,514,316	34	38	38
29	1,514,316	34	38	38
30	1,514,316	34	38	38
31	1,514,316	34	38	38
32	1,514,316	34	38	38
33	1,514,316	34	38	38
34	1,514,316	34	38	38
35	1,514,316	34	38	38
36	1,514,315	34	38	38
37	1,514,315	34	38	38
38	1,514,314	34	38	38
39	1,514,313	34	38	38
40	1,514,313	34	38	38
41	1,514,309	34	38	38
42	1,514,283	34	38	38
43	1,514,265	34	38	38
44	1,514,251	34	38	38
45	1,514,224	34	38	38
46	1,514,188	34	38	38
47	1,514,145	34	38	38
48	1,514,105	34	38	38
49	1,514,078	34	38	38
50	1,514,046	34	38	38
51	1,514,005	34	38	38
52	1,513,981	34	38	38
53	1,513,954	34	38	38
54	1,513,936	34	38	38
55	1,513,924	34	38	38
56	1,513,921	34	38	38
57	1,513,914	34	38	38
58	1,513,912	34	38	38
59	1,513,912	34	38	38
60	1,513,910	34	38	38
61	1,513,909	34	38	38

base position	coverage	5%ile	25%ile	Median
62	1,513,909	34	38	38
63	1,513,909	34	38	38
64	1,513,909	32	38	38
65	1,513,905	34	38	38
66	1,513,902	34	38	38
67	1,513,901	34	38	38
68	1,513,900	34	38	38
69	1,513,897	34	38	38
70	1,513,895	34	38	38
71	1,513,895	34	38	38
72	1,513,894	34	38	38
73	1,513,894	34	38	38
74	1,513,894	34	38	38
75	1,513,894	34	38	38
76	1,513,894	34	38	38
77	1,513,894	34	38	38
78	1,513,894	34	38	38
79	1,513,894	34	38	38
80	1,513,894	33	38	38
81	1,513,894	34	38	38
82	1,513,894	34	38	38
83	1,513,894	34	38	38
84	1,513,894	34	38	38
85	1,513,894	34	38	38
86	1,513,894	33	38	38
87	1,513,894	33	38	38
88	1,513,894	34	38	38
89	1,513,894	33	38	38
90	1,513,894	34	38	38
91	1,513,894	34	38	38
92	1,513,894	34	38	38
93	1,513,894	34	38	38
94	1,513,894	33	38	38
95	1,513,894	33	38	38
96	1,513,894	33	38	38
97	1,513,894	32	38	38
98	1,513,894	32	38	38

base position	coverage	5%ile	25%ile	Median
99	1,513,894	33	38	38
100	1,513,893	32	38	38
101	1,513,893	34	38	38
102	1,513,893	32	38	38
103	1,513,892	32	38	38
104	1,513,891	31	38	38
105	1,513,890	33	38	38
106	1,513,890	32	38	38
107	1,513,890	34	38	38
108	1,513,890	33	38	38
109	1,513,890	34	38	38
110	1,513,890	33	38	38
111	1,513,890	33	38	38
112	1,513,889	33	38	38
113	1,513,889	33	38	38
114	1,513,889	34	38	38
115	1,513,878	34	38	38
116	1,513,863	32	38	38
117	1,513,832	33	38	38
118	1,513,778	33	38	38
119	1,513,736	32	38	38
120	1,513,675	33	38	38
121	1,513,604	33	38	38
122	1,513,528	32	38	38
123	1,513,433	32	38	38
124	1,513,305	31	38	38
125	1,513,191	31	38	38
126	1,513,066	31	38	38
127	1,512,948	31	38	38
128	1,512,829	31	38	38
129	1,512,735	30	38	38
130	1,512,662	31	38	38
131	1,512,583	31	38	38
132	1,512,521	31	38	38
133	1,512,465	30	38	38
134	1,512,427	30	38	38
135	1,512,402	30	38	38

base position	coverage	5%ile	25%ile	Median
136	1,512,392	30	38	38
137	1,512,381	31	38	38
138	1,512,374	30	38	38
139	1,512,365	30	38	38
140	1,512,360	30	38	38
141	1,512,355	31	38	38
142	1,512,347	31	38	38
143	1,512,344	30	38	38
144	1,512,338	30	38	38
145	1,512,338	31	38	38
146	1,512,336	30	38	38
147	1,512,335	30	38	38
148	1,512,334	31	38	38
149	1,512,334	30	38	38
150	1,512,334	30	38	38
151	1,512,334	30	38	38
152	1,512,315	29	38	38
153	1,512,314	29	38	38
154	1,512,314	29	38	38
155	1,512,314	29	38	38
156	1,512,311	30	38	38
157	1,512,311	30	38	38
158	1,512,306	28	37	38
159	1,512,298	29	37	38
160	1,512,285	29	37	38
161	1,512,252	29	38	38
162	1,512,196	29	38	38
163	1,512,129	29	38	38
164	1,512,037	29	38	38
165	1,511,907	29	38	38
166	1,511,752	29	38	38
167	1,511,572	29	38	38
168	1,511,357	27	37	38
169	1,511,128	28	37	38
170	1,510,856	27	37	38
171	1,510,558	27	37	38
172	1,510,240	27	37	38

base position	coverage	5%ile	25%ile	Median
173	1,509,917	27	37	38
174	1,509,635	27	37	38
175	1,509,379	28	37	38
176	1,509,135	27	37	38
177	1,508,940	27	37	38
178	1,508,776	27	37	38
179	1,508,652	27	37	38
180	1,508,504	27	37	38
181	1,508,433	27	37	38
182	1,508,374	27	37	38
183	1,508,329	27	37	38
184	1,508,316	27	37	38
185	1,508,303	27	37	38
186	1,508,300	26	37	38
187	1,508,296	26	37	38
188	1,508,294	26	37	38
189	1,508,287	27	37	38
190	1,508,219	26	37	38
191	1,508,148	25	37	38
192	1,508,098	26	37	38
193	1,507,595	27	37	38
194	1,507,108	25	37	38
195	1,506,762	26	37	38
196	1,506,483	25	37	38
197	1,505,911	25	37	38
198	1,505,326	25	37	38
199	1,504,936	25	37	38
200	1,504,585	25	37	38
201	1,504,483	25	37	38
202	1,504,350	25	37	38
203	1,504,245	25	37	38
204	1,504,166	25	37	38
205	1,504,119	24	37	38
206	1,504,118	25	37	38
207	1,504,118	25	37	38
208	1,504,116	25	37	38
209	1,504,100	24	37	38

base position	coverage	5%ile	25%ile	Median
210	1,503,837	25	37	38
211	1,503,602	25	37	38
212	1,503,457	25	37	38
213	1,503,321	25	37	38
214	1,503,041	23	37	38
215	1,502,419	24	37	38
216	1,501,919	23	37	38
217	1,501,494	24	37	38
218	1,501,313	24	37	38
219	1,500,944	24	37	38
220	1,500,544	23	37	38
221	1,500,270	23	37	38
222	1,500,062	23	37	38
223	1,500,061	23	37	38
224	1,500,059	23	36	38
225	1,500,058	23	36	38
226	1,500,058	23	37	38
227	1,500,056	23	37	38
228	1,500,052	23	37	38
229	1,500,052	23	37	38
230	1,500,048	23	37	38
231	1,500,037	23	37	38
232	1,500,028	23	36	38
233	1,500,017	23	36	38
234	1,500,008	23	36	38
235	1,500,001	23	36	38
236	1,499,997	22	36	38
237	1,499,994	22	36	38
238	1,499,985	22	36	38
239	1,499,979	23	36	38
240	1,499,971	22	36	38
241	1,499,968	22	36	38
242	1,499,960	21	36	38
243	1,499,956	21	36	38
244	1,499,953	21	36	38
245	1,499,949	21	36	38
246	1,499,945	20	36	38

base position	coverage	5%ile	25%ile	Median
247	1,499,945	20	36	38
248	1,499,944	20	36	38
249	1,499,942	20	35	38
250	1,499,941	18	34	37
251	1,499,938	18	35	37
252	1,499,928	18	35	38
253	1,499,923	18	34	38
254	1,499,917	18	34	38
255	1,499,909	18	35	38
256	1,499,899	19	35	38
257	1,499,889	19	34	38
258	1,499,882	18	34	38
259	1,499,869	18	34	37
260	1,499,850	18	34	38
261	1,499,827	20	35	38
262	1,499,809	18	34	38
263	1,499,783	18	34	38
264	1,499,768	18	34	38
265	1,499,746	17	34	38
266	1,499,719	18	34	38
267	1,499,694	18	34	38
268	1,499,673	17	34	38
269	1,499,653	18	34	38
270	1,499,631	2	34	37
271	1,499,614	17	34	38
272	1,499,217	17	34	37
273	1,498,771	17	34	37
274	1,497,253	16	34	37
275	1,435,024	15	33	37
276	1,053,560	8	19	25

75%ile	95%ile
34	34
34	34
34	34
34	34
34	34

75%ile	95%ile
38	38
38	38
38	38
38	38
38	38
38	38
38	38
38	38
38	38
38	38
38	38
38	38
38	38
38	38
38	38
38	38
38	38
38	38
38	38
38	38
38	38
38	38
38	38
38	38
38	38
38	38
38	38
38	38
38	38
38	38
38	38
38	38
38	38
38	38
38	38
38	38
38	38

75%ile	95%ile
38	38
38	38
38	38
38	38
38	38
38	38
38	38
38	38
38	38
38	38
38	38
38	38
38	38
38	38
38	38
38	38
38	38
38	38
38	38
38	38
38	38
38	38
38	38
38	38
38	38
38	38
38	38
38	38
38	38
38	38
38	38
38	38
38	38
38	38
38	38
38	38
38	38

75%ile	95%ile
38	38
38	38
38	38
38	38
38	38
38	38
38	38
38	38
38	38
38	38
38	38
38	38
38	38
38	38
38	38
38	38
38	38
38	38
38	38
38	38
38	38
38	38
38	38
38	38
38	38
38	38
38	38
38	38
38	38
38	38
38	38
38	38
38	38
38	38
38	38
38	38
38	38

75%ile	95%ile
38	38
38	38
38	38
38	38
38	38
38	38
38	38
38	38
38	38
38	38
38	38
38	38
38	38
38	38
38	38
38	38
38	38
38	38
38	38
38	38
38	38
38	38
38	38
38	38
38	38
38	38
38	38
38	38
38	38
38	38
38	38
38	38
38	38
38	38
38	38
38	38
38	38

75%ile	95%ile
38	38
38	38
38	38
38	38
38	38
38	38
38	38
38	38
38	38
38	38
38	38
38	38
38	38
38	38
38	38
38	38
38	38
38	38
38	38
38	38
38	38
38	38
38	38
38	38
38	38
38	38
38	38
38	38
38	38
38	38
38	38
38	38
38	38
38	38
38	38
38	38
38	38

75%ile	95%ile
38	38
38	38
38	38
38	38
38	38
38	38
38	38
38	38
38	38
38	38
38	38
38	38
38	38
38	38
38	38
38	38
38	38
38	38
38	38
38	38
38	38
38	38
38	38
38	38
38	38
38	38
38	38
38	38
38	38
38	38
38	38
38	38
38	38
38	38
38	38
38	38
38	38

75%ile	95%ile
38	38
38	38
38	38
38	38
38	38
38	38
38	38
38	38
38	38
38	38
38	38
38	38
38	38
38	38
38	38
38	38
38	38
38	38
38	38
38	38
38	38
38	38
38	38
38	38
38	38
38	38
38	38
38	38
38	38
38	38
38	38
38	38
38	38
38	38
38	38
38	38
38	38

75%ile	95%ile
38	38
38	38
38	38
38	38
38	38
38	38
38	38
38	38
38	38
38	38
38	38
35	38

R2

Summarizes the base-quality distribution along the base positions for R2 reads.
coverage: total number of quality values observed at that base position
median & percentiles: median & percentiles of quality scores observed at that base position

base position	coverage	5%ile	25%ile	Median
1	1,514,316	21	34	34
2	1,514,316	23	34	34
3	1,514,316	23	34	34
4	1,514,316	23	34	34
5	1,514,316	23	34	34
6	1,514,316	26	37	38
7	1,514,316	26	37	38
8	1,514,316	27	37	38
9	1,514,316	27	37	38
10	1,514,316	25	37	38
11	1,514,316	26	37	38
12	1,514,316	27	37	38
13	1,514,316	26	37	38
14	1,514,316	24	37	38
15	1,514,316	25	37	38
16	1,514,316	23	37	38
17	1,514,316	27	37	38
18	1,514,316	26	37	38

base position	coverage	5%ile	25%ile	Median
19	1,514,316	26	37	38
20	1,514,316	26	37	38
21	1,514,316	26	37	38
22	1,514,316	25	37	38
23	1,514,316	27	37	38
24	1,514,316	27	37	38
25	1,514,316	26	37	38
26	1,514,316	27	37	38
27	1,514,316	27	37	38
28	1,514,316	27	37	38
29	1,514,316	25	37	38
30	1,514,316	27	37	38
31	1,514,316	26	37	38
32	1,514,316	27	37	38
33	1,514,316	26	37	38
34	1,514,316	25	37	38
35	1,514,316	23	37	38
36	1,514,316	24	37	38
37	1,514,315	23	37	38
38	1,514,315	24	37	38
39	1,514,315	25	37	38
40	1,514,314	23	37	38
41	1,514,314	24	37	38
42	1,514,310	24	37	38
43	1,514,284	23	37	38
44	1,514,266	24	37	38
45	1,514,252	24	37	38
46	1,514,226	23	36	38
47	1,514,190	23	36	38
48	1,514,148	23	37	38
49	1,514,107	24	37	38
50	1,514,081	25	37	38
51	1,514,050	24	37	38
52	1,514,008	25	37	38
53	1,513,983	24	37	38
54	1,513,956	24	37	38
55	1,513,938	24	37	38

base position	coverage	5%ile	25%ile	Median
56	1,513,926	25	37	38
57	1,513,923	25	37	38
58	1,513,916	24	37	38
59	1,513,914	24	37	38
60	1,513,914	24	37	38
61	1,513,912	23	37	38
62	1,513,911	24	37	38
63	1,513,911	23	37	38
64	1,513,911	23	37	38
65	1,513,911	23	37	38
66	1,513,907	24	37	38
67	1,513,904	24	37	38
68	1,513,903	24	37	38
69	1,513,902	24	37	38
70	1,513,899	24	37	38
71	1,513,897	23	37	38
72	1,513,897	23	37	38
73	1,513,896	23	37	38
74	1,513,895	23	37	38
75	1,513,895	22	37	38
76	1,513,895	22	36	38
77	1,513,895	22	36	38
78	1,513,895	22	36	38
79	1,513,894	21	36	38
80	1,513,894	23	36	38
81	1,513,894	23	37	38
82	1,513,894	23	37	38
83	1,513,894	22	36	38
84	1,513,894	23	37	38
85	1,513,894	11	36	38
86	1,513,894	23	36	38
87	1,513,894	22	36	38
88	1,513,894	22	36	38
89	1,513,894	22	36	38
90	1,513,894	23	37	38
91	1,513,894	22	36	38
92	1,513,894	23	37	38

base position	coverage	5%ile	25%ile	Median
93	1,513,894	22	36	38
94	1,513,894	23	37	38
95	1,513,894	22	37	38
96	1,513,894	23	37	38
97	1,513,894	23	37	38
98	1,513,894	22	37	38
99	1,513,894	22	36	38
100	1,513,894	22	37	38
101	1,513,894	23	37	38
102	1,513,893	22	36	38
103	1,513,892	22	37	38
104	1,513,892	22	36	38
105	1,513,891	22	36	38
106	1,513,890	22	36	38
107	1,513,890	22	36	38
108	1,513,890	22	36	38
109	1,513,890	21	36	38
110	1,513,890	22	36	38
111	1,513,890	21	36	38
112	1,513,890	21	36	38
113	1,513,889	22	36	38
114	1,513,889	22	36	38
115	1,513,889	22	36	38
116	1,513,879	22	36	38
117	1,513,865	22	36	38
118	1,513,837	22	36	38
119	1,513,779	21	36	38
120	1,513,739	21	36	38
121	1,513,677	20	36	38
122	1,513,606	21	36	38
123	1,513,534	21	36	38
124	1,513,438	19	35	38
125	1,513,308	19	35	38
126	1,513,195	21	36	38
127	1,513,069	20	36	38
128	1,512,948	20	36	38
129	1,512,829	19	35	38

base position	coverage	5%ile	25%ile	Median
130	1,512,736	19	35	38
131	1,512,663	19	35	38
132	1,512,583	19	35	38
133	1,512,524	19	36	38
134	1,512,467	19	35	38
135	1,512,432	19	35	38
136	1,512,405	19	35	38
137	1,512,395	19	35	38
138	1,512,384	18	35	38
139	1,512,378	19	35	38
140	1,512,368	18	35	38
141	1,512,363	18	35	38
142	1,512,357	18	35	38
143	1,512,350	16	34	38
144	1,512,347	15	34	38
145	1,512,341	16	34	38
146	1,512,341	15	34	38
147	1,512,339	17	34	38
148	1,512,338	17	35	38
149	1,512,336	14	34	38
150	1,512,336	18	35	38
151	1,512,336	15	34	37
152	1,512,336	16	34	38
153	1,512,316	14	34	38
154	1,512,315	16	34	38
155	1,512,315	16	34	37
156	1,512,315	15	34	37
157	1,512,312	11	34	37
158	1,512,312	11	34	37
159	1,512,307	11	34	37
160	1,512,299	14	34	37
161	1,512,284	11	34	37
162	1,512,252	11	34	37
163	1,512,196	15	34	37
164	1,512,131	16	34	37
165	1,512,041	11	34	37
166	1,511,915	11	34	37

base position	coverage	5%ile	25%ile	Median
167	1,511,761	11	34	37
168	1,511,581	11	34	37
169	1,511,370	11	34	37
170	1,511,138	11	34	37
171	1,510,869	11	34	37
172	1,510,568	11	32	37
173	1,510,248	11	34	37
174	1,509,928	11	34	37
175	1,509,648	11	32	37
176	1,509,392	11	31	37
177	1,509,150	10	30	37
178	1,508,958	10	31	37
179	1,508,795	10	29	36
180	1,508,668	10	28	36
181	1,508,525	10	29	37
182	1,508,456	10	30	37
183	1,508,397	10	28	36
184	1,508,353	10	31	37
185	1,508,340	10	30	37
186	1,508,328	10	29	37
187	1,508,325	10	29	37
188	1,508,319	10	29	37
189	1,508,318	10	30	37
190	1,508,312	10	30	37
191	1,508,247	10	29	37
192	1,508,179	10	29	37
193	1,508,136	11	30	37
194	1,507,653	11	30	37
195	1,507,170	11	30	37
196	1,506,852	11	29	37
197	1,506,588	10	29	37
198	1,506,033	11	30	37
199	1,505,459	10	29	37
200	1,505,098	11	30	37
201	1,504,758	10	29	37
202	1,504,658	11	30	37
203	1,504,531	10	28	37

base position	coverage	5%ile	25%ile	Median
204	1,504,428	10	28	37
205	1,504,355	10	28	37
206	1,504,313	9	26	35
207	1,504,312	9	26	35
208	1,504,312	9	25	35
209	1,504,309	9	24	33
210	1,504,299	9	24	33
211	1,504,040	9	24	33
212	1,503,814	9	24	34
213	1,503,685	9	24	33
214	1,503,553	9	24	33
215	1,503,283	9	23	32
216	1,502,687	9	24	34
217	1,502,227	9	23	33
218	1,501,838	9	25	34
219	1,501,660	9	24	33
220	1,501,306	9	23	32
221	1,500,920	9	24	34
222	1,500,675	9	23	32
223	1,500,474	8	22	31
224	1,500,473	8	21	31
225	1,500,469	8	21	30
226	1,500,465	8	20	30
227	1,500,465	8	20	30
228	1,500,464	8	19	29
229	1,500,460	8	20	29
230	1,500,457	8	19	29
231	1,500,452	8	19	29
232	1,500,441	8	20	29
233	1,500,431	8	20	29
234	1,500,422	8	20	29
235	1,500,412	8	19	28
236	1,500,405	8	19	28
237	1,500,393	8	19	27
238	1,500,381	8	20	29
239	1,500,367	8	20	29
240	1,500,360	8	19	29

base position	coverage	5%ile	25%ile	Median
241	1,500,352	8	19	29
242	1,500,351	7	18	27
243	1,500,344	7	18	27
244	1,500,340	8	19	28
245	1,500,336	8	19	27
246	1,500,332	8	18	27
247	1,500,327	7	18	27
248	1,500,327	8	18	27
249	1,500,326	8	18	27
250	1,500,324	7	17	26
251	1,500,324	7	17	26
252	1,500,321	7	16	25
253	1,500,310	7	17	25
254	1,500,308	7	17	25
255	1,500,301	7	17	25
256	1,500,291	7	17	25
257	1,500,280	7	17	25
258	1,500,271	7	16	24
259	1,500,264	7	16	25
260	1,500,249	7	16	25
261	1,500,232	7	16	24
262	1,500,210	7	16	24
263	1,500,194	7	16	24
264	1,500,170	7	15	24
265	1,500,157	7	16	24
266	1,500,135	7	15	24
267	1,500,110	7	15	23
268	1,500,089	7	15	22
269	1,500,071	7	15	22
270	1,500,052	7	15	22
271	1,500,023	7	14	22
272	1,499,962	7	13	21
273	1,499,647	7	13	22
274	1,495,543	7	13	21
275	1,444,002	7	13	21

base position	coverage	5%ile	25%ile	Median
276	1,241,911	7	8	13

75%ile	95%ile
34	34
34	34
34	34
34	34
34	34
38	38
38	38
38	38
38	38
38	38
38	38
38	38
38	38
38	38
38	38
38	38
38	38
38	38
38	38
38	38
38	38
38	38
38	38
38	38
38	38
38	38
38	38
38	38
38	38
38	38
38	38
38	38
38	38
38	38

75%ile	95%ile
38	38
38	38
38	38
38	38
38	38
38	38
38	38
38	38
38	38
38	38
38	38
38	38
38	38
38	38
38	38
38	38
38	38
38	38
38	38
38	38
38	38
38	38
38	38
38	38
38	38
38	38
38	38
38	38
38	38
38	38
38	38
38	38
38	38
38	38
38	38
38	38
38	38

75%ile	95%ile
38	38
38	38
38	38
38	38
38	38
38	38
38	38
38	38
38	38
38	38
38	38
38	38
38	38
38	38
38	38
38	38
38	38
38	38
38	38
38	38
38	38
38	38
38	38
38	38
38	38
38	38
38	38
38	38
38	38
38	38
38	38
38	38
38	38
38	38
38	38
38	38
38	38

75%ile	95%ile
38	38
38	38
38	38
38	38
38	38
38	38
38	38
38	38
38	38
38	38
38	38
38	38
38	38
38	38
38	38
38	38
38	38
38	38
38	38
38	38
38	38
38	38
38	38
38	38
38	38
38	38
38	38
38	38
38	38
38	38
38	38
38	38
38	38
38	38
38	38
38	38
38	38

75%ile	95%ile
38	38
38	38
38	38
38	38
38	38
38	38
38	38
38	38
38	38
38	38
38	38
38	38
38	38
38	38
38	38
38	38
38	38
38	38
38	38
38	38
38	38
38	38
38	38
38	38
38	38
38	38
38	38
38	38
38	38
38	38
38	38
37	38
38	38
37	38
37	38
37	38
37	38

75%ile	95%ile
37	38
38	38
37	38
37	38
37	38
37	38
38	38
38	38
37	38
38	38
38	38
38	38
38	38
38	38
38	38
38	38
38	38
38	38
37	38
38	38
37	38
37	38
37	38
37	38
37	38
37	38
37	38
37	37
37	37
37	38
37	37
37	38
37	37
37	38
37	38
37	38
37	38

75%ile	95%ile
37	38
37	38
37	37
37	37
37	37
36	37
36	37
36	37
36	37
36	37
36	37
35	37
36	37
36	37
36	37
35	37
36	37
35	37
36	37
36	37
36	37
36	37
35	37
35	37
36	37
35	37
34	37
34	37
34	37
34	37
33	37
33	37
32	37
32	37
32	37
32	37
33	37

75%ile	95%ile
32	37
31	37
31	37
31	37
31	37
30	37
31	37
30	37
31	37
30	37
30	37
30	37
30	37
30	37
30	37
30	37
30	37
30	37
29	37
19	29

Total

Summarizes the base-quality distribution along the base positions for total reads.
coverage: total number of quality values observed at that base position
median & percentiles: median & percentiles of quality scores observed at that base position

base position	coverage	5%ile	25%ile	Median
1	3,028,632	27	34	34
2	3,028,632	31	34	34
3	3,028,632	31	34	34
4	3,028,632	31	34	34
5	3,028,632	31	34	34
6	3,028,632	31	38	38
7	3,028,632	31	38	38
8	3,028,632	32	38	38
9	3,028,632	31	38	38
10	3,028,632	31	38	38

base position	coverage	5%ile	25%ile	Median
11	3,028,632	31	38	38
12	3,028,632	31	38	38
13	3,028,632	31	38	38
14	3,028,632	31	38	38
15	3,028,632	31	38	38
16	3,028,632	31	38	38
17	3,028,632	31	38	38
18	3,028,632	31	38	38
19	3,028,632	31	38	38
20	3,028,632	31	38	38
21	3,028,632	31	38	38
22	3,028,632	31	38	38
23	3,028,632	31	38	38
24	3,028,632	31	38	38
25	3,028,632	31	38	38
26	3,028,632	31	38	38
27	3,028,632	31	38	38
28	3,028,632	31	38	38
29	3,028,632	31	38	38
30	3,028,632	31	38	38
31	3,028,632	31	38	38
32	3,028,632	31	38	38
33	3,028,632	31	38	38
34	3,028,632	31	38	38
35	3,028,632	31	38	38
36	3,028,631	31	38	38
37	3,028,630	29	37	38
38	3,028,629	31	37	38
39	3,028,628	31	38	38
40	3,028,627	29	37	38
41	3,028,623	28	37	38
42	3,028,593	29	37	38
43	3,028,549	27	37	38
44	3,028,517	27	37	38
45	3,028,476	27	37	38
46	3,028,414	27	37	38
47	3,028,335	27	37	38

base position	coverage	5%ile	25%ile	Median
48	3,028,253	27	37	38
49	3,028,185	27	37	38
50	3,028,127	27	37	38
51	3,028,055	29	37	38
52	3,027,989	31	37	38
53	3,027,937	30	37	38
54	3,027,892	28	37	38
55	3,027,862	27	37	38
56	3,027,847	31	37	38
57	3,027,837	31	38	38
58	3,027,828	30	38	38
59	3,027,826	27	38	38
60	3,027,824	29	37	38
61	3,027,821	27	37	38
62	3,027,820	27	37	38
63	3,027,820	27	37	38
64	3,027,820	27	37	38
65	3,027,816	27	37	38
66	3,027,809	27	37	38
67	3,027,805	27	37	38
68	3,027,803	28	37	38
69	3,027,799	27	37	38
70	3,027,794	27	37	38
71	3,027,792	27	37	38
72	3,027,791	27	37	38
73	3,027,790	27	37	38
74	3,027,789	27	37	38
75	3,027,789	27	37	38
76	3,027,789	27	37	38
77	3,027,789	26	37	38
78	3,027,789	25	37	38
79	3,027,788	25	37	38
80	3,027,788	27	37	38
81	3,027,788	27	37	38
82	3,027,788	27	37	38
83	3,027,788	27	37	38
84	3,027,788	27	37	38

base position	coverage	5%ile	25%ile	Median
85	3,027,788	22	37	38
86	3,027,788	27	37	38
87	3,027,788	25	37	38
88	3,027,788	25	37	38
89	3,027,788	25	37	38
90	3,027,788	27	37	38
91	3,027,788	27	37	38
92	3,027,788	27	37	38
93	3,027,788	27	37	38
94	3,027,788	26	37	38
95	3,027,788	27	37	38
96	3,027,788	27	37	38
97	3,027,788	27	37	38
98	3,027,788	25	37	38
99	3,027,788	27	37	38
100	3,027,787	27	37	38
101	3,027,787	27	37	38
102	3,027,786	25	37	38
103	3,027,784	25	37	38
104	3,027,783	25	37	38
105	3,027,781	25	37	38
106	3,027,780	25	37	38
107	3,027,780	25	37	38
108	3,027,780	25	37	38
109	3,027,780	24	37	38
110	3,027,780	25	37	38
111	3,027,780	25	37	38
112	3,027,779	25	37	38
113	3,027,778	27	37	38
114	3,027,778	25	37	38
115	3,027,767	25	37	38
116	3,027,742	25	37	38
117	3,027,697	25	37	38
118	3,027,615	25	37	38
119	3,027,515	24	37	38
120	3,027,414	24	37	38
121	3,027,281	25	37	38

base position	coverage	5%ile	25%ile	Median
122	3,027,134	24	37	38
123	3,026,967	25	37	38
124	3,026,743	23	37	38
125	3,026,499	23	37	38
126	3,026,261	24	37	38
127	3,026,017	24	37	38
128	3,025,777	24	37	38
129	3,025,564	23	37	38
130	3,025,398	23	37	38
131	3,025,246	23	37	38
132	3,025,104	23	37	38
133	3,024,989	24	37	38
134	3,024,894	23	37	38
135	3,024,834	23	37	38
136	3,024,797	23	37	38
137	3,024,776	23	37	38
138	3,024,758	23	37	38
139	3,024,743	23	37	38
140	3,024,728	23	37	38
141	3,024,718	23	37	38
142	3,024,704	23	37	38
143	3,024,694	22	36	38
144	3,024,685	22	36	38
145	3,024,679	23	36	38
146	3,024,677	22	36	38
147	3,024,674	22	36	38
148	3,024,672	23	36	38
149	3,024,670	22	36	38
150	3,024,670	23	37	38
151	3,024,670	22	36	38
152	3,024,651	22	36	38
153	3,024,630	22	36	38
154	3,024,629	22	36	38
155	3,024,629	22	36	38
156	3,024,626	22	36	38
157	3,024,623	21	36	38
158	3,024,618	21	36	38

base position	coverage	5%ile	25%ile	Median
159	3,024,605	21	36	38
160	3,024,584	21	36	38
161	3,024,536	21	36	38
162	3,024,448	22	36	38
163	3,024,325	21	36	38
164	3,024,168	22	36	38
165	3,023,948	22	36	38
166	3,023,667	21	36	38
167	3,023,333	21	36	38
168	3,022,938	20	36	38
169	3,022,498	21	36	38
170	3,021,994	20	36	38
171	3,021,427	20	36	38
172	3,020,808	20	35	38
173	3,020,165	20	36	38
174	3,019,563	20	35	38
175	3,019,027	19	35	38
176	3,018,527	18	35	38
177	3,018,090	18	34	37
178	3,017,734	18	35	38
179	3,017,447	17	34	37
180	3,017,172	16	34	37
181	3,016,958	17	34	37
182	3,016,830	17	34	37
183	3,016,726	17	34	37
184	3,016,669	18	35	38
185	3,016,643	18	34	37
186	3,016,628	17	34	37
187	3,016,621	17	34	37
188	3,016,613	16	34	37
189	3,016,605	18	34	37
190	3,016,531	17	34	37
191	3,016,395	17	34	37
192	3,016,277	17	34	38
193	3,015,731	17	34	38
194	3,014,761	17	34	38
195	3,013,932	17	34	38

base position	coverage	5%ile	25%ile	Median
196	3,013,335	16	34	38
197	3,012,499	17	34	38
198	3,011,359	16	34	38
199	3,010,395	16	34	38
200	3,009,683	16	34	38
201	3,009,241	16	34	37
202	3,009,008	16	34	38
203	3,008,776	15	34	37
204	3,008,594	15	34	37
205	3,008,474	14	34	37
206	3,008,431	14	32	37
207	3,008,430	14	33	37
208	3,008,428	11	32	37
209	3,008,409	11	31	37
210	3,008,136	12	30	37
211	3,007,642	12	30	37
212	3,007,271	11	31	37
213	3,007,006	11	31	37
214	3,006,594	11	30	37
215	3,005,702	11	30	37
216	3,004,606	11	31	37
217	3,003,721	11	30	37
218	3,003,151	13	32	37
219	3,002,604	11	31	37
220	3,001,850	10	29	37
221	3,001,190	11	31	37
222	3,000,737	10	29	37
223	3,000,535	10	28	37
224	3,000,532	10	28	37
225	3,000,527	10	28	36
226	3,000,523	9	27	36
227	3,000,521	10	27	36
228	3,000,516	9	26	36
229	3,000,512	10	26	36
230	3,000,505	9	26	36
231	3,000,489	9	26	36
232	3,000,469	9	26	36

base position	coverage	5%ile	25%ile	Median
233	3,000,448	9	26	36
234	3,000,430	9	26	36
235	3,000,413	9	26	36
236	3,000,402	9	25	36
237	3,000,387	9	25	36
238	3,000,366	9	26	36
239	3,000,346	9	26	36
240	3,000,331	9	26	36
241	3,000,320	9	26	36
242	3,000,311	8	25	35
243	3,000,300	9	25	36
244	3,000,293	9	25	36
245	3,000,285	9	25	36
246	3,000,277	8	24	35
247	3,000,272	8	24	35
248	3,000,271	8	24	35
249	3,000,268	8	24	34
250	3,000,265	8	22	34
251	3,000,262	8	22	34
252	3,000,249	8	22	34
253	3,000,233	8	22	34
254	3,000,225	8	22	34
255	3,000,210	8	22	34
256	3,000,190	8	23	34
257	3,000,169	8	22	33
258	3,000,153	8	21	33
259	3,000,133	8	21	33
260	3,000,099	8	22	33
261	3,000,059	8	21	33
262	3,000,019	8	21	33
263	2,999,977	8	21	33
264	2,999,938	8	21	33
265	2,999,903	8	21	33
266	2,999,854	8	21	33
267	2,999,804	8	21	32
268	2,999,762	8	20	32
269	2,999,724	8	20	32

base position	coverage	5%ile	25%ile	Median
270	2,999,683	7	19	30
271	2,999,637	8	20	32
272	2,999,179	8	19	32
273	2,998,418	8	20	32
274	2,992,796	8	19	31
275	2,879,026	8	19	30
276	2,295,471	7	9	19

75%ile	95%ile
34	34
34	34
34	34
34	34
34	34
38	38
38	38
38	38
38	38
38	38
38	38
38	38
38	38
38	38
38	38
38	38
38	38
38	38
38	38
38	38
38	38
38	38
38	38
38	38
38	38
38	38
38	38
38	38
38	38

75%ile	95%ile
38	38
38	38
38	38
38	38
38	38
38	38
38	38
38	38
38	38
38	38
38	38
38	38
38	38
38	38
38	38
38	38
38	38
38	38
38	38
38	38
38	38
38	38
38	38
38	38
38	38
38	38
38	38
38	38
38	38
38	38
38	38
38	38
38	38
38	38
38	38
38	38
38	38

75%ile	95%ile
38	38
38	38
38	38
38	38
38	38
38	38
38	38
38	38
38	38
38	38
38	38
38	38
38	38
38	38
38	38
38	38
38	38
38	38
38	38
38	38
38	38
38	38
38	38
38	38
38	38
38	38
38	38
38	38
38	38
38	38
38	38
38	38
38	38
38	38
38	38
38	38
38	38

75%ile	95%ile
38	38
38	38
38	38
38	38
38	38
38	38
38	38
38	38
38	38
38	38
38	38
38	38
38	38
38	38
38	38
38	38
38	38
38	38
38	38
38	38
38	38
38	38
38	38
38	38
38	38
38	38
38	38
38	38
38	38
38	38
38	38
38	38
38	38
38	38
38	38
38	38
38	38

75%ile	95%ile
38	38
38	38
38	38
38	38
38	38
38	38
38	38
38	38
38	38
38	38
38	38
38	38
38	38
38	38
38	38
38	38
38	38
38	38
38	38
38	38
38	38
38	38
38	38
38	38
38	38
38	38
38	38
38	38
38	38
38	38
38	38
38	38
38	38
38	38
38	38
38	38
38	38

75%ile	95%ile
38	38
38	38
38	38
38	38
38	38
38	38
38	38
38	38
38	38
38	38
38	38
38	38
38	38
38	38
38	38
38	38
38	38
38	38
38	38
38	38
38	38
38	38
38	38
38	38
38	38
38	38
38	38
38	38
38	38
38	38
38	38
38	38
38	38
38	38
38	38
38	38
38	38

75%ile	95%ile
38	38
38	38
38	38
38	38
38	38
38	38
38	38
38	38
38	38
38	38
38	38
38	38
38	38
38	38
38	38
38	38
38	38
38	38
38	38
38	38
38	38
38	38
38	38
38	38
38	38
38	38
38	38
38	38
38	38
38	38
38	38
38	38
38	38
38	38
38	38
38	38
37	38

75%ile	95%ile
37	38
38	38
38	38
38	38
38	38
38	38
38	38
38	38
37	38
38	38
38	38
38	38
38	38
38	38
38	38
38	38
38	38
38	38
38	38
37	38
38	38
37	38
37	38
37	38
37	38
27	37

4. Over-representation analyses

4.1 Enriched 5-mers

R1

Summarizes the five most-overrepresented 5-mers for R1 reads. The over-representation of a 5-mer is calculated as the ratio of the expected and observed 5-mer frequency. The expected frequency is

calculated as product of the empirical nucleotide probabilities that make up the k-mer. (5-mers that contain ambiguous bases are ignored)
coverage: number of 5-mers observed at that position
%: number of times a 5-mer has been observed at that position normalized to all 5-mers observed at that position

base position	coverage	% CCTAC	% CCCTT	% CGGCC
1	1,513,913	12.14	0.00	0.00
2	1,514,315	0.01	0.00	0.00
3	1,514,315	0.00	0.00	0.00
4	1,514,315	0.00	0.00	0.00
5	1,514,315	0.01	0.00	0.00
6	1,514,315	1.15	0.00	0.00
7	1,514,316	0.00	0.00	0.00
8	1,514,316	0.00	0.00	0.00
9	1,514,315	0.00	0.00	0.01
10	1,514,315	0.00	0.00	0.00
11	1,514,315	0.00	0.00	0.00
12	1,514,313	0.00	0.00	0.00
13	1,514,313	0.00	0.27	0.00
14	1,514,181	0.00	0.41	0.00
15	1,514,181	0.00	0.40	0.00
16	1,514,181	0.00	0.42	0.00
17	1,514,183	0.00	0.45	0.00
18	1,514,183	0.00	0.47	0.00
19	1,514,316	0.00	0.45	0.00
20	1,514,315	0.00	0.39	0.00
21	1,514,315	0.00	0.46	0.00
22	1,514,305	0.00	0.47	0.00
23	1,514,305	0.00	0.42	0.00
24	1,514,304	0.00	0.43	0.00
25	1,514,124	0.00	0.13	0.00
26	1,514,121	0.00	0.00	0.00
27	1,514,123	0.00	0.00	0.00
28	1,514,081	0.01	0.00	0.00
29	1,514,063	0.40	0.00	0.00
30	1,514,201	0.37	0.00	0.00
31	1,514,201	0.41	0.00	0.00
32	1,514,199	0.43	0.01	0.00

base position	coverage	% CCTAC	% CCCTT	% CGGCC
33	1,514,203	0.48	0.75	0.00
34	1,514,250	0.43	0.72	0.00
35	1,514,249	0.46	0.80	0.00
36	1,514,249	0.42	0.79	0.00
37	1,514,264	0.41	0.81	0.00
38	1,514,239	0.39	0.72	0.01
39	1,514,224	0.37	0.75	0.01
40	1,514,186	0.36	0.67	0.01
41	1,514,122	0.05	0.68	0.01
42	1,514,086	0.05	0.65	0.01
43	1,514,024	0.04	0.60	0.01
44	1,513,984	0.04	0.57	0.01
45	1,513,957	0.00	0.01	0.01
46	1,513,982	0.00	0.00	0.01
47	1,513,894	0.00	0.00	0.01
48	1,513,887	0.00	0.00	0.01
49	1,513,856	0.00	0.00	0.01
50	1,513,838	0.00	0.00	0.00
51	1,513,826	0.00	0.00	0.00
52	1,513,836	0.00	0.00	0.00
53	1,513,829	0.00	0.00	0.00
54	1,513,870	0.00	0.00	0.00
55	1,513,870	0.00	0.00	0.00
56	1,513,882	0.00	0.00	0.00
57	1,513,855	0.00	0.00	0.00
58	1,513,854	0.00	0.00	0.00
59	1,513,844	0.00	0.00	0.00
60	1,513,844	0.00	0.00	0.00
61	1,513,837	0.13	0.00	0.00
62	1,513,841	0.14	0.00	0.00
63	1,513,830	0.12	0.10	0.00
64	1,513,836	0.14	0.12	0.00
65	1,513,801	0.15	0.11	0.00
66	1,513,796	0.16	0.12	0.00
67	1,513,759	0.14	0.13	0.00
68	1,513,758	0.12	0.13	0.00
69	1,513,758	0.15	0.13	0.00

base position	coverage	% CCTAC	% CCCTT	% CGGCC
70	1,513,760	0.14	0.14	0.00
71	1,513,760	0.13	0.14	0.00
72	1,513,827	0.14	0.13	0.00
73	1,513,803	0.00	0.13	0.01
74	1,513,809	0.00	0.12	0.04
75	1,513,809	0.00	0.03	2.18
76	1,513,359	0.00	0.04	0.01
77	1,513,359	0.00	0.03	0.00
78	1,513,415	0.00	0.02	0.00
79	1,513,378	0.00	0.03	0.00
80	1,513,377	0.00	0.01	0.00
81	1,513,779	0.00	0.01	0.00
82	1,513,779	0.00	0.01	0.01
83	1,513,560	0.00	0.01	0.00
84	1,513,560	0.00	0.01	0.00
85	1,513,563	0.00	0.01	0.00
86	1,513,571	0.00	0.02	0.00
87	1,513,571	0.00	0.01	0.00
88	1,513,821	0.00	0.02	0.00
89	1,513,821	0.00	0.01	0.00
90	1,513,821	0.00	0.02	0.00
91	1,513,891	0.00	0.00	0.00
92	1,513,891	0.00	0.00	0.00
93	1,513,884	0.00	0.00	0.00
94	1,513,884	0.00	0.01	0.00
95	1,513,887	0.00	0.00	0.00
96	1,513,886	0.00	0.00	0.00
97	1,513,885	0.00	0.00	0.00
98	1,513,887	0.00	0.00	0.00
99	1,513,886	0.00	0.00	0.00
100	1,513,885	0.00	0.00	0.00
101	1,513,881	0.00	0.00	0.00
102	1,513,857	0.00	0.00	0.00
103	1,513,857	0.00	0.00	0.00
104	1,513,857	0.00	0.00	0.00
105	1,513,857	0.00	0.00	0.00
106	1,513,857	0.00	0.00	0.00

base position	coverage	% CCTAC	% CCCTT	% CGGCC
107	1,513,877	0.00	0.01	0.19
108	1,513,876	0.00	0.43	0.22
109	1,513,866	0.00	0.48	0.20
110	1,513,825	0.00	0.66	0.28
111	1,513,814	0.00	0.74	0.40
112	1,513,799	0.00	0.76	0.42
113	1,513,768	0.00	0.79	0.40
114	1,513,704	0.00	0.78	0.37
115	1,513,699	0.00	0.70	0.49
116	1,513,605	0.00	0.82	0.44
117	1,513,534	0.00	0.73	0.44
118	1,513,458	0.00	0.76	0.45
119	1,513,367	0.05	0.74	0.23
120	1,513,250	0.42	0.27	0.21
121	1,513,170	0.42	0.27	0.20
122	1,513,045	0.47	0.26	0.14
123	1,512,924	0.65	0.30	0.06
124	1,512,809	0.66	0.33	0.05
125	1,512,717	0.67	0.32	0.04
126	1,512,644	3.68	0.32	0.04
127	1,512,565	0.60	0.38	0.00
128	1,512,482	0.62	0.33	0.00
129	1,512,426	0.56	2.06	0.00
130	1,512,388	0.25	0.74	0.00
131	1,512,363	0.21	0.80	0.00
132	1,512,357	0.22	0.75	0.00
133	1,512,373	0.14	0.76	0.00
134	1,512,366	0.17	0.43	0.00
135	1,512,362	0.05	0.37	0.00
136	1,512,357	0.04	0.36	0.00
137	1,512,342	0.03	0.38	0.13
138	1,512,330	0.04	0.35	0.14
139	1,512,319	0.00	0.02	0.15
140	1,512,293	0.00	0.02	0.15
141	1,512,264	0.00	0.02	0.15
142	1,512,209	0.00	0.02	0.15
143	1,512,102	0.00	0.01	0.15

base position	coverage	% CCTAC	% CCCTT	% CGGCC
144	1,512,100	0.00	0.01	0.13
145	1,512,100	0.00	0.00	0.14
146	1,512,100	0.00	0.00	0.13
147	1,512,093	0.00	0.00	0.12
148	1,512,105	0.00	0.00	0.12
149	1,512,082	0.00	0.00	0.01
150	1,512,082	0.00	0.01	0.01
151	1,512,082	0.00	0.00	0.00
152	1,512,079	0.05	0.00	0.00
153	1,512,196	0.05	0.00	0.00
154	1,512,297	0.06	0.00	0.00
155	1,512,294	0.06	0.00	0.00
156	1,512,271	0.06	0.01	0.00
157	1,512,167	0.05	0.01	0.00
158	1,512,031	0.05	0.00	0.00
159	1,511,884	0.05	0.01	0.00
160	1,511,792	0.05	0.01	0.01
161	1,511,610	0.05	0.00	0.00
162	1,511,466	0.04	0.00	0.00
163	1,511,351	0.04	0.01	0.01
164	1,511,301	0.00	0.00	0.56
165	1,511,065	0.00	0.00	0.54
166	1,510,849	0.00	0.00	0.65
167	1,510,547	0.10	0.00	0.67
168	1,510,209	0.16	0.00	0.67
169	1,509,882	0.22	0.00	0.61
170	1,509,601	0.23	0.00	0.97
171	1,509,345	0.24	0.00	0.91
172	1,509,102	0.23	0.00	0.93
173	1,508,930	0.57	0.00	0.91
174	1,508,776	0.78	0.00	0.88
175	1,508,652	0.80	0.00	0.83
176	1,508,504	0.82	0.00	0.41
177	1,508,433	0.82	0.00	0.38
178	1,508,374	0.77	0.00	0.32
179	1,508,326	0.67	0.00	0.30
180	1,508,313	0.61	0.00	0.32

base position	coverage	% CCTAC	% CCCTT	% CGGCC
181	1,508,300	0.54	0.00	0.30
182	1,508,297	0.50	0.02	0.05
183	1,508,293	0.48	0.03	0.04
184	1,508,294	0.46	0.05	0.04
185	1,508,287	0.19	0.06	0.04
186	1,508,219	0.00	0.06	0.04
187	1,508,144	0.00	0.06	0.03
188	1,508,073	0.00	0.06	0.04
189	1,507,570	0.00	0.06	0.05
190	1,507,072	0.00	0.07	0.96
191	1,506,726	0.00	0.06	0.93
192	1,506,447	0.00	0.06	1.05
193	1,505,896	0.00	0.06	1.06
194	1,505,304	0.00	0.04	1.10
195	1,504,929	0.00	0.04	0.98
196	1,504,578	0.00	0.00	1.03
197	1,504,441	0.00	0.00	0.92
198	1,504,307	0.00	0.00	0.92
199	1,504,202	0.00	0.00	0.88
200	1,504,096	0.00	0.00	0.82
201	1,504,049	0.00	0.00	0.80
202	1,504,060	0.00	0.00	0.11
203	1,504,089	0.00	0.00	0.11
204	1,504,087	0.00	0.00	0.05
205	1,504,099	0.00	0.00	0.03
206	1,503,836	0.00	0.00	0.05
207	1,503,601	0.00	0.00	0.97
208	1,503,455	0.00	0.00	1.13
209	1,503,319	0.00	0.00	1.29
210	1,503,026	0.00	0.00	1.35
211	1,502,404	0.00	0.00	1.37
212	1,501,897	0.00	0.00	1.27
213	1,501,472	0.00	0.01	1.29
214	1,501,291	0.00	0.01	1.62
215	1,500,926	0.00	0.01	1.81
216	1,500,484	0.00	0.01	1.80
217	1,500,212	0.00	0.01	1.76

base position	coverage	% CCTAC	% CCCTT	% CGGCC
218	1,500,010	0.00	0.01	1.70
219	1,500,009	0.00	0.00	0.96
220	1,500,016	0.00	0.00	0.78
221	1,500,057	0.00	0.00	0.70
222	1,500,052	0.00	0.00	0.63
223	1,500,050	0.00	0.00	0.59
224	1,500,012	0.01	0.00	0.60
225	1,500,012	0.17	0.00	0.57
226	1,500,008	0.19	0.00	0.24
227	1,499,998	0.18	0.00	0.07
228	1,499,989	0.19	0.00	0.09
229	1,500,017	0.20	0.00	1.00
230	1,500,008	0.20	0.00	1.10
231	1,500,001	0.19	0.01	1.26
232	1,499,997	0.16	0.66	1.32
233	1,499,816	0.21	0.64	1.33
234	1,499,807	0.19	0.76	1.23
235	1,499,801	0.17	0.76	1.28
236	1,499,793	0.18	0.78	1.58
237	1,499,790	0.00	0.69	1.76
238	1,499,959	0.03	0.81	1.76
239	1,499,953	0.05	0.76	1.69
240	1,499,950	0.97	0.75	1.64
241	1,499,946	1.71	0.74	0.91
242	1,499,942	1.84	0.71	0.76
243	1,499,943	1.96	0.79	0.69
244	1,499,935	1.99	0.39	0.62
245	1,499,933	1.89	0.40	0.58
246	1,499,932	1.87	0.36	0.58
247	1,499,931	2.21	0.38	0.54
248	1,499,921	2.35	0.36	0.21
249	1,499,910	2.33	0.34	0.01
250	1,499,904	2.26	0.26	0.01
251	1,499,896	2.17	0.26	0.02
252	1,499,886	1.44	0.25	0.01
253	1,499,876	0.80	0.22	0.01
254	1,499,882	0.72	0.21	0.02

base position	coverage	% CCTAC	% CCCTT	% CGGCC
255	1,499,869	0.63	0.09	0.01
256	1,499,836	0.60	0.01	0.01
257	1,499,813	0.63	0.01	0.01
258	1,499,795	0.61	0.02	0.00
259	1,499,769	0.28	0.00	0.00
260	1,499,754	0.09	0.00	0.00
261	1,499,744	0.08	0.00	0.00
262	1,499,717	0.08	0.00	0.12
263	1,499,672	0.07	0.00	0.15
264	1,499,559	0.08	0.00	0.25
265	1,499,539	0.08	0.00	0.25
266	1,403,132	0.08	0.00	0.26
267	1,403,116	0.06	0.00	0.28
268	1,402,750	0.16	0.00	0.24
269	1,402,446	0.13	0.00	0.28
270	1,401,045	0.13	0.00	0.25
271	1,435,024	0.23	0.00	0.29
272	1,053,558	4.20	0.00	0.34

% TCCTA
0.00
0.00
0.00
0.00
0.00
0.00
0.00
0.00
0.00
0.00
0.00
0.00
0.00
0.00
0.00
0.00
0.00

% TCCTA
0.00
0.01
0.35
0.42
0.40
0.41
0.45
0.39
0.38
0.39
0.40
0.38
0.33
0.33
0.00
0.00
0.00
0.00
0.00
0.00
0.00
0.00
0.00
0.00
0.00
0.00
0.00
0.00
0.00
0.00
0.00
0.00
0.00
0.00
0.00
0.00

% TCCTA
0.00
0.00
0.00
0.00
0.00
0.00
0.00
0.00
0.00
0.00
0.00
0.00
0.00
0.00
0.00
0.00
0.00
0.00
0.00
0.00
0.00
0.00
0.00
0.00
0.00
0.00
0.00
0.00
0.00
0.01
0.01
0.01
0.01
0.01
0.01
0.01
0.01

% TCCTA
0.01
0.00
0.01
0.00
0.01
0.01
0.01
0.23
0.01
0.00
0.00
0.01
0.00
0.00
0.00
0.00
0.00
0.00
0.00
0.00
0.00
0.00
0.00
0.00
0.00
0.00
0.02
0.02
0.02
0.02
0.02
0.02
0.02
0.02
0.02
0.02
0.02

% TCCTA
0.02
0.00
0.00
0.00
0.00
0.00
0.00
0.00
0.01
0.93
0.89
1.05
1.03
1.06
0.94
0.99
0.88
0.89
0.84
0.78
0.74
0.05
0.05
0.00
0.00
0.00
0.00
0.00
0.00
0.00
0.00
0.00
0.00
0.00
0.09
0.26
0.26

% TCCTA
0.30
0.31
0.28
0.25
0.23
0.23
0.23
0.15
0.00
0.00
0.00
0.00
0.00
0.00
0.00
0.00
0.00
0.00
0.00
0.00
0.00
0.00
0.00
0.00
0.00
0.00
0.00
0.00
0.00
0.00
0.00
0.00
0.00
0.00
0.00
0.00
0.00

% TCCTA
0.00
0.00
0.00
0.00
0.00
0.00
0.00
0.00
0.00
0.00
0.00
0.00
0.00
0.00
0.00
0.00
0.00
0.00
0.00
0.00
0.00
0.05
0.05
0.06
0.32
0.35
0.38
0.43
0.44
0.50
0.42
0.47
0.48
0.42
0.33
0.40
1.03

% TCCTA
1.71
1.83
1.87
1.92
1.86
1.78
2.12
2.28
2.24
2.19
2.12
1.39
0.78
0.71
0.62
0.58
0.59
0.56
0.24
0.05
0.05
0.04
0.04
0.03
0.03
0.03
0.03
0.03
0.00
0.00
0.00
0.01
0.00

R2

Summarizes the five most-overrepresented 5-mers for R2 reads. The over-representation of a 5-mer is calculated as the ratio of the expected and observed 5-mer frequency. The expected frequency is

calculated as product of the empirical nucleotide probabilities that make up the k-mer. (5-mers that contain ambiguous bases are ignored)
coverage: number of 5-mers observed at that position
%: number of times a 5-mer has been observed at that position normalized to all 5-mers observed at that position

base position	coverage	% GTAGG	% GGCCG	% TAGGA
1	1,513,310	0.00	0.00	0.00
2	1,514,309	0.00	0.00	0.00
3	1,514,286	0.00	0.00	0.00
4	1,514,286	0.00	0.00	0.00
5	1,514,285	0.00	0.00	0.00
6	1,514,253	0.00	0.00	0.00
7	1,514,253	0.00	0.00	0.00
8	1,514,266	0.00	0.00	0.00
9	1,514,266	0.00	0.02	0.00
10	1,514,256	0.00	0.01	0.00
11	1,514,294	0.00	0.03	0.00
12	1,514,294	0.01	0.01	0.00
13	1,514,294	1.54	0.01	0.01
14	1,514,287	1.51	0.01	1.53
15	1,514,290	1.22	0.03	1.50
16	1,514,290	1.34	0.01	1.21
17	1,514,290	1.51	0.00	1.33
18	1,514,289	1.72	0.00	1.50
19	1,514,311	1.66	0.00	1.71
20	1,514,310	1.21	0.00	1.66
21	1,514,300	1.35	0.00	1.21
22	1,514,300	1.25	0.00	1.35
23	1,514,262	0.01	0.01	1.24
24	1,514,261	0.00	1.10	0.01
25	1,514,261	0.00	1.12	0.00
26	1,514,267	0.00	0.86	0.00
27	1,514,254	0.01	0.96	0.00
28	1,514,258	0.00	1.07	0.00
29	1,514,260	0.00	1.21	0.00
30	1,514,259	0.40	1.27	0.00
31	1,514,261	0.45	0.95	0.00
32	1,514,310	0.41	1.08	0.00

base position	coverage	% GTAGG	% GGCCG	% TAGGA
33	1,514,303	0.41	1.01	0.00
34	1,514,260	0.46	0.15	0.00
35	1,514,244	0.47	0.12	0.00
36	1,514,168	0.43	0.14	0.00
37	1,514,168	0.40	0.13	0.00
38	1,514,164	0.39	0.17	0.00
39	1,514,138	0.40	0.15	0.00
40	1,514,145	0.00	0.15	0.00
41	1,514,164	0.00	0.14	0.00
42	1,514,136	0.01	0.00	0.00
43	1,514,100	1.28	0.00	0.00
44	1,514,056	1.13	0.00	0.00
45	1,514,013	0.77	0.01	0.00
46	1,513,987	0.63	1.14	0.00
47	1,513,960	1.26	1.19	0.00
48	1,513,894	1.33	1.00	0.00
49	1,513,881	0.86	1.10	0.00
50	1,513,831	0.80	1.24	0.00
51	1,513,813	0.00	1.39	0.00
52	1,513,765	0.00	1.35	0.00
53	1,513,785	0.00	1.01	0.00
54	1,513,778	0.00	1.15	0.00
55	1,513,794	0.00	1.08	0.00
56	1,513,794	0.00	0.13	0.00
57	1,513,863	0.00	0.13	0.00
58	1,513,862	0.00	0.03	0.00
59	1,513,861	0.00	0.03	0.00
60	1,513,861	0.00	0.03	0.00
61	1,513,861	0.00	0.03	0.00
62	1,513,886	0.00	0.03	0.00
63	1,513,895	0.00	0.56	0.00
64	1,513,896	0.00	0.56	0.00
65	1,513,886	0.00	0.42	0.00
66	1,513,883	0.00	0.47	0.00
67	1,513,881	0.00	0.53	0.08
68	1,513,881	0.00	0.59	0.08
69	1,513,875	0.00	0.58	0.05

base position	coverage	% GTAGG	% GGCCG	% TAGGA
70	1,513,880	0.00	0.56	0.04
71	1,513,853	0.00	0.60	0.08
72	1,513,830	0.00	0.59	0.09
73	1,513,830	0.00	0.19	0.06
74	1,513,830	0.00	0.17	0.05
75	1,513,830	0.00	0.17	0.00
76	1,513,833	0.00	0.18	0.00
77	1,513,864	0.00	0.19	0.00
78	1,513,868	0.00	0.18	0.00
79	1,513,860	0.00	0.17	0.00
80	1,513,858	0.00	0.03	0.00
81	1,449,925	0.00	0.03	0.00
82	1,449,919	0.00	0.02	0.00
83	1,449,918	0.00	0.00	0.00
84	1,449,917	0.00	0.00	0.00
85	1,449,888	0.00	0.00	0.00
86	1,513,801	0.04	0.00	0.00
87	1,513,799	0.52	0.00	0.00
88	1,513,800	0.51	0.00	0.00
89	1,513,792	0.41	0.01	0.00
90	1,513,803	0.45	0.64	0.00
91	1,513,798	0.50	0.64	0.00
92	1,513,798	0.58	0.49	0.00
93	1,513,798	0.55	0.54	0.00
94	1,513,839	0.41	0.60	0.00
95	1,513,847	0.45	0.71	0.00
96	1,513,857	0.39	0.67	0.00
97	1,513,855	0.00	0.49	0.00
98	1,513,820	0.00	0.54	0.00
99	1,513,810	0.00	0.50	0.00
100	1,513,805	0.00	0.00	0.00
101	1,513,804	0.00	0.00	0.00
102	1,513,804	0.00	0.00	0.00
103	1,513,769	0.00	0.00	0.00
104	1,513,769	0.00	0.00	0.00
105	1,513,774	0.00	0.00	0.00
106	1,513,774	0.00	0.00	0.00

base position	coverage	% GTAGG	% GGCCG	% TAGGA
107	1,513,774	0.00	0.00	0.00
108	1,513,852	0.00	0.00	0.00
109	1,513,845	0.00	0.00	0.00
110	1,513,743	0.00	0.01	0.00
111	1,513,743	0.00	0.83	0.00
112	1,513,733	0.00	0.74	0.00
113	1,513,719	0.00	0.49	0.00
114	1,513,691	0.00	0.43	0.00
115	1,513,739	0.00	0.82	0.52
116	1,513,699	0.00	0.91	0.54
117	1,513,625	0.00	0.64	0.61
118	1,513,554	0.00	0.59	0.67
119	1,513,482	0.00	0.07	0.82
120	1,513,388	0.00	0.09	0.89
121	1,513,193	0.00	0.09	0.92
122	1,513,079	0.00	0.08	0.72
123	1,512,952	0.00	0.12	0.84
124	1,512,810	0.00	0.08	2.98
125	1,512,691	0.00	0.07	0.43
126	1,512,628	0.00	0.07	0.37
127	1,512,556	0.00	0.04	0.40
128	1,512,470	0.00	0.04	0.34
129	1,512,418	0.00	0.05	0.02
130	1,512,350	0.00	0.04	0.00
131	1,512,340	0.00	0.04	0.00
132	1,512,313	0.00	0.03	0.00
133	1,512,303	0.00	0.01	0.00
134	1,512,292	0.00	0.01	0.00
135	1,512,312	0.00	0.01	0.00
136	1,512,302	0.00	0.01	0.00
137	1,512,301	0.00	0.01	0.00
138	1,512,296	0.00	0.01	0.00
139	1,512,290	0.00	0.01	0.00
140	1,512,250	0.00	0.01	0.00
141	1,512,227	0.00	0.00	0.00
142	1,512,227	0.00	0.00	0.00
143	1,512,265	0.00	0.00	0.00

base position	coverage	% GTAGG	% GGCCG	% TAGGA
144	1,512,262	0.00	0.00	0.00
145	1,512,199	0.00	0.00	0.00
146	1,512,199	0.00	0.00	0.00
147	1,512,197	0.00	0.00	0.00
148	1,512,196	0.00	0.01	0.00
149	1,512,176	0.00	0.00	0.00
150	1,512,249	0.00	0.00	0.00
151	1,512,171	0.02	0.00	0.00
152	1,512,173	0.03	0.00	0.00
153	1,512,176	0.02	0.00	0.00
154	1,512,176	0.02	0.00	0.01
155	1,512,171	0.02	0.00	0.01
156	1,512,212	0.03	0.00	0.01
157	1,512,189	0.02	0.00	0.01
158	1,512,107	0.02	0.00	0.01
159	1,511,632	0.02	0.00	0.02
160	1,511,561	0.02	0.00	0.01
161	1,511,471	0.00	0.00	0.01
162	1,511,342	0.00	0.00	0.01
163	1,511,191	0.04	0.00	0.01
164	1,511,423	0.07	0.00	0.00
165	1,511,222	0.45	0.00	0.03
166	1,510,986	0.39	0.00	0.39
167	1,510,720	0.35	0.00	0.34
168	1,510,434	4.65	0.00	0.24
169	1,510,114	1.63	0.00	1.53
170	1,509,730	1.23	0.00	1.52
171	1,509,485	0.96	0.00	1.14
172	1,509,224	1.80	0.00	0.88
173	1,508,988	1.88	0.00	1.69
174	1,508,797	1.24	0.00	1.80
175	1,508,700	1.10	0.03	1.18
176	1,508,576	0.36	0.18	1.05
177	1,508,439	0.53	0.15	0.35
178	1,508,370	0.46	0.13	0.51
179	1,508,313	0.35	1.38	0.44
180	1,508,269	0.20	1.30	0.33

base position	coverage	% GTAGG	% GGCCG	% TAGGA
181	1,508,282	0.00	0.94	0.19
182	1,508,269	0.00	0.75	0.00
183	1,508,265	0.01	1.57	0.00
184	1,508,273	0.02	1.82	0.01
185	1,508,269	0.96	1.26	0.02
186	1,508,252	0.97	1.09	0.91
187	1,508,187	0.68	0.38	0.81
188	1,508,102	0.65	0.52	0.51
189	1,508,047	1.12	0.46	0.48
190	1,507,564	2.38	0.35	0.93
191	1,507,083	1.91	0.20	2.18
192	1,506,763	1.45	0.00	1.67
193	1,506,502	0.78	0.00	1.24
194	1,505,970	1.33	0.01	0.59
195	1,505,396	1.43	0.01	1.13
196	1,505,035	0.79	0.02	1.21
197	1,504,698	0.76	0.04	0.75
198	1,504,590	0.02	0.33	0.73
199	1,504,416	0.00	0.28	0.03
200	1,504,295	0.00	0.22	0.01
201	1,504,216	0.00	2.60	0.00
202	1,504,177	0.00	2.49	0.00
203	1,504,177	0.00	1.71	0.00
204	1,504,177	0.00	1.39	0.00
205	1,504,263	0.04	2.73	0.00
206	1,504,241	0.04	2.98	0.00
207	1,503,946	0.03	1.95	0.00
208	1,503,200	0.03	1.75	0.00
209	1,503,068	0.04	0.36	0.00
210	1,502,911	0.04	0.51	0.00
211	1,502,645	0.05	0.44	0.00
212	1,502,045	0.03	0.32	0.00
213	1,502,104	0.04	0.19	0.00
214	1,501,714	0.03	0.00	0.00
215	1,501,562	0.01	0.05	0.00
216	1,501,208	0.01	0.05	0.00
217	1,500,838	0.01	0.05	0.00

base position	coverage	% GTAGG	% GGCCG	% TAGGA
218	1,500,591	0.01	1.23	0.00
219	1,500,397	0.01	1.11	0.00
220	1,500,396	0.01	0.73	0.00
221	1,500,394	0.00	0.62	0.00
222	1,500,384	0.00	1.33	0.00
223	1,500,372	0.00	2.79	0.00
224	1,500,371	0.01	2.16	0.00
225	1,500,367	0.06	1.61	0.00
226	1,500,365	0.06	0.85	0.00
227	1,500,365	0.05	1.53	0.00
228	1,500,368	0.05	1.58	0.00
229	1,500,349	0.06	1.00	0.00
230	1,500,306	0.07	0.90	0.00
231	1,500,297	0.11	0.02	0.00
232	1,500,283	0.24	0.00	0.00
233	1,500,291	0.23	0.01	0.00
234	1,500,287	0.20	0.01	0.00
235	1,500,295	0.17	0.01	0.00
236	1,500,289	0.19	0.01	0.00
237	1,500,289	0.21	0.01	0.00
238	1,500,291	0.27	0.02	0.00
239	1,500,274	0.29	0.01	0.00
240	1,500,280	0.28	0.02	0.01
241	1,500,277	0.20	0.04	0.00
242	1,500,270	0.30	0.07	0.02
243	1,500,254	0.31	0.06	0.02
244	1,500,256	0.22	0.04	0.11
245	1,500,243	0.18	0.14	0.10
246	1,500,242	0.27	0.15	0.17
247	1,500,242	0.25	0.11	0.17
248	1,500,228	0.14	0.08	0.20
249	1,500,217	0.14	0.11	0.18
250	1,500,227	0.06	0.09	0.21
251	1,500,211	0.07	0.06	0.18
252	1,500,204	0.04	0.06	0.23
253	1,500,189	0.04	0.01	0.20
254	1,500,182	0.00	0.01	0.19

base position	coverage	% GTAGG	% GGCCG	% TAGGA
255	1,500,175	0.00	0.01	0.16
256	1,500,167	0.00	0.01	0.05
257	1,500,150	0.01	0.01	0.04
258	1,500,142	0.01	0.01	0.00
259	1,500,123	0.01	0.01	0.00
260	1,500,083	0.01	0.01	0.00
261	1,500,059	0.01	0.02	0.00
262	1,500,031	0.01	0.83	0.00
263	1,500,002	0.02	0.73	0.00
264	1,499,977	0.79	0.46	0.00
265	1,499,972	0.74	0.43	0.01
266	1,499,974	0.39	0.81	0.03
267	1,499,916	0.40	1.40	0.03
268	1,499,859	0.78	1.07	0.03
269	1,499,544	0.83	0.93	0.05
270	1,495,448	0.45	0.40	1.08
271	1,443,933	0.51	0.65	0.96
272	1,241,849	0.03	0.58	0.00

% AGGAG
0.00
0.00
0.00
0.00
0.00
0.00
0.00
0.00
0.00
0.00
0.00
0.00
0.00
0.01
1.53
1.51
1.22

% AGGAG
1.33
1.50
1.72
1.66
1.21
1.35
1.24
0.00
0.00
0.00
0.00
0.00
0.00
0.00
0.00
0.00
0.00
0.00
0.00
0.00
0.00
0.00
0.00
0.00
0.00
0.00
0.00
0.00
0.00
0.00
0.00
0.00
0.00
0.00
0.00
0.00
0.00

% AGGAG
0.00
0.00
0.00
0.00
0.00
0.00
0.00
0.00
0.00
0.00
0.00
0.00
0.00
0.00
0.00
0.00
0.00
0.00
0.00
0.00
0.00
0.00
0.00
0.01
0.01
0.01
0.01
0.01
0.01
0.01
0.01
0.01
0.01
0.01
0.01
0.00
0.00

% AGGAG
0.00
0.00
0.00
0.00
0.00
0.00
0.00
0.00
0.00
0.00
0.00
0.00
0.00
0.00
0.00
0.00
0.00
0.00
0.00
0.00
0.00
0.00
0.00
0.00
0.00
0.00
0.00
0.00
0.00
0.00
0.00
0.00
0.02
0.00
0.00
0.00

% AGGAG
0.00
0.00
0.00
0.00
0.00
0.00
0.00
0.00
0.00
0.00
0.00
0.00
0.00
0.00
0.00
0.00
0.00
0.00
0.00
0.00
0.00
0.00
0.00
0.00
0.00
0.00
0.01
0.01
0.01
0.01
0.01
0.02
0.01
0.01
0.01
0.01
0.00

% AGGAG
0.03
0.40
0.59
0.26
1.53
1.54
1.16
0.90
1.70
1.82
1.19
1.06
0.35
0.51
0.44
0.33
0.19
0.00
0.00
0.01
0.02
0.91
0.81
0.51
0.48
0.93
2.13
1.68
1.25
0.59
1.14
1.22
0.75
0.73
0.02
0.00
0.01

% AGGAG
0.01
0.01
0.01
0.01
0.01
0.01
0.01
0.01
0.01
0.00
0.00
0.00
0.00
0.00
0.00
0.00
0.00
0.00
0.00
0.00
0.00
0.00
0.00
0.00
0.00
0.00
0.00
0.00
0.00
0.00
0.00
0.00
0.00
0.00
0.00
0.00
0.00

% AGGAG
0.00
0.00
0.00
0.00
0.00
0.00
0.00
0.00
0.00
0.01
0.00
0.00
0.00
0.00
0.00
0.00
0.00
0.00
0.00
0.00
0.00
0.00
0.00
0.00
0.00
0.00
0.00
0.00
0.00
0.00
0.00
0.00
0.00

4.2 Sequence duplication levels

Summarizes the duplication level distribution. Duplication levels are simply the count of how often a particular sequence has been found.
duplicate count: number of times a sequence has been seen
abs: number of sequences that have been found that many times
%: number of sequences that have been found that many times normalized to the number of unique sequences in R1 / R2

duplicate count	R1: abs	R1: %	R2: abs	R2: %
1	3,759	36.24	6,928	49.60
2	832	8.02	1,138	8.15
3	376	3.63	593	4.25
4	229	2.21	371	2.66
5	142	1.37	292	2.09
6	132	1.27	193	1.38
7	100	0.96	185	1.32
8	88	0.85	151	1.08
9	78	0.75	111	0.79
10	63	0.61	112	0.80
11	63	0.61	114	0.82
12	52	0.50	89	0.64
13	62	0.60	82	0.59
14	47	0.45	69	0.49
15	48	0.46	66	0.47
16	54	0.52	56	0.40
17	49	0.47	41	0.29
18	46	0.44	48	0.34
19	44	0.42	38	0.27
20	38	0.37	49	0.35
21	32	0.31	41	0.29
22	45	0.43	39	0.28
23	43	0.41	35	0.25
24	36	0.35	43	0.31
25	45	0.43	32	0.23
26	20	0.19	29	0.21
27	34	0.33	31	0.22
28	40	0.39	30	0.21

duplicate count	R1: abs	R1: %	R2: abs	R2: %
29	36	0.35	29	0.21
30	41	0.40	33	0.24
31	22	0.21	26	0.19
32	21	0.20	29	0.21
33	22	0.21	23	0.16
34	36	0.35	25	0.18
35	33	0.32	18	0.13
36	29	0.28	16	0.11
37	34	0.33	20	0.14
38	27	0.26	29	0.21
39	20	0.19	14	0.10
40	25	0.24	17	0.12
41	22	0.21	22	0.16
42	33	0.32	14	0.10
43	37	0.36	20	0.14
44	31	0.30	15	0.11
45	21	0.20	15	0.11
46	24	0.23	17	0.12
47	28	0.27	27	0.19
48	23	0.22	18	0.13
49	29	0.28	14	0.10
50	22	0.21	22	0.16
51	27	0.26	13	0.09
52	17	0.16	18	0.13
53	25	0.24	16	0.11
54	16	0.15	19	0.14
55	20	0.19	9	0.06
56	19	0.18	12	0.09
57	20	0.19	21	0.15
58	16	0.15	20	0.14
59	15	0.14	12	0.09
60	19	0.18	19	0.14
61	20	0.19	14	0.10
62	20	0.19	13	0.09
63	19	0.18	10	0.07
64	13	0.13	22	0.16
65	20	0.19	13	0.09

duplicate count	R1: abs	R1: %	R2: abs	R2: %
66	15	0.14	8	0.06
67	19	0.18	13	0.09
68	17	0.16	15	0.11
69	16	0.15	14	0.10
70	20	0.19	11	0.08
71	16	0.15	12	0.09
72	12	0.12	12	0.09
73	21	0.20	12	0.09
74	18	0.17	12	0.09
75	8	0.08	15	0.11
76	20	0.19	9	0.06
77	14	0.13	6	0.04
78	10	0.10	11	0.08
79	23	0.22	8	0.06
80	18	0.17	12	0.09
81	15	0.14	15	0.11
82	14	0.13	16	0.11
83	14	0.13	10	0.07
84	12	0.12	12	0.09
85	13	0.13	12	0.09
86	6	0.06	12	0.09
87	6	0.06	9	0.06
88	5	0.05	13	0.09
89	11	0.11	13	0.09
90	9	0.09	11	0.08
91	10	0.10	14	0.10
92	12	0.12	11	0.08
93	22	0.21	15	0.11
94	12	0.12	12	0.09
95	15	0.14	14	0.10
96	15	0.14	7	0.05
97	7	0.07	11	0.08
98	13	0.13	9	0.06
99	13	0.13	7	0.05
100	15	0.14	8	0.06
105	7	0.07	7	0.05
110	14	0.13	7	0.05

duplicate count	R1: abs	R1: %	R2: abs	R2: %
115	15	0.14	8	0.06
120	10	0.10	13	0.09
125	5	0.05	10	0.07
130	10	0.10	10	0.07
135	9	0.09	10	0.07
140	5	0.05	11	0.08
145	13	0.13	8	0.06
150	11	0.11	6	0.04
155	5	0.05	5	0.04
160	7	0.07	3	0.02
165	6	0.06	5	0.04
170	6	0.06	9	0.06
175	4	0.04	6	0.04
180	6	0.06	3	0.02
185	6	0.06	10	0.07
190	10	0.10	6	0.04
195	9	0.09	3	0.02
200	5	0.05	8	0.06
205	8	0.08	4	0.03
210	2	0.02	1	0.01
215	5	0.05	7	0.05
220	6	0.06	4	0.03
225	4	0.04	4	0.03
230	5	0.05	0	0.00
235	3	0.03	1	0.01
240	5	0.05	4	0.03
245	4	0.04	3	0.02
250	4	0.04	2	0.01
255	8	0.08	8	0.06
260	9	0.09	5	0.04
265	5	0.05	2	0.01
270	6	0.06	2	0.01
275	1	0.01	2	0.01
280	6	0.06	6	0.04
285	4	0.04	4	0.03
290	2	0.02	1	0.01
295	2	0.02	4	0.03

duplicate count	R1: abs	R1: %	R2: abs	R2: %
300	2	0.02	3	0.02
305	1	0.01	4	0.03
310	3	0.03	3	0.02
315	1	0.01	3	0.02
320	0	0.00	4	0.03
325	2	0.02	3	0.02
330	1	0.01	1	0.01
335	3	0.03	1	0.01
340	1	0.01	4	0.03
345	1	0.01	1	0.01
350	4	0.04	3	0.02
355	4	0.04	0	0.00
360	2	0.02	1	0.01
365	2	0.02	1	0.01
370	3	0.03	4	0.03
375	2	0.02	0	0.00
380	1	0.01	3	0.02
385	3	0.03	1	0.01
390	1	0.01	2	0.01
395	2	0.02	0	0.00
400	1	0.01	6	0.04
405	1	0.01	0	0.00
410	0	0.00	1	0.01
420	2	0.02	2	0.01
425	3	0.03	1	0.01
430	1	0.01	3	0.02
435	1	0.01	2	0.01
440	3	0.03	1	0.01
445	3	0.03	0	0.00
450	3	0.03	1	0.01
455	1	0.01	3	0.02
460	0	0.00	1	0.01
465	1	0.01	0	0.00
470	3	0.03	0	0.00
475	2	0.02	1	0.01
480	3	0.03	1	0.01
485	1	0.01	1	0.01

duplicate count	R1: abs	R1: %	R2: abs	R2: %
490	1	0.01	0	0.00
495	1	0.01	1	0.01
500	1	0.01	1	0.01
505	1	0.01	2	0.01
510	2	0.02	1	0.01
515	3	0.03	1	0.01
520	0	0.00	1	0.01
525	1	0.01	2	0.01
530	1	0.01	0	0.00
535	4	0.04	0	0.00
545	1	0.01	4	0.03
550	3	0.03	0	0.00
555	0	0.00	2	0.01
560	0	0.00	1	0.01
565	0	0.00	1	0.01
570	2	0.02	1	0.01
575	2	0.02	0	0.00
580	2	0.02	4	0.03
585	0	0.00	1	0.01
590	1	0.01	0	0.00
600	3	0.03	3	0.02
605	1	0.01	0	0.00
610	1	0.01	0	0.00
615	0	0.00	1	0.01
630	1	0.01	1	0.01
635	2	0.02	0	0.00
640	1	0.01	0	0.00
645	0	0.00	1	0.01
650	2	0.02	0	0.00
655	2	0.02	1	0.01
660	1	0.01	0	0.00
665	0	0.00	2	0.01
670	0	0.00	1	0.01
675	1	0.01	1	0.01
680	4	0.04	0	0.00
685	1	0.01	1	0.01
690	2	0.02	1	0.01

duplicate count	R1: abs	R1: %	R2: abs	R2: %
695	1	0.01	0	0.00
710	3	0.03	0	0.00
715	1	0.01	0	0.00
720	2	0.02	0	0.00
725	1	0.01	0	0.00
730	3	0.03	0	0.00
735	1	0.01	0	0.00
745	1	0.01	0	0.00
750	0	0.00	1	0.01
755	0	0.00	2	0.01
760	1	0.01	0	0.00
765	1	0.01	0	0.00
770	0	0.00	2	0.01
775	2	0.02	0	0.00
795	1	0.01	0	0.00
805	1	0.01	1	0.01
810	1	0.01	0	0.00
815	3	0.03	0	0.00
820	2	0.02	0	0.00
825	2	0.02	0	0.00
830	1	0.01	0	0.00
845	0	0.00	1	0.01
850	1	0.01	0	0.00
860	1	0.01	0	0.00
865	0	0.00	1	0.01
880	3	0.03	1	0.01
885	0	0.00	1	0.01
890	2	0.02	0	0.00
895	0	0.00	1	0.01
905	0	0.00	1	0.01
925	1	0.01	0	0.00
935	0	0.00	1	0.01
940	1	0.01	0	0.00
960	2	0.02	0	0.00
965	1	0.01	0	0.00
975	1	0.01	0	0.00
980	1	0.01	1	0.01

duplicate count	R1: abs	R1: %	R2: abs	R2: %
985	1	0.01	0	0.00
990	1	0.01	0	0.00
1,000	1	0.01	0	0.00
1,030	2	0.02	0	0.00
1,040	1	0.01	0	0.00
1,045	0	0.00	1	0.01
1,050	2	0.02	0	0.00
1,070	1	0.01	1	0.01
1,080	1	0.01	0	0.00
1,095	2	0.02	2	0.01
1,105	1	0.01	0	0.00
1,115	1	0.01	0	0.00
1,120	1	0.01	0	0.00
1,135	1	0.01	0	0.00
1,155	2	0.02	0	0.00
1,160	1	0.01	0	0.00
1,165	0	0.00	1	0.01
1,170	1	0.01	1	0.01
1,200	1	0.01	0	0.00
1,205	0	0.00	2	0.01
1,210	1	0.01	0	0.00
1,225	1	0.01	0	0.00
1,270	0	0.00	1	0.01
1,275	0	0.00	1	0.01
1,280	0	0.00	1	0.01
1,300	2	0.02	0	0.00
1,310	1	0.01	0	0.00
1,320	1	0.01	0	0.00
1,335	1	0.01	0	0.00
1,340	1	0.01	1	0.01
1,350	0	0.00	1	0.01
1,375	0	0.00	1	0.01
1,385	0	0.00	1	0.01
1,405	1	0.01	0	0.00
1,415	1	0.01	0	0.00
1,420	0	0.00	1	0.01
1,425	1	0.01	0	0.00

duplicate count	R1: abs	R1: %	R2: abs	R2: %
1,430	0	0.00	1	0.01
1,440	0	0.00	1	0.01
1,460	2	0.02	0	0.00
1,480	0	0.00	1	0.01
1,510	0	0.00	1	0.01
1,545	1	0.01	0	0.00
1,635	1	0.01	0	0.00
1,700	0	0.00	1	0.01
1,720	0	0.00	1	0.01
1,740	0	0.00	1	0.01
1,780	1	0.01	0	0.00
1,800	1	0.01	0	0.00
1,915	1	0.01	0	0.00
1,940	1	0.01	0	0.00
1,955	0	0.00	1	0.01
1,970	0	0.00	2	0.01
2,020	0	0.00	1	0.01
2,025	0	0.00	2	0.01
2,040	0	0.00	1	0.01
2,055	0	0.00	1	0.01
2,095	0	0.00	1	0.01
2,170	0	0.00	1	0.01
2,265	0	0.00	1	0.01
2,285	0	0.00	1	0.01
2,305	0	0.00	1	0.01
2,325	1	0.01	0	0.00
2,355	0	0.00	1	0.01
2,360	1	0.01	0	0.00
2,365	0	0.00	1	0.01
2,425	0	0.00	1	0.01
2,500	0	0.00	1	0.01
2,530	0	0.00	2	0.01
2,645	0	0.00	1	0.01
2,685	0	0.00	1	0.01
2,710	1	0.01	0	0.00
2,875	0	0.00	1	0.01
2,990	1	0.01	0	0.00

duplicate count	R1: abs	R1: %	R2: abs	R2: %
2,995	0	0.00	1	0.01
3,030	1	0.01	0	0.00
3,215	0	0.00	1	0.01
3,220	0	0.00	1	0.01
3,415	1	0.01	0	0.00
3,480	0	0.00	1	0.01
3,585	0	0.00	1	0.01
3,825	1	0.01	0	0.00
3,855	1	0.01	0	0.00
3,910	0	0.00	1	0.01
4,230	0	0.00	1	0.01
4,280	0	0.00	1	0.01
4,865	0	0.00	1	0.01
4,875	0	0.00	1	0.01
5,065	0	0.00	1	0.01
5,995	1	0.01	0	0.00
7,280	0	0.00	1	0.01
8,865	0	0.00	1	0.01
8,895	0	0.00	1	0.01

4.3 Duplicated sequences

R1

Reports sequences that have been observed more than once for R1 reads.
sequence: the 5'-end of the sequence that has been found multiple times
abs: number of times this sequence has been observed
%: number of times this sequence has been observed normalized to the number of unique sequences

sequence	abs	%
CCTACGGGTGGCTGCAGTGGGGAA TATTGGACAATGGAGGGAACTCTGAT	6,173	0.25
CCTACGGGGGGCTGCAGTGGGGA ATATTGGACAATGGAGGGAACTCTGAT	5,995	0.25
CCTACGGGGGGCAGCAGTGGGGA ATATTGGACAATGGAGGGAACTCTGAT	5,332	0.22
CCTACGGGAGGCTGCAGTGGGGAA TATTGGACAATGGAGGGAACTCTGAT	4,797	0.20

sequence	abs	%
GGCGGACGGGTGAGTAACGCGTAA AGAACTTGCCCTTTAGACTGGGATAA	4,693	0.19
CCTACGGGTGGCAGCAGTGGGGAA TATTGGACAATGGAGGGAACTCTGAT	4,552	0.19
CTAGGCGGACGGGTGAGTAACGCG TAAAGAACTTGCCCTTTAGACTGGGA	4,154	0.17
AGGCGGACGGGTGAGTAACGCGTA AAGAACTTGCCCTTTAGACTGGGATA	3,904	0.16
CCTACGGGCGGCTGCAGTGGGGA ATATTGGACAATGGAGGGAACTCTGAT	3,855	0.16
TAGGCGGACGGGTGAGTAACGCGT AAAGAACTTGCCCTTTAGACTGGGAT	3,825	0.16
CTAACTAGGCGGACGGGTGAGTAA CGCGTAAAGAACTTGCCCTTTAGACT	3,739	0.15
CCTACGGGTGGCTGCAGTGGGGAA TATTGCGCAATGGGGGAAACCCTGAC	3,628	0.15
CCTACGGGTGGCTGCAGTAGGGAA TCTTCCGCAATGGACGAAAGTCTGAC	3,426	0.14
TATCTAGGCGGACGGGTGAGTAAC GCGTAAAGAACTTGCCCTTTAGACTG	3,415	0.14
CCTACGGGGGGCTGCAGTGGGGA ATATTGCGCAATGGGGGAAACCCTGAC	3,382	0.14
GCTAGGCGGACGGGTGAGTAACGC GTAAAGAACTTGCCCTTTAGACTGGG	3,352	0.14
CCTACGGGTGGCTGCAGTGAGGAA TATTGGTCAATGGACGCAAGTCTGAA	3,343	0.14
CCTACGGGAGGCAGCAGTGGGGA ATATTGGACAATGGAGGGAACTCTGAT	3,327	0.14
AGCTATCTAGGCGGACGGGTGAGT AACGCGTAAAGAACTTGCCCTTTAGA	3,296	0.14
ACCTAGGCGGACGGGTGAGTAACG CGTAAAGAACTTGCCCTTTAGACTGG	3,291	0.14
CCTACGGGTGGCTGCAGTAGGGAA TCTTCCACAATGGACGCAAGTCTGAT	3,264	0.13
CCTACGGGGGGCTGCAGTGAGGAA TATTGGTCAATGGACGCAAGTCTGAA	3,164	0.13
CCTACGGGGGGCTGCAGTAGGGAA TCTTCCACAATGGACGCAAGTCTGAT	3,153	0.13
CCTACGGGGGGCTGCAGTAGGGAA TCTTCCGCAATGGACGAAAGTCTGAC	3,096	0.13

R2

Reports sequences that have been observed more than once for R2 reads.
sequence: the 5'-end of the sequence that has been found multiple times
abs: number of times this sequence has been observed
%: number of times this sequence has been observed normalized to the number of unique sequences

sequence	abs	%
CTAGCTTTACCGCGGCTGCTGGCA CGTATTTAGCCGTTGCTTCTTCTGTA	8,895	0.37
TTTACCGCGGCTGCTGGCACGTAT TTAGCCGTTGCTTCTTCTGTAGGTAC	8,865	0.36
TAGCTTTACCGCGGCTGCTGGCAC GTATTTAGCCGTTGCTTCTTCTGTAG	8,427	0.35
CTTTACCGCGGCTGCTGGCACGTA TTTAGCCGTTGCTTCTTCTGTAGGTA	7,747	0.32
CTAGCATTACCGCGGCTGCTGGCA CGTATTTAGCCGTTGCTTCTTCTGTA	7,280	0.30
ATTACCGCGGCTGCTGGCACGTAT TTAGCCGTTGCTTCTTCTGTAGGTAC	6,869	0.28
TAGCATTACCGCGGCTGCTGGCAC GTATTTAGCCGTTGCTTCTTCTGTAG	6,791	0.28
AGCTTTACCGCGGCTGCTGGCACG TATTTAGCCGTTGCTTCTTCTGTAGG	6,719	0.28
CATTACCGCGGCTGCTGGCACGTA TTTAGCCGTTGCTTCTTCTGTAGGTA	6,042	0.25
GCGAGCTTTACCGCGGCTGCTGGC ACGTATTTAGCCGTTGCTTCTTCTGT	5,552	0.23
GACTACCAGGGTATCTAATCCTGTT TGCTCCCCTAGCTTTCGCACTTCAG	5,209	0.21
AGCGAGGATTACCGCGGCTGCTGG CACGTATTTAGCCGTTGCTTCTTCTG	5,151	0.21
GACTACCCGGGTATCTAATCCTGTT TGCTCCCCTAGCTTTCGCACTTCAG	5,065	0.21
GACTACTAGGGTATCTAATCCTGTT TGCTCCCCTAGCTTTCGCACTTCAG	4,973	0.20
GACTACACGGGTATCTAATCCTGTT TGCTCCCCTAGCTTTCGCACTTCAG	4,875	0.20
GACTACTCGGGTATCTAATCCTGTT TGCTCCCCTAGCTTTCGCACTTCAG	4,872	0.20
GACTACAAGGGTATCTAATCCTGTT TGCTCCCCTAGCTTTCGCACTTCAG	4,865	0.20
GCTTTACCGCGGCTGCTGGCACGT ATTTAGCCGTTGCTTCTTCTGTAGGT	4,803	0.20
TTTACCGCGGCTGCTGGCACGTAG TTAGCCGTGACTTTCTAATTGATTAC	4,534	0.19

sequence	abs	%
CTAGCTTTACCGCGGCTGCTGGCA CGTAGTTAGCCGTGACTTTCTAATTG	4,517	0.19
TTTACCGCGGCTGCTGGCACGGAA TTAGCCGGTCCTTATTCATATGGTAC	4,491	0.18
CTAGCTTTACCGCGGCTGCTGGCA CGGAATTAGCCGGTCCTTATTCATAT	4,483	0.18
AGCGAGGTTTACCGCGGCTGCTGG CACGTATTTAGCCGTTGCTTCTTCTG	4,371	0.18
TAGCTTTACCGCGGCTGCTGGCAC GTAGTTAGCCGTGACTTTCTAATTGA	4,328	0.18
